# Comparison of bone augmentation techniques in the mandibular posterior region: A randomized clinical trial protocol

**DOI:** 10.1371/journal.pone.0337304

**Published:** 2026-10-29

**Authors:** Antonio Lanata-Flores, Marcelo Armijos Briones, Pablo Benítez Sellán, Patricia Estefanía Ayala Aguirre

**Affiliations:** School of Dentistry, Universidad de Especialidades Espíritu Santo, Samborondón, Ecuador; Universidade de Trás-os-Montes e Alto Douro: Universidade de Tras-os-Montes e Alto Douro, PORTUGAL

## Abstract

This randomized, statistician-blinded, parallel-group clinical trial will compare the effectiveness of three bone-augmentation techniques applied to the posterior mandible using the cortical shell approach. Eighty-seven participants will be randomly allocated into three groups (autogenous, xenogeneic, or allogeneic cortical shells), each combined with a 1:1 mixture of autogenous bone chips and biomaterial. Participants may contribute one or two eligible posterior mandibular sites; however, the unit of analysis for the primary outcome will be the participant, using a participant-level summary (mean value when two sites are treated). The study is registered at ClinicalTrials.gov (Identifier: NCT06661447). Recruitment has not yet begun. The single primary endpoint is horizontal bone gain (mm) at 6 months, evaluated by cone-beam computed tomography (CBCT). Additional CBCT assessments at 3 and 12 months will be analyzed as secondary longitudinal outcomes. Volumetric and morphometric analyses will be performed through a standardized digital workflow integrating intraoral scanning, CBCT segmentation, and surface registration. Secondary outcomes will include bone density, surgical duration, and postoperative inflammation. Based on a previous CBCT-based randomized clinical trial with a related horizontal ridge-augmentation methodology, assuming a pooled standard deviation of approximately 1.12 mm and a clinically relevant between-group difference of 1.0 mm, 26 evaluable participants per group are required to achieve approximately 81.5% power for each prespecified primary comparison using a conservative two-sided α of 0.025 for sample-size planning. To allow for an anticipated 10% attrition rate, 29 participants will be recruited per group, resulting in a total planned sample of 87 participants. The primary analysis will use an ANCOVA model adjusted for baseline ridge width. This study aims to generate controlled, quantitative evidence on the volumetric and clinical performance of different cortical graft materials for posterior mandibular reconstruction.

Trial registration

ClinicalTrials.gov Identifier: NCT06661447. The registry record will be updated before recruitment begins.

## Introduction

Alveolar bone loss following tooth extraction remains one of the main challenges in oral and maxillofacial rehabilitation. Ridge resorption occurs rapidly, particularly at the buccal aspect and compromises prosthetic and esthetic outcomes [1]. Several augmentation techniques have been described, including guided bone regeneration (GBR), particulate onlay grafts, autogenous block grafts, ridge splitting, sinus-floor elevation, and distraction osteogenesis, each with distinct advantages and drawbacks related to complexity, healing, and stability [2,3].

Among these, the cortical shell technique provides predictable results for horizontal and three-dimensional reconstruction. Thin cortical plates, harvested mainly from the mandibular ramus or symphysis, are fixed to the recipient ridge with microscrews to form a biologic box that contains particulate graft material [4]. This approach combines osteogenic autogenous chips with a xenogeneic scaffold to ensure mechanical stability and volume maintenance [5,6].

However, harvesting autogenous bone can cause donor-site morbidity such as paresthesia, swelling, and limited bone availability [7]. To address these issues, xenogeneic and allogeneic cortical laminae have been introduced as ready-to-use alternatives that replicate the mechanical properties of autogenous bone while avoiding secondary surgery. Xenogeneic grafts exhibit favorable biocompatibility and osteoconductivity but show variable remodeling rates [8]; allogeneic bone grafts offer biologic compatibility but show variable resorption [9].

Evidence remains limited, as most reports are retrospective and rely on two-dimensional imaging. Recent advances in digital volumetric imaging have made it possible to quantify bone remodeling with high precision. Three-dimensional analysis using cone-beam computed tomography (CBCT) combined with specialized software for segmentation and surface registration allows accurate evaluation of volumetric and morphologic changes in augmented areas. This digital workflow enables standardized measurements over time and provides reproducible data for comparing the performance of different grafting materials. Accordingly, this trial will compare the clinical, radiographic, and volumetric performance of autogenous, xenogeneic, and allogeneic cortical shells in a randomized, statistician-blinded, parallel-group design. By combining digital imaging software for standardized CBCT evaluation with three-dimensional analysis tools for quantitative comparison, the study aims to evaluate differences in clinical, radiographic, and volumetric outcomes among autogenous, xenogeneic, and allogeneic cortical shells, including horizontal bone gain, dimensional stability, surgical duration, and postoperative morbidity.

## Materials and methods

### Study design

A randomized, statistician-blinded, parallel-group clinical trial will be conducted at the Universidad de Especialidades Espíritu Santo (UEES), Samborondón, Ecuador, in accordance with the SPIRIT 2025. The study has not yet begun participant recruitment, data collection, or surgical procedures. Recruitment and data collection are expected to begin in October 2026 and conclude in August 2027. Participants will be randomly assigned to one of three groups receiving autogenous, xenogeneic, or allogeneic cortical shells. This parallel allocation avoids potential cross-interference between grafts and allows independent comparison among materials. The trial schedule of enrolment, interventions, and assessments is presented in Fig 1, following the SPIRIT 2025 recommendations.

**Fig 1:**
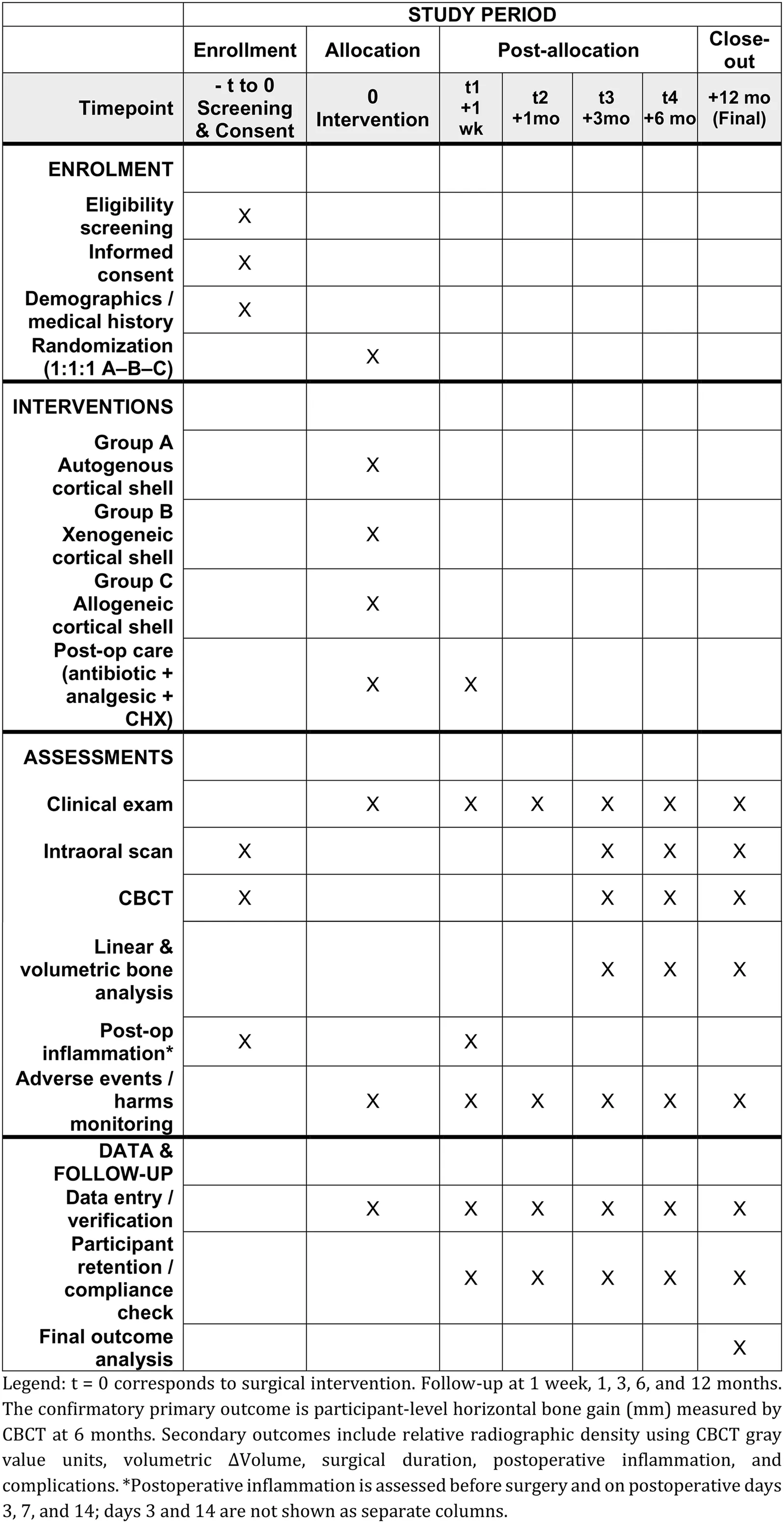
SPIRIT 2025 schedule of enrolment, interventions, and assessments.

This study was approved by the Comité de Ética de Investigación en Seres Humanos del Instituto Superior Tecnológico Portoviejo (CEISH-ITSUP), Ecuador (Approval No. 1715824376). All procedures will comply with the Declaration of Helsinki and national regulations. Written informed consent will be obtained from all participants prior to any study procedure. The ethics committee will receive semiannual progress reports; any protocol amendments will be submitted for review and approval before implementation. Adverse events will be recorded and reported according to institutional policy.

### Participants

Eighty-seven adults (18–64 years) requiring horizontal ridge augmentation in the posterior mandible (Cawood & Howell IV–VI) will be enrolled. Participants may present one or two eligible posterior mandibular edentulous sites. When two sites are eligible, both will be treated under the participant’s allocated intervention to preserve the parallel-group design. All participants must present good oral hygiene (plaque index < 20%) and systemic health suitable for surgery.

### Exclusion criteria

- Systemic conditions or medications affecting bone healing.
- Current heavy smoking (>5 cigarettes/day).
- Pregnancy or lactation.
- Chemotherapy or radiotherapy to the head and neck.
- Active periodontal disease or infection.

Smoking status will be assessed at baseline by structured self-report and categorized as current heavy smokers (>5 cigarettes/day), light smokers (≤5 cigarettes/day), former smokers (cessation ≥6 months), or never smokers. Only current heavy smokers will be excluded. Smoking history and vaping use will be recorded for descriptive and exploratory analyses.

Prior to enrollment, all participants will receive both verbal and written explanations of the study objectives, procedures, potential risks, and benefits. Written informed consent will be obtained using a printed physical document signed by hand by each participant before any study-related activity. All signed consent forms will be securely stored in the physical records of the UEES Dental School, accessible only to authorized study personnel.

### Study groups and interventions

Each participant will be assigned to one treatment group only (autogenous, xenogeneic, or allogeneic cortical shell), following computer-generated block randomization in a 1:1:1 ratio.

#### Group A – Autogenous cortical shell.

Cortical plates (~1 mm) harvested from the mandibular ramus using a piezoelectric device (*Piezosurgery touch, Mectron, Italy*) will be adapted and fixed with LevelOne® 1.0 mm titanium micro screws (*KLS Martin Group, Tuttlingen, Germany*) made of a Ti-6Al-4V implant-grade alloy (ASTM F136/ DIN ISO 5832−3). These self-retaining Centre-Drive screws are designed for micro-osteosynthesis in oral and maxillofacial surgery, providing excellent torque control and minimal soft-tissue irritation. The enclosed space is filled with a 1:1 mixture of autogenous bone chips and xenogeneic particulate (*Bio-Oss, Geistlich Pharma AG, Switzerland*).

#### Group B – Xenogeneic cortical shell.

Pre-fabricated bovine laminae (*OsteoBiol® Lamina, Tecnoss®, Italy*) will be rehydrated for five minutes, trimmed to contour, and fixed with screws of the same specifications. The space is filled with the same bone mixture.

#### Group C – Allogeneic cortical shell.

Human-derived cortical sheets (*Maxgraft® Cortical Sheet, Botiss®, Germany*) will be rehydrated for ten minutes and shaped before fixation with screws. The internal space is filled identically to Groups A and B.

### Surgical procedure

All surgical procedures will be carried out under local anesthesia using Articaine 4% with epinephrine 1:100,000 (*Septodont®, France*). Each intervention will be performed under aseptic conditions with full-thickness flap elevation to expose the posterior mandibular edentulous ridge.

A mid-crestal incision will be made along the alveolar crest of the edentulous ridge, followed by two small vertical releasing incisions positioned at the mesial and distal limits of the surgical field to ensure adequate access and flap mobility. The flap will be carefully reflected to expose the bone, avoiding damage to the periosteum at the base of the incision.

The recipient site will be decorticated using a round carbide bur under copious sterile saline irrigation to promote bleeding and facilitate graft revascularization. Small perforations will be made through the cortical plate until medullary bleeding is observed, following the principles described by Khoury and Hanser (2015).

Once the graft corresponding to each experimental group has been positioned and stabilized, and the grafting space filled, a tension-free closure will be performed. The flaps will be passively repositioned after periosteal releasing incisions, ensuring complete coverage of the grafted area and proper adaptation over the augmented ridge.

Wound closure will be achieved using 6−0 blue monofilament nylon sutures (*TechSuture®, São Paulo, Brazil*), a non-absorbable material that provides high tensile strength, precise wound approximation, and minimal tissue reactivity. Interrupted and mattress sutures will be used according to flap configuration to achieve stable primary closure.

Postoperative management will include:

- Antibiotic therapy: *Amoxicillin 875 mg + Clavulanic acid 125 mg*, orally every 12 hours for 7 days.
- Analgesic regimen: *Ibuprofen 600 mg* every 8 hours for 3 days.
- Antiseptic rinse: *0.12% chlorhexidine gluconate* twice daily for two weeks.

Patients will be instructed to maintain a soft diet and avoid mechanical trauma in the grafted area. Sutures will be removed 14 days after surgery.

Follow-up visits will be scheduled at 1 week, 1 month, 3 months, 6 months, and 12 months postoperatively. At each follow-up, clinical evaluation of wound healing, tissue integrity, and possible complications (e.g., dehiscence, infection, graft exposure) will be documented.

Measured outcomes will include bone gain (mm^3^), relative radiographic density (gray value units), surgical duration (minutes), and postoperative facial swelling (mm). Postoperative facial swelling will be assessed as a standardized extraoral linear measurement from the ipsilateral oral commissure to the tragus using a calibrated flexible millimeter tape. Participants will be seated upright with the Frankfort horizontal plane parallel to the floor during measurement. Baseline measurements will be recorded preoperatively to allow calculation of postoperative change (Δ swelling). At postoperative days 3, 7, and 14, measurements will be repeated, and a standardized photographic record of the measurement region will be obtained under consistent head positioning and lighting conditions to ensure accurate landmark identification and minimize inter-examiner variability. Each measurement will be performed twice by the same calibrated examiner, and the mean value will be recorded. This variable will be interpreted as a secondary exploratory proxy measure of facial edema.

### Volumetric and morphometric analysis

Quantitative three-dimensional evaluation will be conducted through a standardized digital workflow that integrates intraoral surface scanning and CBCT-based volumetric analysis. This multimodal approach ensures reproducible evaluation of graft remodeling and contour stability over time.

1. **Intraoral scanning:**

Each participant will undergo digital impression acquisition using a TRIOS® 3 Wired intraoral scanner (*3Shape A/S, Copenhagen, Denmark*) before surgery and at each follow-up interval (3, 6, and 12 months). The scanner provides high-resolution color models (accuracy < 20 μm) that enable precise surface documentation. The resulting STL files will serve to evaluate external volumetric changes and soft-tissue adaptation over time.

2. **CBCT-based segmentation and linear measurements:**

Preoperative and postoperative cone-beam computed tomography (CBCT) scans will be obtained using the Planmeca Viso® G7 CBCT system (*Planmeca Oy, Helsinki, Finland*) under standardized exposure parameters and patient positioning. DICOM datasets will be segmented in the Romexis® 3D module to isolate the grafted region of interest. Surface meshes of the reconstructed bone will be exported as STL files (1:1 scale) for morphometric analysis. Baseline ridge width will be measured on the preoperative CBCT using the same standardized reference system applied for postoperative assessments. A perpendicular reference line relative to the alveolar ridge inclination will be established, and horizontal measurements will be obtained at 5, 7, and 11 mm apical to the alveolar crest. The mean baseline ridge width derived from these levels will be used as the covariate in the primary ANCOVA model to adjust for initial ridge dimension differences among groups.

In addition to volumetric evaluation, linear bone measurements will be performed directly in Romexis® following the methodologies described by Monje et al. (2015) and Amaral Valladão et al. (2020).

- For horizontal gain, a perpendicular reference line will be traced relative to the alveolar ridge inclination, and three horizontal lines will be drawn at 5, 7, and 11 mm from the alveolar crest to measure ridge width at each level.
- For vertical gain, measurements will extend from the most coronal point of the alveolar crest to the upper border of the mandibular canal or basal cortical plate, depending on the anatomic limits of each case.
- Each measurement will be performed by two calibrated examiners following standardized protocols and repeated twice with a two-week interval to evaluate intra- and inter-examiner reliability using intraclass correlation coefficients (ICC). An ICC of 0.90 or higher will be considered the prespecified reliability criterion.
- The resulting data will be expressed as the mean horizontal and vertical bone gain (mm) and compared among the experimental groups.

This standardized approach, based on Monje et al. (2015) and Amaral Valladão et al. (2020), allows reproducible quantification of linear bone gain and complements the three-dimensional volumetric workflow performed with Romexis and CloudCompare, ensuring geometric and clinical accuracy in mandibular bone-augmentation assessment.

Radiographic density will be assessed using device-specific gray value measurements obtained from the CBCT datasets. It is acknowledged that CBCT-derived gray values do not represent calibrated Hounsfield Units (HU) as in medical CT. Therefore, density values will be interpreted as relative within-device measurements under standardized acquisition parameters (same machine, field of view, voxel size, and exposure settings for all scans).

3. **Surface registration and quantitative analysis:**

STL models obtained from both the TRIOS 3 intraoral scans and Romexis segmentations will be imported into CloudCompare (*EDF R&D, Paris, France*) for alignment and measurement. Surface registration will be performed using the *Iterative Closest Point (ICP)* algorithm (overlap 0.8, max iterations 100, sampling 50 000, RMS < 0.2 mm). Color-coded *Cloud-to-Mesh* distance maps (0–2 mm) will visualize local contour differences and bone volume changes between time points. This enables assessment of both hard-tissue (bone) and soft-tissue remodeling in the grafted regions.

Volume computation will be performed using the *Compute Mesh Volume* function, and the volumetric change (ΔVolume = Vol_post – Vol_pre) will be recorded for each site. All analyses will be repeated twice to ensure measurement reliability, with a mean registration error below 0.1 mm. Quantitative data will be exported for statistical comparison.

This integrated workflow, combining intraoral scanning, CBCT and surface-based morphometric evaluation, provides a transparent, reproducible, and fully digital protocol for monitoring bone and soft-tissue remodeling following cortical shell augmentation.

4. **Morphology** **analysis**

Surface-based morphometry will be performed by rigidly aligning pre- and postoperative CBCT-derived meshes on anatomically stable mandibular regions (lingual cortical base and basal mandibular contours) to avoid bias from the augmented site. After alignment, point-to-surface distance maps will be computed to generate color-coded deviation maps (positive values = apposition; negative values = resorption). Morphological metrics will include mean absolute distance (MAD), root-mean-square (RMS) distance, 95th-percentile distance, and the signed volume of positive/negative deviations within a standardized region of interest (ROI) that envelopes the augmented ridge. Mesh preprocessing (isotropic remeshing, light Laplacian smoothing) and ICP registration parameters will be pre-specified. Two calibrated examiners will repeat the full pipeline twice (two-week interval); intra-/inter-rater reliability will be reported (ICC).

This surface-based approach complements linear and volumetric outcomes and localizes remodeling patterns across the augmented contour, following validated CBCT subtraction and surface-registration workflows for alveolar augmentation [10,11].

If shape-based correspondence analysis is performed, SPHARM-PDM algorithms may be applied to assess 3D curvature and topographic variations, as described by Kim et al. [12].

### Randomization and blinding

Participants will be randomly allocated to one of the three treatment groups using a computer-generated randomization sequence created through the web-based platform Random.org (Randomness and Integrity Services Ltd., Dublin, Ireland), which generates true random numbers based on atmospheric noise. Block randomization with variable block sizes will be used to ensure balanced group allocation while maintaining unpredictability.

The allocation sequence will be generated by an independent researcher not involved in participant recruitment, surgical procedures, or outcome assessment. Allocation concealment will be ensured using sequentially numbered, opaque, sealed envelopes (SNOSE), prepared in advance and stored securely. Envelopes will be opened only after confirmation of eligibility and written informed consent, immediately prior to surgery.

The statistician responsible for the primary analysis will remain blinded to treatment allocation through coded group identifiers until completion of the primary analysis. Due to the surgical nature of the interventions and the requirement for autogenous bone harvesting in one group, blinding of the operating surgeon and participants is not feasible. Outcome measurements (CBCT segmentation and digital morphometric analyses) will be performed according to standardized protocols; however, these procedures will not involve masked allocation, and group identity will not be concealed from the operators performing image processing. CBCT datasets and intraoral scan files will be coded before measurement to remove participant identifiers, and duplicate measurements will be performed independently by two calibrated examiners. If the prespecified reliability criterion is not achieved, examiners will repeat calibration before final measurements are completed.

If a participant presents two eligible posterior mandibular sites, both sites will receive the same allocated intervention; no within-participant randomization will be performed.

### Sample size

The sample size was determined a priori using G*Power version 3.1.9.7 (Heinrich-Heine-Universität Düsseldorf, Germany). The calculation was based on the two prespecified confirmatory comparisons of the autogenous cortical shell group (Group A) versus the xenogeneic shell group (Group B) and versus the allogeneic shell group (Group C). For sample-size planning, a conservative two-sided α of 0.025 per comparison was used to account for multiplicity across the two primary contrasts; the final confirmatory analyses will control the family-wise type I error using the Holm procedure.

A clinically relevant between-group difference of 1.0 mm in horizontal bone gain at 6 months and a common standard deviation of approximately 1.12 mm were assumed, corresponding to a standardized effect size of Cohen’s d = 0.893. The standard deviation was derived from a previous CBCT-based randomized clinical trial reporting 6-month variability in horizontal ridge augmentation, with standard deviations of 1.35 mm and 0.87 mm across two treatment arms and an approximate pooled standard deviation of 1.12 mm [13]. Under these assumptions, with equal allocation and 80% power, G*Power indicated that 26 evaluable participants per group are required (52 participants for each pairwise comparison; achieved power approximately 81.5%).

Because the trial includes three parallel groups, 78 evaluable participants are therefore required. To account for an anticipated attrition rate of approximately 10%, the enrollment target was increased to 29 participants per group, resulting in a total planned sample of 87 participants.

The power calculation was performed independently of any anticipated efficiency gain from baseline adjustment in the primary ANCOVA model; therefore, no baseline-to-follow-up correlation or variance reduction was assumed. The stated power refers to each prespecified confirmatory contrast evaluated at the conservative two-sided α of 0.025 used for planning.

Because the variance estimate was obtained from a previous randomized trial conducted under a related but not identical anatomical and clinical setting, sensitivity analyses were additionally considered across plausible standard deviations. With a 1.0-mm target difference and two-sided α = 0.025, approximately 26, 29, 36, and 39 evaluable participants per group would be required to achieve 80% power for assumed standard deviations of 1.12, 1.20, 1.35, and 1.40 mm, respectively. These estimates illustrate the sensitivity of statistical power to the transportability of the variance assumption. Full details of the calculation and sensitivity analysis are provided in Supplementary File 1.

### Statistical analysis

#### Primary and secondary endpoints.

The single primary endpoint of this trial is horizontal bone gain (mm) at 6 months, analyzed at the participant level. The primary comparison will assess adjusted between-group differences in horizontal bone gain at 6 months. This trial follows a superiority framework. The primary objective is to determine whether the autogenous cortical shell (Group A) provides greater horizontal bone gain at 6 months than each prefabricated alternative. Accordingly, two primary, pre-specified pairwise comparisons will be tested: Group A vs Group B (xenogeneic shell) and Group A vs Group C (allogeneic shell). The comparison between Groups B and C will be considered exploratory.

For each primary comparison (A vs B and A vs C), the null hypothesis is that the adjusted mean horizontal bone gain at 6 months is equal between groups, and the alternative hypothesis is that Group A shows greater adjusted mean gain than the comparator group.

In this trial, the primary endpoint is analyzed at the participant level by using a participant-level summary (mean value when two sites are treated). Because no within-participant randomization is performed and both sites receive the same allocated intervention, clustering at the site level does not affect the primary analysis. Therefore, no additional random-effects structure is required for the primary endpoint model.

Secondary endpoints include horizontal bone gain at 3 and 12 months, volumetric change (ΔVolume), morphometric parameters (MAD, RMS, 95th percentile distance), relative radiographic density values (gray units), surgical duration, postoperative facial swelling, and soft tissue contour changes derived from intraoral surface scans.

Longitudinal analyses across time points (3, 6, and 12 months) will be considered secondary and exploratory.

Soft tissue contour changes will be analyzed as exploratory secondary outcomes based on surface deviation metrics obtained after STL alignment. These analyses aim to describe external volumetric and morphological remodeling but will not be considered confirmatory endpoints.

All statistical analyses will be conducted using IBM SPSS Statistics v28.0 (IBM Corp., USA). Statistical model assumptions will be evaluated primarily through residual diagnostics, including assessment of residual normality, homoscedasticity, linearity, and influential observations, rather than relying solely on normality testing of the raw outcome data. To control the family-wise type I error across the two primary pairwise comparisons (A vs B and A vs C), multiplicity adjustment will be applied using the Holm step-down procedure (family-wise α = 0.05). Adjusted p-values will be reported. In addition, adjusted mean differences with 95% confidence intervals will be presented for each primary comparison.

The primary analysis will evaluate between-group differences in horizontal bone gain (mm) at 6 months using an ANCOVA model with treatment group as a fixed factor and baseline ridge width as a covariate. The two primary contrasts of interest are Group A vs Group B and Group A vs Group C; multiplicity adjustment will be applied as specified above. Model assumptions will be checked, and results will be reported as adjusted mean differences with 95% confidence intervals.

**Secondary outcome analyses.** Secondary outcomes include horizontal bone gain at 3 and 12 months, vertical bone gain, volumetric change (ΔVolume), morphometric parameters (MAD, RMS, and 95th-percentile distance), relative CBCT gray values, surgical duration, postoperative complications, repeated postoperative facial-swelling measurements, and soft-tissue contour changes. Horizontal bone gain at 12 months, vertical bone gain, and postoperative complications will be considered key secondary outcomes; all additional morphometric, volumetric, radiographic-density, and surface-based outcomes will be considered exploratory. No formal multiplicity adjustment will be applied to secondary endpoints, and these findings will be interpreted as supportive or exploratory rather than confirmatory. Effect estimates and 95% confidence intervals will be emphasized.

**Longitudinal horizontal bone gain.** Horizontal bone gain across 3, 6, and 12 months will be analyzed at the participant level using participant-level summaries at each time point (mean value when two sites are treated and the single-site value otherwise). A linear mixed-effects model will include Group, Time, and Group × Time interaction as fixed effects, baseline ridge width as a covariate, and Participant as a random intercept. Time will be treated as a categorical factor. An unstructured covariance matrix will be used to model within-participant correlations across repeated measurements. The Group × Time interaction will assess whether changes over time differ among treatment groups. Estimated marginal means, between-group contrasts at each time point, and corresponding 95% confidence intervals will be reported. Model assumptions will be assessed using residual diagnostics, including residual normality, homoscedasticity, and influential observations.

All available repeated observations will be included in the mixed-effects models. Under the missing-at-random assumption, likelihood-based estimation will be used without requiring complete observations at all follow-up visits. No last-observation-carried-forward or single-value imputation will be used for the longitudinal mixed-effects analyses.

As a supportive analysis, horizontal bone gain may also be analyzed at the site level using all available site-specific measurements. In this model, Site will be nested within Participant, with random intercepts for Participant and Site-within-Participant, and site-specific baseline ridge width will be included as a covariate. These analyses will account for within-participant clustering when two treated sites contribute data and will not replace the participant-level analysis.

**Volumetric change (ΔVolume).** Volumetric change will be analyzed at the site level. For repeated measurements, linear mixed-effects models will include Group, Time, and Group × Time interaction as fixed effects, with Participant and Site-within-Participant included as random effects. For a single follow-up assessment, Group will be included as a fixed effect and Participant as a random intercept. Treatment effects will be expressed as adjusted mean differences with 95% confidence intervals.

**Morphometric parameters.** Mean absolute distance (MAD), root mean square (RMS) deviation, and 95th-percentile distance obtained from surface-based analyses will be analyzed at the site level. Cross-sectional comparisons will use linear mixed-effects models with Group as a fixed effect and Participant as a random intercept. When measurements are repeated over time, Group, Time, and Group × Time interaction will be included as fixed effects, with random intercepts for Participant and Site nested within Participant. Adjusted mean differences and 95% confidence intervals will be reported.

**Relative CBCT gray values.** Relative radiographic-density measurements will be analyzed at the site level using linear mixed-effects models. For repeated measurements, models will include Group, Time, and Group × Time interaction as fixed effects and random intercepts for Participant and Site-within-Participant. Results will be expressed as adjusted mean differences with 95% confidence intervals. These measurements will be interpreted as device-specific relative gray values rather than absolute Hounsfield units.

**Surgical duration.** Surgical duration will be recorded from the administration of local anesthesia to the final suture for each surgical session. Surgical duration will be analyzed at the participant level using a linear regression model with treatment group as the primary predictor. If clinically relevant prespecified covariates are included, adjusted mean differences and 95% confidence intervals will be reported. If two sites are treated during the same surgical session, total surgical duration for that session will constitute the participant-level observation.

**Postoperative facial swelling.** Repeated facial-swelling measurements will be analyzed at the participant level using a linear mixed-effects model with Group, Time, and Group × Time interaction as fixed effects and Participant as a random intercept. Time will be treated as categorical. The Group × Time interaction will evaluate differences in postoperative swelling trajectories among treatments. Estimated marginal means, between-group differences at prespecified postoperative time points, and 95% confidence intervals will be reported.

**Soft-tissue contour changes.** Soft-tissue contour changes derived from aligned intraoral surface scans will be analyzed at the site level. Cross-sectional comparisons will use linear mixed-effects models with Group as a fixed effect and Participant as a random intercept. For repeated assessments, Group, Time, and Group × Time interaction will be included as fixed effects, with random intercepts for Participant and Site-within-Participant. Treatment effects will be reported as adjusted mean differences with 95% confidence intervals.

**Postoperative complications.** Binary postoperative complications will be analyzed using generalized linear mixed-effects models with a logit link. Group will be included as a fixed effect and Participant as a random intercept when site-level complications are recorded and a participant contributes two sites. Treatment effects will be reported as odds ratios with 95% confidence intervals. For rare complications or sparse data, Fisher’s exact test will additionally be used for descriptive between-group comparisons.

All secondary longitudinal mixed-effects analyses will use all available observations and will not require complete follow-up at every time point. Under the missing-at-random assumption, likelihood-based mixed-effects estimation will be used without last-observation-carried-forward or single-value imputation. Model adequacy will be evaluated using residual diagnostics, including residual distribution, homoscedasticity, linearity, and influential observations. Appropriate transformations or robust alternatives will be considered if model assumptions are materially violated.

**Missing data.** Every effort will be made to minimize missing outcome data through scheduled follow-up reminders and predefined imaging windows. The amount, pattern, and reasons for missing data will be summarized by treatment group. Baseline characteristics of participants with and without available 6-month primary-outcome data will also be described to assess potential evidence of differential attrition.

The primary analysis will follow the intention-to-treat principle and will include all randomized participants according to their assigned treatment group. If any primary-outcome data at 6 months are missing, multiple imputation by chained equations (MICE) will be used under a missing-at-random (MAR) assumption. Twenty imputed datasets will be generated. The imputation model will include treatment group, baseline ridge width, age, sex, smoking status, available horizontal ridge-width measurements at other follow-up time points, and clinically relevant variables associated with outcome or loss to follow-up, including postoperative complications when available. The prespecified ANCOVA model will be fitted separately to each imputed dataset, and treatment-effect estimates and standard errors will be combined using Rubin’s rules [14].

A complete-case ANCOVA restricted to participants with observed 6-month primary-outcome data will be performed as a sensitivity analysis and will not replace the prespecified multiple-imputation analysis.

To assess departures from the MAR assumption, a pattern-mixture delta-adjustment sensitivity analysis will be performed. Because the primary superiority hypothesis favors Group A (autogenous cortical shell), the MNAR sensitivity analysis will impose assumptions unfavorable to Group A. Imputed 6-month horizontal bone-gain values for participants with missing outcomes in Group A will be progressively reduced relative to their MAR-based imputations, while imputed values in Groups B and C will initially remain unchanged. Prespecified delta reductions of −0.25, −0.50, −0.75, and −1.00 mm will be evaluated.

A tipping-point analysis will additionally increase the unfavorable delta adjustment in 0.10-mm increments until the statistical conclusion for each primary contrast (A vs B and A vs C) changes. The tipping point will therefore indicate the magnitude of an unobserved unfavorable departure from MAR that would be required to overturn the primary superiority conclusion.

For secondary longitudinal outcomes analyzed using linear mixed-effects models, all available repeated observations will be included. These models provide valid likelihood-based estimates under the MAR assumption and do not require complete observations at every follow-up visit. No single-value imputation or last-observation-carried-forward procedure will be used. The number of available observations at each time point and patterns of missing follow-up measurements will be reported by treatment group.

### Data management and confidentiality

Clinical and radiographic information will be entered into encrypted electronic case report forms (eCRFs) with double data entry to minimize transcription errors. Each participant will be assigned a unique study code; identifying data will be stored separately from clinical outcomes. De-identified datasets and STL files will be kept on a secure UEES institutional server with access restricted to authorized study staff. After publication, the de-identified participant-level dataset underlying the reported results, the data dictionary, metadata, and statistical code will be deposited in a public repository in accordance with PLOS ONE data policies. Complete CBCT/DICOM and intraoral-scan imaging files will not be publicly released because they may contain potentially identifiable anatomical information. Access to these imaging files, when scientifically justified and ethically permissible, will be evaluated through an institutional data-access process and will not depend solely on the discretion of the corresponding author.

### Expected results and discussion

This trial is expected to identify whether horizontal bone gain, dimensional stability, surgical duration, and postoperative morbidity differ among autogenous, xenogeneic, and allogeneic cortical shells under standardized surgical and digital assessment conditions. Autogenous cortical shells may show greater early bone gain given their osteogenic potential, while prefabricated xenogeneic and allogeneic laminae may differ in operative time, donor-site morbidity, and volumetric stability.

The integrated digital workflow is designed to deliver objective, reproducible quantification of remodeling. By combining linear metrics [15,16] with volumetric ΔVolume and colorimetric deviation maps, the study can detect subtle differences across materials beyond conventional 2D measurements.

Limitations include the single-center setting and 12-month follow-up, which may not capture long-term remodeling beyond one year. The parallel-group design ensures independence between interventions and allows direct statistical comparison among three distinct grafting materials under standardized surgical and analytical conditions.

## Supporting information

S1 File Sample size calculation. (DOCX)

S2 File SPIRIT 2025 checklist. (DOCX)

S3 File Original study protocol. (PDF)
