# Supplementary material for: Comparison of bone augmentation techniques in the mandibular posterior region: A randomized clinical trial protocol
Source: PLoS One. 2026 Oct 29;21(10):e0337304. doi: 10.1371/journal.pone.0337304 (PMC13718928; doi:10.1371/journal.pone.0337304)
Supplement: Supplementary file 1 [file pone.0337304.s003.pdf]

Original Study Protocol

Annex 12

Clinical behavior in the regeneration of soft and bone tissues of the oral cavity in patients with missing teeth and dental defects at the Dentistry Clinic of Universidad Espíritu Santo from 2024 to 2029

Form for submitting protocols for observational and interventional research involving human participants (except clinical trials).

Instructions

This form must be completed in full in Spanish, using 10-point Times New Roman, single spacing, A4 paper, and 2.5 cm margins on all sides. Additional table rows may be created if needed, while observing the text limits in individual sections. The form must not exceed 20 pages. Arguments must be supported by current bibliographic references and statistical data, cited in the text using a number of [sentence incomplete in the source].

General research information

Research type

Mark the applicable option with an X.

Observational studies	Interventional studies
Cross-sectional studies	Quasi-experimental studies
Ecological studies	Field trial
Case reports	X – Randomized controlled trials without medicines and/or mobile devices
Case series	
Case-control studies	
Cohort studies	
Other — specify:	

Research implementation period

60 months
1 May 2024 to 30 April 2029

Research funding

Field	Information
Total research funding	122,410.80
Funding sources	Research Center – Universidad de Especialidades Espíritu Santo

Sponsor information

The sponsor is the natural or legal person, company, institution, enterprise, or organization, including academic organizations, legally constituted and legally represented in the country, responsible for initiating, managing, developing, and funding the research.

Name of the person or institution conducting the research

Field	Information
Sponsor	Universidad Espíritu Santo
National identity number / tax identification number (RUC)	
Institutional telephone	(593) - 045000950
Extension	
Email	
Address	km 2.5, road to Samborondón
Institutional website	www.uees.edu.ec
Implementing body	Research Center – Universidad de Especialidades Espíritu Santo

Geographical coverage

Select only one type of coverage.

Coverage	Area or specification
National	
Planning zone 1	Carchi, Esmeraldas, Imbabura, Sucumbíos
Planning zone 2	Napo, Orellana, Pichincha
Planning zone 3	Chimborazo, Cotopaxi, Pastaza, Tungurahua
Planning zone 4	Manabí, Santo Domingo de los Tsáchilas
Planning zone 5	Bolívar, Guayas, Los Ríos, Santa Elena
Planning zone 6	Azuay, Cañar, Morona Santiago
Planning zone 7	El Oro, Loja, Zamora Chinchipe
Planning zone 8	Guayaquil, Samborondón, Durán cantons
Planning zone 9	Metropolitan District of Quito
Provincial	Specify the provinces where the research will take place.
Local	Specify the province and cantons where the research will take place.

Research personnel

Note: Include all investigators. Add a row for each member of the scientific and technical team if necessary. If the sponsor is also an investigator, repeat the name in both roles.

Principal investigator	Information
Full name	Antonio Gabriel Lanata Flores
National identity number / passport	0916524820
Academic qualification	Dentist
Institution	UEES
Personal and institutional email	alanataf@uees.edu.ec
Mobile telephone	0983820084

Additional role	Name / identification / qualification / institution / email / telephone
Sponsor	
Investigator 1	
Investigator 2	
Technician / Assistant	

Research details

Structured abstract

The abstract must contain the following sections (maximum one page):
1. Title
2. Introduction
3. Objectives
4. Methods
5. Expected results

Research problem

Patients with missing teeth commonly experience dysfunction. Functional impairments occur because tooth loss alters occlusion, reduces the stability of the remaining teeth, and is accompanied by ongoing bone remodeling in the edentulous area.

Edentulous areas undergo progressive bone remodeling, which often complicates bone rehabilitation. Dental implant placement is currently the first-choice rehabilitation option for edentulous areas. Patients with extensive bone loss resulting from bone remodeling require complex bone regeneration procedures involving autogenous grafts or biomaterials. The conventional bone regeneration technique was conceptualized by Urban et al.[1] in 2013. It uses a 1:1 mixture of autogenous bone particles harvested from the patient's mandible and anorganic bovine bone, covered with a resorbable collagen membrane secured to the patient's bone with tacks.

The horizontal bone regeneration technique using autogenous bone described by Khoury et al.[2] involves two bone blocks stabilized with miniscrews, with the defect filled with autogenous bone particles. Autogenous bone is harvested from areas such as the mandibular ramus, mandibular angle (retromolar region), or chin symphysis. In the original Khoury technique, bone is harvested from the donor site using a micromotor, saw, or piezoelectric device. The 2021 systematic review by Sánchez et al.[3] states: "The availability of RCTs comparing these techniques specifically is scarce, so it will be appropriate to design comparative studies in future in order to understand implant behavior and marginal bone loss with a longer follow-up".

During healing after extraction, the alveolar bone undergoes additional morphological changes as a result of natural remodeling. This begins immediately after extraction, with the greatest resorption occurring during the first two months, although dimensional changes can still be observed one year after surgery. Various studies have reported greater resorption of the buccal wall than of the lingual socket wall. Regarding donor-site soft tissues, there is only one randomized controlled clinical trial report validating the use of flowable resin with collagen hemostatic sponges, and there are no studies on self-adhesive dressings for palatal protection. The intention is to evaluate their use as alternative techniques to protect the palate after free gingival graft harvesting and to assess healing and postoperative pain.

PROBLEM

There is no consensus on the benefits and indications of soft tissue and bone regeneration techniques.

Bone regeneration studies are limited by inadequate follow-up.

Urban, I.A.; Lozada, J.L.; Jovanovic, S.A.; Nagy, K. Horizontal guided bone regeneration in the posterior maxilla using recombinant human platelet-derived growth factor: A case report. Int. J. Periodontics Restor. Dent. 2013, 33, 421–425.

Khoury, F.; Hanser, T. Mandibular bone block harvesting from the retromolar region: A 10-year prospective clinical study. Int. J. Oral Maxillofac. Implants 2015, 30, 688–697.

Sánchez-Sánchez,J.; Pickert, F.N.; Sánchez-Labrador, L.; GF Tresguerres, F.; Martínez- González, J.M.; Meniz-García, C. Horizontal Ridge Augmentation: A Comparison between Khoury and Urban Technique. Biology 2021, 10, 749.

Horváth A, Mardas N, Mezzomo LA, Needleman IG, Donos N. Alveolar ridge preservation. A systematic review. Clin Oral Investig [Internet]. 2013 Mar 1 [cited 2023 Aug 5];17(2):341–63.

Mauricio G Araújo 1, Jan Lindhe, Dimensional ridge alterations following tooth extraction. An experimental study in the dog. Journal of Clinical Periodontology, 32(2), 212–218

Oreste Iocca, Alessio Farcomeni, Simon Pardiñas Lopez, Huzefa S. Talib. Alveolar ridge preservation after tooth extraction: a Bayesian Network meta-analysis of grafting materials efficacy on prevention of bone height and width reduction. Journal of Clinical Periodontology, 44(1), 104–114 | 10.1111/jcpe.12633.

Meza-Mauricio J, Mourão ERST, Oliveira Marinho K, Vergara-Buenaventura A, Mendoza-Azpur G, Muniz FWMG, et al. Effect of collagen sponge and flowable resin composite on pain management after free gingival graft harvesting: A randomized controlled clinical trial. Eur J Oral Sci. 2023;131:e12935. https://doi.org/10.1111/eos.12935Cairo F, Pagliaro U, Nieri M. Soft tissue management at implant sites. En: Journal of Clinical Periodontology. 2008. p. 163-7.

Rationale

Bone regeneration using bone plates and microscrews is a highly precise but low-cost procedure. Developing a feasible surgical protocol for dental surgeons specializing in implantology is therefore of considerable interest. Bioactive glass has also been shown to be an alternative for bone tissue repair and replacement. Most reductions in bone volume occur at two and six months after tooth extraction, as reported in the article by Araújo and Lindhe. During alveolar ridge preservation, the surgeon must remove all granulation tissue remaining in the socket; inadequate removal may compromise the effectiveness of ridge preservation. Conversely, research has demonstrated advantages of bioactive glass arising from its antimicrobial components. There is currently little or no scientific evidence concerning this biomaterial when grafted into post-extraction sockets.

Soft tissue grafting causes substantial pain complaints among patients. The aim is therefore to reduce postoperative morbidity and surgical time and improve soft tissue healing after harvesting a free gingival graft and placing it in areas requiring coverage.

Theoretical framework

Soft tissues

Gingival recession (GR) can be defined as exposure of the root surface following apical migration of the gingival margin relative to the cementoenamel junction. (1) GR affects both younger and older populations. Various surveys have found that 88% of individuals aged ≥65 years and 50% of those aged 18–64 years have ≥1 site with gingival recession. (1-20) GR may arise from anatomical or pathological factors or trauma and results in attachment loss. (2)

Root surface exposure caused by GR is frequently associated with dentin hypersensitivity, root caries, non-carious cervical lesions (NCCLs), poor plaque control, and an unaesthetic appearance. (7)

Indications for root coverage procedures include patients' requests for improved aesthetics and reduced root hypersensitivity, as well as an interest in increasing the amount of keratinized tissue. (3)

The presence of keratinized gingiva has long been considered necessary to maintain gingival health. Although the exact amount of keratinized tissue (KT) required for periodontal health remains uncertain, clinicians agree that ≥1 mm of gingiva is needed to maintain periodontal health in patients with optimal plaque control. (24)

Lang and Loe proposed that ≥2 mm of KT is necessary around teeth to maintain periodontal health in patients with average oral hygiene. The current consensus is that ≥2 mm of KT and ≥1 mm of attached gingiva are required around teeth. (25)

Gingival recession is one of the most common aesthetic concerns involving periodontal tissues. Classifying this condition is important for diagnosis, prognosis, and appropriate treatment planning.

In periodontal plastic surgery, correct diagnosis of gingival recession defects (GRDs) is crucial for determining the need for therapy, choosing appropriate treatment approaches, and longitudinally monitoring or predicting changes at treated versus untreated sites. (16)

Numerous diagnostic classifications of GRDs have been proposed over the years. Among these, the Miller classification has been the most prominent in clinical practice for decades. However, this system included a limited set of variables. (17-18)

A new classification was proposed in 2018 by Cairo et al., based on the interdental clinical attachment level. (19)

The Cairo classification is treatment-oriented and predicts root coverage potential by assessing interdental clinical attachment level (CAL). In Cairo RT1 (Miller Classes I and II), 100% root coverage can be predicted. In Cairo RT2 (Miller Class III), some randomized clinical trials indicate the limit of interdental CAL loss within which 100% root coverage is predictable using different root coverage procedures. In Cairo RT3 (Miller Class IV), complete root coverage cannot be achieved. (21-22)

This classification overcomes some limitations of the Miller classification, such as difficulty distinguishing Classes I and II and the use of "soft tissue or bone loss" as an interdental reference to diagnose periodontal destruction in the interdental area. Furthermore, the Miller classification was proposed when root coverage techniques were just emerging, and its root coverage prognoses for the four classes no longer correspond to treatment outcomes achieved with more advanced surgical techniques. (21)

Clinicians should focus on susceptibility factors and modifiable conditions associated with gingival recession. The main susceptibility factors are thin gingival tissue, absence of KT or a narrow band (<2 mm), probing depths extending beyond the mucogingival junction (MGJ), and a positive history of gingival recession and/or inflammatory periodontal disease. Modifiable conditions include plaque accumulation, inflammatory periodontal disease, aberrant frenum position, traumatic oral hygiene habits, subgingival restoration margins, smoking, and systemic conditions such as diabetes. Clinicians should identify these factors and, where possible, modify them accordingly. (23)

Numerous periodontal plastic surgery techniques have been suggested for treating GR. Connective tissue grafting (CTG) with a coronally advanced flap (CAF) produces gains in keratinized tissue and predictable treatment outcomes. (2) Another widely used procedure is the tunnel technique, which uses a supraperiosteal flap to improve vascularization of the flap and graft. (4)

Other techniques are available for unfavorable mucogingival conditions, such as marginal frenum attachment or a shallow vestibule; the free gingival graft (FGG) has been proposed for these situations. (5) An FGG is a soft tissue graft harvested from the palate with its overlying epithelium. (8-12) One of its main indications is to restore adequate keratinized tissue and gingival thickness in the presence of mucogingival defects. (8-9)

Considerable variability in subsequent root coverage has been reported with this technique (range: 11%–87%; mean: 72%). One challenge is the limited blood supply because part of the graft is positioned on an avascular recipient site, namely the exposed root surface. (5)

Recently, Carcuac and Derks proposed a modified FGG (ModFGG), in which a connective tissue pedicle flap is turned from its apical origin to cover the exposed root surface before placement of the FGG. (6-10)

The rationale for this approach is to improve graft revascularization. Scientific evidence indicates that complete root coverage (CRC) can be achieved in all patients at three months. However, trials comparing ModFGG with conventional FGG and evaluating the long-term effectiveness of this new technique are lacking. (1-11)

Autologous connective tissue grafting (CTG) is currently considered the gold standard for treating periodontal and peri-implant soft tissue defects. It was initially used to increase edentulous ridge volume and keratinized gingival thickness and to deepen the vestibule. Subsequently, it has been used for numerous procedures, including root coverage, soft tissue augmentation around dental implants and partially edentulous areas in aesthetic zones, papilla reconstruction, and scar correction. Its use has provided a better prognosis and greater long-term stability of periodontal and peri-implant soft tissues.

Selecting a CTG donor site requires an accurate diagnosis to choose the appropriate technique and procedure according to biological characteristics. The palatal mucosa and tuberosity are suitable CTG donor sites; their inherent genetic and tissue factors have beneficial effects on healing and defect correction. The tuberosity has been considered a favorable harvesting site for thicker fibrous grafts because it contains more lamina propria and less submucosal tissue (glandular and adipose). Such grafts are particularly recommended for volume augmentation in peri-implant hard and soft tissue defects. CTGs from palatal mucosa have yielded successful outcomes in treating mucosal deficiencies. The origin and biological composition of tuberosity grafts have been shown to favor hyperplastic responses, potentially compromising aesthetic areas.

Harvesting a CTG from the palate has been described as the preferred option for mucosal defects because visibility and access are better, and the size of the palate allows a graft with greater diameter and length than can be obtained from the tuberosity.

Various techniques can be used to harvest a CTG from the palate, leaving the site either protected by epithelium, as in a subepithelial connective tissue graft (SCTG), or unprotected, as in an FGG. The FGG technique offers advantages in graft design, size, and ease of harvesting, but causes greater postoperative discomfort, including pain, infection risk, and possible excessive bleeding; healing occurs by secondary intention. With SCTG harvesting, patients experience less discomfort because the wound heals by primary intention, but the technique is more complex and may not yield adequate graft thickness.

Accidental injury to a main vascular trunk or its collateral branches is the most common cause of palatal hemorrhage. When wound edges are separated and cannot be approximated, healing and epithelialization occur by secondary intention over a prolonged period, with defect closure taking approximately two to four weeks. Patients report the highest donor-site pain on the first postoperative day, with pain decreasing over two weeks after surgery.

Palatal donor-site healing occurs through fibroblast proliferation, collagen synthesis, angiogenesis, and wound contraction. Revascularization, immunity, and epithelial cell proliferation are also crucial for optimal wound healing. Accelerating healing and reducing prolonged bleeding and palatal wound pain remain challenging. Various materials have been used to reduce patient morbidity, including hemostatic agents (e.g., absorbable synthetic collagen, absorbable gelatin sponge, cyanoacrylate, oxidized regenerated cellulose, and ferric subsulfate), periodontal dressings, self-adhesive dressings, mechanical protection such as Hawley retainers or thermoformed appliances, and, more recently, platelet concentrates.

Periodontal dressings have proved effective in significantly reducing pain, discomfort, and bleeding and enabling patients to carry out routine activities. Nevertheless, friction against the palatal mucosa during function may cause detachment, leading to bleeding associated with pain, delayed healing, and difficulty speaking.

A self-adhesive dressing made of hydrophilic polymers and hydroxyethyl cellulose, without eugenol, adheres to intraoral surfaces to protect wounds and promote improved tissue healing. It absorbs inflammatory exudate generated during the early healing phases and supports growth factor proliferation. These dressings can be stabilized over wounds, and adhesion time depends on the location and condition of the surgical bed. Postoperative benefits include improved control of pain and bleeding and improved healing.

The diversity of adhesive restorative materials now makes it possible to create a fixed protective barrier for the palatal donor site. Adhesive techniques on adjacent teeth produce a superficial resin layer covering the denuded area and promoting healing by maintaining the clot. This favors local hemostasis and reduces patient morbidity (pain, edema, infection, and necrosis).

Carcuac O, Trullenque-Eriksson A, Derks J. Modified-free gingival graft technique for treatment of gingival recession defects at mandibular incisors: A randomized clinical trial. J Periodontol. 2023 Jan 10. doi: 10.1002/JPER.22-0581. Epub ahead of print. PMID: 36627509.

Akcan SK, Ünsal B. Gingival recession treatment with concentrated growth factor membrane: a comparative clinical trial. J Appl Oral Sci. 2020 Mar 27;28:e20190236. doi: 10.1590/1678-7757-2019-0236. PMID: 32236353; PMCID: PMC7105285.

Cairo F, Nieri M, Pagliaro U. Efficacy of periodontal plastic surgery procedures in the treatment of localized facial gingival recessions. A systematic review. J Clin Periodontol. 2014;41(15):S44-62.

Ramos UD, Bastos GF, Costa CA, de Souza SLS, Taba M Jr, Novaes AB Jr. Root coverage with tunneling technique or modified advanced flap associated with acellular dermal matrix: results from 6 months randomized clinical trial. Clin Oral Investig. 2022 Jan;26(1):773-780. doi: 10.1007/s00784-02104055-8. Epub 2021 Aug 7. PMID: 34363104.

Pini Prato G, Clauser C, Cortellini P, Tinti C, Vincenzi G, Pagliaro U. Guided tissue regeneration versus mucogingival surgery in the treatment of human buccal recessions. A 4-year follow-up study. J Periodontol. 1996;67:1216-1223.

Fernández-Jiménez A, García-De-La-Fuente AM, Estefanía-Fresco R, Marichalar-Mendia X, Aguirre-Urizar JM, Aguirre-Zorzano LA. Complete root coverage in the treatment of Miller class III or RT2 gingival recessions: a systematic review and meta-analysis. BMC Oral Health. 2021 Mar 22;21(1):145. doi: 10.1186/s12903-021-01494-3. Erratum in: BMC Oral Health. 2021 Apr 26;21(1):213. PMID: 33752657; PMCID: PMC7986294.

Imber JC, Kasaj A. Treatment of Gingival Recession: When and How? Int Dent J. 2021 Jun;71(3):178-187. doi: 10.1111/idj.12617. Epub 2021 Jan 29. PMID: 34024328; PMCID: PMC9275303.

Zucchelli G, Tavelli L, McGuire MK, Rasperini G, Feinberg SE, Wang HL, Giannobile WV. Autogenous soft tissue grafting for periodontal and peri-implant plastic surgical reconstruction. J Periodontol. 2020 Jan;91(1):9-16. doi: 10.1002/JPER.19-0350. Epub 2019 Oct 6. PMID: 31461778.

Zucchelli G, Mounssif I. Periodontal plastic surgery. Periodontol 2000. 2015 Jun;68(1):333-68. doi: 10.1111/prd.12059. PMID: 25867992.

Barootchi S, Tavelli L, Zucchelli G, Giannobile WV, Wang HL. Gingival phenotype modification therapies on natural teeth: A network meta-analysis. J Periodontol. 2020 Nov;91(11):1386-1399. doi: 10.1002/JPER.19-0715. Epub 2020 Jun 12. PMID: 32392401.

Cairo F, Nieri M, Pagliaro U. Efficacy of periodontal plastic surgery procedures in the treatment of localized facial gingival recessions. A systematic review. J Clin Periodontol. 2014 Apr;41 Suppl 15:S44-62. doi: 10.1111/jcpe.12182. PMID: 24641000.

Almeida VC, Pannuti CM, Ferreira MS, Lazarin RO, Romito GA, Jung RE, Tatakis DN, Silva COE, Cesar Neto JB. Conventional versus flap-protected free gingival graft: a multicenter randomized clinical trial. Braz Oral Res. 2023 Jan 6;37:e001. doi: 10.1590/1807-3107bor-2023.vol37.0001. PMID: 36629587.

Bitencourt FV, Cardoso De David S, Schutz JDS, Otto Kirst Neto A, Visioli F, Fiorini T. Minimizing patient morbidity after free gingival graft harvesting:A triple-blind randomized-controlled clinical trial. Clin Oral Implants Res. 2022 Jun;33(6):622-633. doi: 10.1111/clr.13923. Epub 2022 Apr 6. PMID: 35305280.

Naziker Y, Ertugrul AS. Aesthetic evaluation of free gingival graft applied by partial de-epithelialization and free gingival graft applied by conventional method: a randomized controlled clinical study. Clin Oral Investig. 2023 Jul;27(7):4029-4038. doi: 10.1007/s00784-023-05029-8. Epub 2023 Apr 28. PMID: 37118334.

Huang JP, Liu JM, Wu YM, Dai A, Hu HJ, He FM, Chen QM, Li XJ, Sun P, Ding PH. Clinical evaluation of xenogeneic collagen matrix versus free gingival grafts for keratinized mucosa augmentation around dental implants: A randomized controlled clinical trial. J Clin Periodontol. 2021 Oct;48(10):1293-1301. doi: 10.1111/jcpe.13518. Epub 2021 Jul 23. PMID: 34219259.

Pini Prato G, Di Gianfilippo R, Pannuti CM, Allen EP, Aroca S, Avila-Ortiz G, Bouchard P, Cairo F, Chackartchi T, Cortellini P, Franceschi D, Mahajan A, Mancini EA, McGuire MK, Natsvlishvili T, Santamaria MP, Scheyer ET, Wang HL,

Kornman K, Chambrone L. Diagnostic reproducibility of the 2018 Classification of Gingival Recession Defects and Gingival Phenotype: A multicenter inter- and intra-examiner agreement study. J Periodontol. 2023 May;94(5):661-672. doi: 10.1002/JPER.22-0501. Epub 2022 Dec 20. PMID: 36464773.

Hard tissues

Tooth extraction triggers a cascade of biological events mediated both by the local inflammatory response following surgery and by the loss of masticatory stimulation of the periodontium. These events disrupt the homeostasis and structural integrity of periodontal tissues.

During post-extraction healing, alveolar bone undergoes additional morphological changes as a result of natural remodeling. This begins immediately after extraction, with the greatest resorption occurring in the first two months, although dimensional changes may still be observed one year after surgery. Various studies have reported greater resorption of the buccal wall than of the lingual socket wall. Alveolar ridge preservation (ARP) is a surgical technique intended to minimize post-extraction dimensional changes. Several techniques can be used to preserve socket architecture after tooth extraction.

An autologous bone graft is defined as living tissue capable of promoting bone healing that is transferred from a donor site to a recipient site. It has been described as the "gold standard" because of its osteogenic, osteoinductive, and osteoconductive capacity and its ability to provide good structural support, with high biocompatibility and low immunogenicity. However, autologous bone availability is limited, and harvesting it from a donor site increases morbidity, surgical time, infection, inflammation, pain, and bleeding.

Patients with extensive bone loss resulting from remodeling require complex bone regeneration methods involving autogenous grafts or biomaterials. The conventional bone regeneration technique was conceptualized by Urban et al.[1] in 2013 and uses autogenous bone particles harvested from the patient's mandible mixed with anorganic bovine bone in a 1:1 ratio, covered by a resorbable collagen membrane secured to the patient's bone with tacks.

The horizontal bone regeneration technique using autogenous bone described by Khoury et al.[2] uses two bone blocks stabilized with miniscrews, with the defect filled with autogenous bone particles. The autogenous bone is harvested from areas such as the mandibular ramus, mandibular angle (retromolar region), or chin symphysis. The original Khoury technique uses a micromotor, saw, or piezoelectric device to harvest donor-site bone. The 2021 systematic review by Sánchez et al.[3] states: "The availability of RCTs comparing these techniques specifically is scarce, so it will be appropriate to design comparative studies in future in order to understand implant behavior and marginal bone loss with a longer follow-up".

Avila-Ortiz G, Chambrone L, Vignoletti F. Effect of alveolar ridge preservation interventions following tooth extraction: A systematic review and meta-analysis. J Clin Periodontol [Internet]. 2019 Jun 1 [cited 2023 Aug 19];46 Suppl 21(S21):195–223.

Horváth A, Mardas N, Mezzomo LA, Needleman IG, Donos N. Alveolar ridge preservation. A systematic review. Clin Oral Investig [Internet]. 2013 Mar 1 [cited 2023 Aug 5];17(2):341–63.

Mauricio G Araújo 1, Jan Lindhe, Dimensional ridge alterations following tooth extraction. An experimental study in the dog. Journal of Clinical Periodontology, 32(2), 212–218

Oreste Iocca, Alessio Farcomeni, Simon Pardiñas Lopez, Huzefa S. Talib. Alveolar ridge preservation after tooth extraction: a Bayesian Network meta-analysis of grafting materials efficacy on prevention of bone height and width reduction. Journal of Clinical Periodontology, 44(1), 104–114 | 10.1111/jcpe.12633.

Prevention of Bone Resorption by HA/β-TCP + Collagen Composite after Tooth Extraction: A Case Series. International Journal of Environmental Research and Public Health, 16(23), 4616

MacBeth N, Trullenque-Eriksson A, Donos N, Mardas N. Hard and soft tissue changes following alveolar ridge preservation: a systematic review. Clin Oral Implants Res [Internet]. 2017 Aug 1 [cited 2023 Aug 21];28(8):982–1004.

Majzoub J, Ravida A, Starch-Jensen T, Tattan M, Amo FSL del. The Influence of Different Grafting Materials on Alveolar Ridge Preservation: a Systematic Review. J Oral Maxillofac Res [Internet]. 2019 Sep 5 [cited 2023 Aug 5];10(3):3–6.

Atieh MA, Alsabeeha NH, Payne AG, Duncan W, Faggion CM, Esposito M. Interventions for replacing missing teeth: alveolar ridge preservation techniques for dental implant site development. Cochrane Database Syst Rev [Internet]. 2015 May 28 [cited 2023 Aug 5];2015(5).

Zhao R, Yang R, Cooper PR, Khurshid Z, Shavandi A, Ratnayake J. Bone Grafts and Substitutes in Dentistry: A Review of Current Trends and Developments. Molecules [Internet]. 2021 [cited 2023 Aug 5];26(10).

Zhao R, Yang R, Cooper PR, Khurshid Z, Shavandi A, Ratnayake J. Bone Grafts and Substitutes in Dentistry: A Review of Current Trends and Developments. Molecules [Internet]. 2021 [cited 2023 Aug 21];26(10).

Research objectives

General objective

To assess the effectiveness and feasibility of soft tissue and bone techniques to provide improved clinical knowledge that contributes to advances in dental practice.

Specific objectives

- Investigate and compare long-term root coverage outcomes using the modified free graft technique versus other available techniques, analyzing graft stability and patients' periodontal health.
- Evaluate and compare techniques for covering palatal graft donor sites in terms of morbidity with current versus digital techniques.
- Compare the effectiveness of vertical bone augmentation using autogenous bone plates with bone techniques using biomaterials.
- Quantify volumetric changes at different healing stages of post-extraction alveolar ridges treated with biomaterials.
- Develop practical recommendations to improve the application of soft tissue and bone grafting techniques based on the findings and results obtained during the research.

Research question or study hypothesis

Digital surgical protocols are as effective as conventional surgical protocols for dental tissue grafting in dental deficiencies and bone tissue grafting in edentulous ridges.

Methodology

Methodology I

Study type and population

The study has an interventional design involving clinical trials to be conducted at the Dentistry Clinic of Universidad Espíritu Santo in Guayaquil during 2024–2025.

All patients who underwent surgery were informed about the study and procedures and provided written consent.

A total of 30 patients with gingival recession defects (RT1) at mandibular incisors were evaluated, as in the article by Carcuac et al. All patients received comprehensive periodontal care, including dental prophylaxis, oral hygiene instructions, scaling and root planing, and coronal polishing. Surgery was performed only after a plaque index below 20% had been achieved.

Surgical procedure

After the initial examination, all patients received oral hygiene instructions and professional ultrasonic dental cleaning, including scaling and root planing with Gracey curettes and polishing with a rubber cup and fluoride paste.

Patients were randomly assigned to two groups: conventional free gingival graft (FGG, control group) or modified free gingival graft (ModFGG, experimental group). Randomization was computer-generated.

After local anesthesia with lidocaine, the exposed root surfaces were instrumented with Gracey curettes and rinsed with saline. Depending on group allocation, one of the following procedures was performed.

Experimental group with modified free gingival graft

Recipient-site preparation

An intrasulcular incision was made with a 15C scalpel blade, followed by bilateral horizontal incisions 2 mm long at the level of the cementoenamel junction. From these incisions, divergent vertical releasing incisions were extended into the alveolar mucosa. A thin split-thickness flap was then elevated, and the recipient site was measured.

Preparation of the connective tissue pedicle flap

Apical to the exposed root area, a horizontal incision was made at a distance corresponding to the height of the exposed root surface. Two slightly divergent incisions were added from its endpoints in a coronal direction. The connective tissue pedicle flap was thus outlined and carefully elevated from the periosteum. The pedicle graft, attached at its most coronal portion, was then turned over and positioned on the exposed root surface using laterally placed 6.0 resorbable sutures.

Free gingival graft harvesting

The graft dimensions were outlined on the palate adjacent to the premolars and first molar. A split-thickness graft consisting of epithelium and a thin layer of underlying connective tissue was harvested (intended total graft thickness: 1.5 mm), maintaining a distance of ≥2 mm from the maxillary teeth. After FGG harvesting, an acetate sheet was placed to cover the wound and achieve hemostasis.

Graft placement

The FGG was adapted to the recipient site and anchored to the periosteum with simple sutures (periosteal sutures). The lateral graft edges were sutured to provide stability. Where necessary, vertically suspended crossed sutures were placed to apply slight compression of the graft against the recipient site. No apical mucosal sutures were placed.

Control group with conventional free gingival graft

The surgical procedures used in the control group were the same as those in the experimental group, except that the connective tissue pedicle flap was omitted.

Postoperative follow-up and infection control

Postoperative pain and edema were managed with nonsteroidal anti-inflammatory medication and analgesics.

After surgery, patients were instructed to rinse twice daily with 0.2% chlorhexidine digluconate solution and avoid mechanical trauma, such as toothbrushing and excessive muscle traction, in the surgical area for two weeks.

Sutures were removed two weeks after surgery, and plaque control measures were resumed. Patients subsequently brushed with the modified Stillman technique using a soft-bristled toothbrush. Follow-up appointments were scheduled at one, three, and six months, as reported in the article by Carcuac et al. The final examination was performed one year after surgery.

Clinical measurements and patient-related outcomes

Clinical measurements were performed by an examiner at the mid-buccal aspect at baseline (one week before surgery) and at 1, 3, 6, and 12 months after surgery, as in the article by Carcuac et al.

The following variables were selected for assessment:

- Gingival recession depth at buccal and interproximal sites.
- Probing depth at buccal and interproximal sites.
- Clinical attachment level, subsequently calculated at buccal and interproximal sites.

A periodontist with more than five years of experience was contacted to evaluate study outcomes. The periodontist was informed about and trained in the new technique. All questions about the technique were clarified before the study began. The examiner evaluated recession depth, probing depth, and clinical attachment level at each gingival defect.

Data were collected using:

- Cone-beam computed tomography imaging (Samborondón Business Center, Imadent 3D).
- Visual assessment.
- Photographic records.
- Scanning.

Inclusion criteria

- At least one localized recession in the mandibular anterior region.
- Cairo recession type 1.
- Patients with good plaque control ≥15%.
- Patients aged 18–64 years.

Exclusion criteria

- Systemically compromised patients.
- Smoking more than 10 cigarettes per day.
- Pregnancy, breastfeeding, or oral contraceptive use.
- Medication that may affect healing or coagulation.
- Previous periodontal surgery in the gingival recession area.

Methodology II

The study has an interventional design comparing two alternative techniques for protecting the palatal donor site. All patients undergoing intervention will be informed about the study type, procedures, and interventions and will provide consent for decision-making and evaluations throughout the study.

A total of 30 patients will be included who have periodontal or peri-implant defects in at least two quadrants and require free gingival graft harvesting and use for periodontal or peri-implant surgery. The average graft size will span three to four teeth, from the maxillary canine to the maxillary first molar region.

Surgery will be performed in two operative stages using a split-mouth design within the same patient, with two groups: Group I, intervention using a self-adhesive periodontal dressing; and Group II, intervention using flowable resin. Both are intended to protect the palatal donor site.

Group I surgical technique

Local anesthesia is administered using lidocaine with epinephrine 1:80,000. A free gingival graft is harvested with a 15C scalpel blade at a thickness of 2 mm and a length spanning three to four teeth (between the maxillary canine and first molar). Hemostasis is achieved with gauze pressure and anesthetic containing a vasoconstrictor. A hydrolyzed collagen hemostatic sponge (Hemospon) is placed. The self-adhesive periodontal dressing is then cut and adapted to the wound shape, extending 2–3 mm beyond the margins. Crossed sutures are placed to hold the material in position and stabilize the clot. This technique modifies the manufacturer's recommended placement of the dressing above the sutures, which favors rapid loss of adhesion to the oral mucosa and early detachment. The purpose of the modification is to retain the dressing longer and provide greater palatal protection.

Group II surgical technique

The initial graft-harvesting steps are similar to those in the previous technique. This technique focuses on bonding to teeth. For selective enamel etching, 35% phosphoric acid is applied for 15 seconds to the palatal surfaces of the teeth involved, depending on graft size. The surfaces are rinsed with abundant water, and Single Bond Universal adhesive (3M) is applied. In both techniques, a hydrolyzed collagen hemostatic sponge (Hemospon) and crossed sutures are used to maintain and stabilize the clot, while bleeding is controlled with gauze pressure and anesthetic containing a vasoconstrictor. Flowable resin is then applied starting from the palatal tooth surfaces and completely covering the palatal wound.

The protective materials and sutures will be removed seven days after surgery to assess healing through granulation tissue formation and subsequent re-epithelialization.

Inclusion criteria

- Age over 18 years.
- Plaque score below 20%.
- Bleeding on probing below 10%.
- Need for two periodontal or peri-implant surgeries in the maxilla or mandible.
- No previous surgery at the donor site.

Exclusion criteria

- Pregnancy or breastfeeding.
- Palatal lesions.
- Use of a prosthesis covering the palate.
- Periodontal disease.
- Smoking.
- Systemic disease.

Methodology III

This randomized experimental study with parallel groups will be conducted in 78 adults requiring horizontal bone regeneration in edentulous posterior mandibular ridges. Surgical procedures will be performed at the Dentistry Clinic of Universidad de Especialidades Espíritu Santo (UEES), in Samborondón, Ecuador.

Each participant will be randomly assigned to one of three study groups. In all cases, bone regeneration will use the cortical shell technique (Khoury-type technique), filled with a mixture of 50% autogenous bone and 50% xenogeneic biomaterial, to enable subsequent implant-supported rehabilitation once the grafts have integrated.

Inclusion criteria

- Age over 18 years.
- Bilateral posterior mandibular edentulism.
- No previous surgery at the donor site.
- A maximum height of 9 mm from the inferior alveolar nerve to the superior mandibular border.
- No uncontrolled systemic disease.

Exclusion criteria

- Pregnancy.
- Uncontrolled systemic disease.
- Bisphosphonate or anticoagulant use.
- Allergy to penicillins.

Imaging assessment

A preoperative cone-beam computed tomography (CBCT) examination will be performed for comparison with immediate postoperative CBCT findings and an additional CBCT examination six months after surgery.

Clinical assessment

Before surgery, an accurate three-dimensional model of each patient's mandibular ridge dimensions will be obtained. This will be compared with a new scan immediately after surgery and another six months after rehabilitation.

Surgical procedure

Under local anesthesia, bone grafts are placed in the edentulous posterior mandibular regions. Using a No. 15 scalpel blade, an incision is made along the edentulous mandibular ridge, followed by elevation of a trapezoidal flap. The grafts described for the control and study groups will be placed in this space.

Control group with conventional Khoury technique

Autogenous bone graft: The surgical technique consists of placing two autogenous bone plates, 1 mm thick and with height and width adapted to each patient's needs, secured to the mandibular bone with two or three screws. The plates will be harvested from the patient's mandibular ridges and rami. Measurement and cutting guidance will be prepared using exocad software. Bone plates will be harvested with an ultrasonic device (Piezosurgery Touch, Mectron, Italy). The internal graft space will be filled with a mixture of 50% autogenous bone and 50% biomaterials (Bioss, Geistlich Pharma, Switzerland). Depending on the patient's needs, autogenous bone may be obtained from the chin, maxillary retromolar region, or mandibular ramus.

Study group 1 with modified Khoury technique

Autogenous bone graft: The modification replaces autogenous bone with two collagen-containing porcine cortical bone plates (1 mm rigid lamina, OsetoBiol by Tecnoss), secured to the mandibular ridge with two or three screws. The internal graft space will be filled with a mixture of 50% autogenous bone and 50% biomaterials (Bioss [text ends here in the source].

Study group 2 with conventional Khoury technique

Two human-derived cortical sheets (Maxgraft® Cortical Sheet, Botiss, Germany), previously rehydrated and adapted to the defect, will be placed and secured to the mandibular ridge with two or three screws. The internal space will be filled with the same mixture of 50% autogenous bone and 50% xenogeneic biomaterial (Bio-Oss®, Geistlich Pharma, Switzerland).

Methodology IV

Study type and population

This experimental study will involve 30 procedures in 30 different individuals. The study includes 30 people at the Dental Clinic of Universidad Espíritu Santo (UEES), Guayaquil, Ecuador. Thirty maxillary and mandibular tooth extractions were performed using an atraumatic extraction technique. Thirty sites were preserved with bioactive glass, and the socket will be closed with a resorbable collagen membrane retained with sutures.

Patients accepted the study terms and signed the informed consent form. Tomographic measurements were performed before extraction, after extraction, and two and six months after extraction to observe dimensional changes over time.

Surgical procedure

A tomographic scan is obtained of the area indicated for tooth extraction. Patients will undergo tomography before surgery, after extraction, and at two and six months to observe alveolar bone volume changes. The patient attends the UEES clinic for extraction. Appropriate asepsis and antisepsis procedures are followed before surgery. Once a sterile environment has been established, local anesthesia is administered using 2% lidocaine with epinephrine 1:80,000. Atraumatic extraction is performed to avoid damaging the bone plates. A 15C scalpel blade is used to sever the gingival fibers, and a periotome is introduced until it reaches the alveolar crest. The instrument is placed in the periodontal ligament space, and buccopalatal movements are made along the mesial or distal tooth surfaces. The periotome is advanced toward the root apex until two-thirds of the root length is reached or tooth mobility is achieved. The socket is then preserved with 58S bioactive glass (JHS ACTIVE BONE). A 15 × 20 mm resorbable collagen membrane (BIOHORIZONS, MEM-LOK) is placed beneath the buccal and palatal/lingual socket margins and sutured with 6.0 nylon using a 3/8-circle reverse-cutting needle. The membrane is retained with a horizontal mattress suture and a crossed suture. After surgery, the patient undergoes tomography on the same day to assess initial volumetric dimensions. Further scans are obtained at two and six months to observe volumetric changes at each healing stage. The implant is placed at six months.

Inclusion criteria

- Patients over 18 years of age.
- Nonsmokers.
- Patients with an indication for tooth extraction.

- Patients who have accepted the study conditions.

Exclusion criteria

- Pregnancy.
- Systemic disease.
- Previous traumatic extraction.
- Bisphosphonate or anticoagulant use.
- Patients who are not allergic to penicillins. [As stated in the source.]

Human and material resources

1. Person responsible for performing surgical procedures.

2. Person responsible for assessing the data obtained.

3. Person responsible for performing the evaluation.

Tomography equipment.

3D printer.

Ethical and gender considerations

Potential risk of infection: barrier methods, sterile instruments, and sterile gloves will be used. In the event of infection, a professional will always be available. The facility is in the same building as the UEES Clinic medical center, with an on-call physician, although infectious complications are not expected.

Patient data will be anonymized using the first letter of the given name, the first letter of the surname, and the initial digits of the national identity number.

No biological samples will be collected. Participants will receive no compensation and will not pay for the study. Use of the facilities costs $15. Those who require it will not pay any additional cost. Participants may withdraw without incurring any cost.

Informed consent

Depending on the type of research, the minimum requirements (Annex 3) for an informed consent document, whether broad (Annex 4) or specific, must be observed.

Notes

If research participants are minors or are unable to provide consent because of mental or behavioral disorders, an informed assent document is required in addition to informed consent signed by their legal representatives. The assent document must be adapted to the participants' level of maturity and their capacity to decide on participation.

Expected results

Provide details and a description of the potential benefits to the study population and the results expected from project implementation, considering the stated objectives. Highlight the relevance of the results and their potential field of application.

Discuss possible limitations and biases that could prevent the expected results from being achieved. Also explain how these limitations will be overcome to ensure the project's success.

Bibliographic references

Carcuac O, Trullenque-Eriksson A, Derks J. Modified-free gingival graft technique for treatment of gingival recession defects at mandibular incisors: A randomized clinical trial. J Periodontol. 2023 Jan 10. doi: 10.1002/JPER.22-0581. Epub ahead of print. PMID: 36627509.

Akcan SK, Ünsal B. Gingival recession treatment with concentrated growth factor membrane: a comparative clinical trial. J Appl Oral Sci. 2020 Mar 27;28:e20190236. doi: 10.1590/1678-7757-2019-0236. PMID: 32236353; PMCID: PMC7105285.

Cairo F, Nieri M, Pagliaro U. Efficacy of periodontal plastic surgery procedures in the treatment of localized facial gingival recessions. A systematic review. J Clin Periodontol. 2014;41(15):S44-62.

Ramos UD, Bastos GF, Costa CA, de Souza SLS, Taba M Jr, Novaes AB Jr. Root coverage with tunneling technique or modified advanced flap associated with acellular dermal matrix: results from 6 months randomized clinical trial. Clin Oral Investig. 2022 Jan;26(1):773-780. doi: 10.1007/s00784-02104055-8. Epub 2021 Aug 7. PMID: 34363104.

Pini Prato G, Clauser C, Cortellini P, Tinti C, Vincenzi G, Pagliaro U. Guided tissue regeneration versus mucogingival surgery in the treatment of human buccal recessions. A 4-year follow-up study. J Periodontol. 1996;67:1216-1223.

Fernández-Jiménez A, García-De-La-Fuente AM, Estefanía-Fresco R, Marichalar-Mendia X, Aguirre-Urizar JM, Aguirre-Zorzano LA. Complete root coverage in the treatment of Miller class III or RT2 gingival recessions: a systematic review and meta-analysis. BMC Oral Health. 2021 Mar 22;21(1):145. doi: 10.1186/s12903-021-01494-3. Erratum in: BMC Oral Health. 2021 Apr 26;21(1):213. PMID: 33752657; PMCID: PMC7986294.

Imber JC, Kasaj A. Treatment of Gingival Recession: When and How? Int Dent J. 2021 Jun;71(3):178-187. doi: 10.1111/idj.12617. Epub 2021 Jan 29. PMID: 34024328; PMCID: PMC9275303.

Zucchelli G, Tavelli L, McGuire MK, Rasperini G, Feinberg SE, Wang HL, Giannobile WV. Autogenous soft tissue grafting for periodontal and peri-implant plastic surgical reconstruction. J Periodontol. 2020 Jan;91(1):9-16. doi: 10.1002/JPER.19-0350. Epub 2019 Oct 6. PMID: 31461778.

Zucchelli G, Mounssif I. Periodontal plastic surgery. Periodontol 2000. 2015 Jun;68(1):333-68. doi: 10.1111/prd.12059. PMID: 25867992.

Barootchi S, Tavelli L, Zucchelli G, Giannobile WV, Wang HL. Gingival phenotype modification therapies on natural teeth: A network meta-analysis. J Periodontol. 2020 Nov;91(11):1386-1399. doi: 10.1002/JPER.19-0715. Epub 2020 Jun 12. PMID: 32392401.

Cairo F, Nieri M, Pagliaro U. Efficacy of periodontal plastic surgery procedures in the treatment of localized facial gingival recessions. A systematic review. J Clin Periodontol. 2014 Apr;41 Suppl 15:S44-62. doi: 10.1111/jcpe.12182. PMID: 24641000.

Almeida VC, Pannuti CM, Ferreira MS, Lazarin RO, Romito GA, Jung RE, Tatakis DN, Silva COE, Cesar Neto JB. Conventional versus flap-protected free gingival graft: a multicenter randomized clinical trial. Braz Oral Res. 2023 Jan 6;37:e001. doi: 10.1590/1807-3107bor-2023.vol37.0001. PMID: 36629587.

Bitencourt FV, Cardoso De David S, Schutz JDS, Otto Kirst Neto A, Visioli F, Fiorini T. Minimizing patient morbidity after free gingival graft harvesting:A triple-blind randomized-controlled clinical trial. Clin Oral Implants Res. 2022 Jun;33(6):622-633. doi: 10.1111/clr.13923. Epub 2022 Apr 6. PMID: 35305280.

Naziker Y, Ertugrul AS. Aesthetic evaluation of free gingival graft applied by partial de-epithelialization and free gingival graft applied by conventional method: a randomized controlled clinical study. Clin Oral Investig. 2023 Jul;27(7):4029-4038. doi: 10.1007/s00784-023-05029-8. Epub 2023 Apr 28. PMID: 37118334.

Huang JP, Liu JM, Wu YM, Dai A, Hu HJ, He FM, Chen QM, Li XJ, Sun P, Ding PH. Clinical evaluation of xenogeneic collagen matrix versus free gingival grafts for keratinized mucosa augmentation around dental implants: A randomized controlled clinical trial. J Clin Periodontol. 2021 Oct;48(10):1293-1301. doi: 10.1111/jcpe.13518. Epub 2021 Jul 23. PMID: 34219259.

Pini Prato G, Di Gianfilippo R, Pannuti CM, Allen EP, Aroca S, Avila-Ortiz G, Bouchard P, Cairo F, Chackartchi T, Cortellini P, Franceschi D, Mahajan A, Mancini EA, McGuire MK, Natsvlishvili T, Santamaria MP, Scheyer ET, Wang HL, Kornman K, Chambrone L. Diagnostic reproducibility of the 2018 Classification of Gingival Recession Defects and Gingival Phenotype: A multicenter inter- and intra-examiner agreement study. J Periodontol. 2023 May;94(5):661-672. doi: 10.1002/JPER.22-0501. Epub 2022 Dec 20. PMID: 36464773.

Chambrone L, Avila-Ortiz G. An evidence-based system for the classification and clinical management of non-proximal gingival recession defects. J Periodontol. 2021 Mar;92(3):327-335. doi: 10.1002/JPER.20-0149. Epub 2020 Aug 17. PMID: 32738056.

Pini-Prato G, Franceschi D, Cairo F, Nieri M, Rotundo R. Classification of dental surface defects in areas of gingival recession. J Periodontol. 2010 Jun;81(6):885-90. doi: 10.1902/jop.2010.090631. PMID: 20450362.

Cairo F, Nieri M, Cincinelli S, Mervelt J, Pagliaro U. The interproximal clinical attachment level to classify gingival recessions and predict root coverage outcomes: an explorative and reliability study. J Clin Periodontol. 2011 Jul;38(7):661-6. doi: 10.1111/j.1600-051X.2011.01732.x. Epub 2011 Apr 20. PMID: 21507033.

Cortellini P, Bissada NF. Mucogingival conditions in the natural dentition: Narrative review, case definitions, and diagnostic considerations. J Periodontol. 2018 Jun;89 Suppl 1:S204-S213. doi: 10.1002/JPER.16-0671. PMID: 29926948.

Pini-Prato G. The Miller classification of gingival recession: limits and drawbacks. J Clin Periodontol. 2011;38:243–245.

Tonetti MS, Jepsen S; Working Group 2 of the European Workshop on Periodontology. Clinical efficacy of periodontal plastic surgery procedures: consensus report of Group 2 of the 10th European Workshop on Periodontology. J Clin Periodontol. 2014 Apr;41 Suppl 15:S36-43. doi: 10.1111/jcpe.12219. PMID: 24640999.

Merijohn GK. Management and prevention of gingival recession. Periodontol 2000. 2016 Jun;71(1):228-42. doi: 10.1111/prd.12115. PMID: 27045439.

Kim DM, Neiva R. Periodontal soft tissue non-root coverage procedures: a systematic review from the AAP Regeneration Workshop. J Periodontol. 2015 Feb;86(2 Suppl):S56-72. doi: 10.1902/jop.2015.130684. PMID: 25644300.

Wennström J, Lindhe J, Nyman S. Role of keratinized gingiva for gingival health. Clinical and histologic study of normal and regenerated gingival tissue in dogs. J Clin Periodontol. 1981 Aug;8(4):311-28. doi: 10.1111/j.1600-051x.1981.tb02041.x. PMID: 6172452.

Meza-Mauricio J, Mourão ERST, Oliveira Marinho K, Vergara-Buenaventura A, Mendoza-Azpur G, Muniz FWMG, et al. Effect of collagen sponge and flowable resin composite on pain management after free gingival graft harvesting: A randomized controlled clinical trial. Eur J Oral Sci. 2023;131:e12935. https://doi.org/10.1111/eos.12935

Tavelli, Lorenzo; Ravidà, Andrea; Saleh, Muhammad H. A.; Maska, Bartosz; del Amo, Fernando Suárez-López; Rasperini, Giulio; Wang, Hom-Lay (2018). Pain perception following epithelialized gingival graft harvesting: a randomized clinical trial. Clinical Oral Investigations, (), –. doi:10.1007/s00784-018-2455-5

Arturo R, Reyes C, Patricia A, Casillas V. Tratamiento de recesiones gingivales mediante colgajo desplazado coronal modificado más injerto de tejido conectivo subepitelial. Serie de casos Trabajo de investigación [Internet]. Vol. 3, Núm. 1 Ene-Abr. 2012. Disponible en: www.medigraphic.org.mx

Kim DM, Neiva R. Periodontal Soft Tissue Non–Root Coverage Procedures: A Systematic Review From the AAP Regeneration Workshop. J Periodontol. febrero de 2015;86(2-s):S56-72.

Tavelli L, Barootchi S, Ravidà A, Oh TJ, Wang HL. What Is the Safety Zone for Palatal Soft Tissue Graft Harvesting Based on the Locations of the Greater Palatine Artery and Foramen? A Systematic Review. Vol. 77, Journal of Oral and Maxillofacial Surgery. W.B. Saunders; 2019. p. 271.e1-271.e9.

Sanz-Martín I, Rojo E, Maldonado E, Stroppa G, Nart J, Sanz M. Structural and histological differences between connective tissue grafts harvested from the lateral palatal mucosa or from the tuberosity area. Clin Oral Investig. 8 de febrero de 2019;23(2):957-64.

Yussif N, Wagih R, Selim K. Propylene mesh versus acrylic resin stent for palatal wound protection following free gingival graft harvesting: a short-term pilot randomized clinical trial. BMC Oral Health. 1 de diciembre de 2021;21(1).

Avila-Ortiz G, Chambrone L, Vignoletti F. Effect of alveolar ridge preservation interventions following tooth extraction: A systematic review and meta-analysis. J Clin Periodontol [Internet]. 2019 Jun 1 [cited 2023 Aug 19];46 Suppl 21(S21):195–223.

Horváth A, Mardas N, Mezzomo LA, Needleman IG, Donos N. Alveolar ridge preservation. A systematic review. Clin Oral Investig [Internet]. 2013 Mar 1 [cited 2023 Aug 5];17(2):341–63.

Mauricio G Araújo 1, Jan Lindhe, Dimensional ridge alterations following tooth extraction. An experimental study in the dog. Journal of Clinical Periodontology, 32(2), 212–218

Oreste Iocca, Alessio Farcomeni, Simon Pardiñas Lopez, Huzefa S. Talib. Alveolar ridge preservation after tooth extraction: a Bayesian Network meta-analysis of grafting materials efficacy on prevention of bone height and width reduction. Journal of Clinical Periodontology, 44(1), 104–114 | 10.1111/jcpe.12633.

Prevention of Bone Resorption by HA/β-TCP + Collagen Composite after Tooth Extraction: A Case Series. International Journal of Environmental Research and Public Health, 16(23), 4616

MacBeth N, Trullenque-Eriksson A, Donos N, Mardas N. Hard and soft tissue changes following alveolar ridge preservation: a systematic review. Clin Oral Implants Res [Internet]. 2017 Aug 1 [cited 2023 Aug 21];28(8):982–1004.

Majzoub J, Ravida A, Starch-Jensen T, Tattan M, Amo FSL del. The Influence of Different Grafting Materials on Alveolar Ridge Preservation: a Systematic Review. J Oral Maxillofac Res [Internet]. 2019 Sep 5 [cited 2023 Aug 5];10(3):3–6.

Atieh MA, Alsabeeha NH, Payne AG, Duncan W, Faggion CM, Esposito M. Interventions for replacing missing teeth: alveolar ridge preservation techniques for dental implant site development. Cochrane Database Syst Rev [Internet]. 2015 May 28 [cited 2023 Aug 5];2015(5).

Zhao R, Yang R, Cooper PR, Khurshid Z, Shavandi A, Ratnayake J. Bone Grafts and Substitutes in Dentistry: A Review of Current Trends and Developments. Molecules [Internet]. 2021 [cited 2023 Aug 5];26(10).

Zhao R, Yang R, Cooper PR, Khurshid Z, Shavandi A, Ratnayake J. Bone Grafts and Substitutes in Dentistry: A Review of Current Trends and Developments. Molecules [Internet]. 2021 [cited 2023 Aug 21];26(10).

Schmidt AH. Autologous bone graft: Is it still the gold standard? Injury [Internet]. 2021 Jun 1 [cited 2023 Aug 21];52 Suppl 2:S18–22.

Siaili M, Chatzopoulou D, Gillam DG. An overview of periodontal regenerative procedures for the general dental practitioner. Saudi Dent J [Internet]. 2018 Jan;30(1):26.

Fernández RF, Bucchi C, Navarro P, Beltrán V, Borie E. Bone grafts utilized in dentistry: an analysis of patients' preferences. BMC Med Ethics [Internet]. 2015 Oct 20

Baldini N, De Sanctis M, Ferrari M. Deproteinized bovine bone in periodontal and implant surgery. Dent Mater . 2011 Jan 27(1):61–70.

Iezzi G, Degidi M, Scarano A, Petrone G, Piattelli A. Anorganic bone matrix retrieved 14 years after a sinus augmentation procedure: a histologic and histomorphometric evaluation. J Periodontol. 2007 Oct, 78(10):2057–61.

Slotte C, Asklöw B, Lundgren D. Surgical guided tissue regeneration treatment of advanced periodontal defects: a 5-year follow-up study. J Clin Periodontol [Internet]. 2007;34(11):977–84.

Barone A, Toti P, Quaranta A, Alfonsi F, Cucchi A, Negri B, et al. Clinical and Histological changes after ridge preservation with two xenografts: preliminary results from a multicentre randomized controlled clinical trial. J Clin Periodontol. 2017 Feb 1;44(2):204–14.

Titsinides S, Agrogiannis G, Karatzas T. Bone grafting materials in dentoalveolar reconstruction: A comprehensive review. Japanese Dental Science Review. 2019 Nov 1;55(1):26–32.

Sawant K, Pawar A. Bioactive glass in dentistry: A systematic review. Saudi Journal of Oral Sciences [Internet]. 2020 [cited 2023 Aug 19];7(1):3.

Cannio M, Bellucci D, Roether JA, Boccaccini DN, Cannillo V. Bioactive Glass Applications: A Literature Review of Human Clinical Trials. Materials (Basel) [Internet]. 2021 Sep 1 [cited 2023 Aug 19];14(18).

Zohourfazeli M, Mahdi Tajer MH, Moghanian A. Comprehensive investigation on multifunctional properties of zirconium and silver co-substituted 58S bioactive glass. Ceram Int. 2021 Jan 15;47(2):2499–507.

Shahrbabak MSN, Sharifianjazi F, Rahban D, Salimi A. A Comparative Investigation on Bioactivity and Antibacterial Properties of Sol-Gel Derived 58S Bioactive Glass Substituted by Ag and Zn. Silicon. 2019 Dec 1;11(6):2741–51.

Moghanian A, Zohourfazeli M, Mahdi Tajer MH, Miri Z, Hosseini SM, Rashvand A. Preparation, characterization and in vitro biological response of simultaneous co-substitution of Zr+4/Sr+2 58S bioactive glass powder. Ceram Int. 2021 Sep 1;47(17):23762–9.

Khoury, F., & Hanser, T. (2015). Mandibular Bone Block Harvesting from the Retromolar Region: A 10-Year Prospective Clinical Study. The International Journal of Oral & Maxillofacial Implants, 30(3), 688–697.doi:10.11607/jomi.4117

Li S, Zhao J, Xie Y, Tian T, Zhang T, Cai X. Hard tissue stability after guided bone regeneration: a comparison between digital titanium mesh and resorbable membrane. Int J Oral Sci. 2021 Nov 16;13(1):37.

Final declaration

The research team, represented by the project Sponsor and Principal Investigator, freely and voluntarily declares the following:

- The content, authorship, and responsibility for the study results belong to the Sponsor and Principal Investigator, and the Ministry of Public Health is released from any legal action arising on these grounds.
- The project described in this document is original work whose authors are members of the research team. Full legal responsibility is therefore assumed should a third party claim ownership of the project's intellectual rights, and the Ministry of Public Health is released from any legal action arising on these grounds.
- This project causes no harm to research participants or the environment and violates no legal regulation or ethical standard. If the research requires permits from institutions other than the Ministry of Public Health, the Sponsor/Principal Investigator will submit certified copies to the Ministry of Public Health of Ecuador before implementation.
- They will ensure that the research is conducted under the terms approved by both the Human Research Ethics Committee and the Ministry of Public Health.
- A final research report containing the findings will be submitted to the Ministry of Public Health.

Place: City – Province
Date:

**Principal investigator**	**Sponsor**
Antonio Gabriel Lanata Flores	
Full name	Full name
Signature (required):	Signature (required):
0916524820	
National identity number or passport (required)	National identity number or passport (required)

Annex A

Project implementation schedule

This schedule summarizes project implementation over time and must follow a logical sequence of deadlines for activities under each specific objective. It must be consistent with the “Project implementation period” subsection of “General research information”, and with the “Project implementation period” subsection of this form.

Project:

Schedule periods shown in the original grid: 1–36. The table below lists the periods marked with an X for each activity; unlisted periods are unmarked.

Project	1	2	3	4	5	6	7	8	9	10	11	12	13	14	15	16	17	18	19	20	21	22	23	24	25	26	27	28	29	30	31	32	33	34	35	36
**Specific Objetive 1**																																				
**Activity 1.1**	x	x																																		
**Activity 1.2**			x	x																																
**Activity 1.3**			x	x	x	x																														
**Activity 1.4**					x	x	x																													
**Activity 1.5**						x	x	x	x																											
**Activity 1.6**									x	x	x																									
**Specific Objetive 2**																																				
**Activity 2.1**	x	x																																		
**Activity 2.2**			x	x	x																															
**Activity 2.3**				x	x	x	x																													
**Activity 2.4**							x	x	x	x																										
**Activity 2.5**										x	x	x																								
**Specific Objetive 3**																																				
**Activity 3.1**	x	x																																		
**Activity 3.2**			x	x	x																															
**Activity 3.3**					x	x																														
**Activity 3.4**						x	x	x	x	x	x	x																								
**Activity 3.5**							x	x	x	x	x	x	x																							
**Activity 3.6**													x	x																						
**Activity 3.7**														x	x	x																				
**Activity 3.8**															x	x	x	x																		
**Specific Objetive 4**																																				
**Activity 4.1**	x	x	x	x	x	x	x	x	x	x	x	x	x	x	x	x																				
**Activity 4.2**															x	x	x																			
**Activity 4.3**															x	x	x	x	x																	
**Activity 4.4**																			x	x	x															

Annex B

Participating institutions

Field	Information
Institution	UEES
Public / private	Private
Postal address	km 2.5, road to Samborondón
Contact person	Antonio Lanata Flores
Contact email	alanataf@uees.edu.ec
Contact telephone	(+593) 983820084

Annex C

Operational definitions of variables

Bone tissue

Field	Description
Definition	Bone tissue is a specialized connective tissue that forms the main structure of bones in humans and other vertebrates. It consists mainly of specialized cells called osteocytes embedded in a mineralized extracellular matrix composed primarily of calcium and phosphate salts.[1]
Dimension	Amount of newly formed bone at different postoperative stages
Indicator	Measurement from the superior border of the maxillary ridge to the inferior alveolar nerve
Scale	mm
Type	Quantitative

Gingival tissue

Field	Description
Definition	A type of soft tissue that surrounds the teeth and protects the bones supporting them. The gingiva consists mainly of dense fibrous connective tissue and is covered externally by keratinized epithelium, which protects it from injury and microbial invasion.[2]
Dimension	Amount of newly formed soft tissue at different postoperative stages
Indicator	Measurement of soft tissue margins around teeth
Scale	
Type	Quantitative

Socioeconomic and demographic characteristics

Field	Description
Definition	"Socioeconomic" refers to the relationship between the social and economic aspects of a society, including income, education, social class, employment, and living conditions. The term is used to analyze how these factors interact and influence different aspects of individuals' and communities' lives.[3] "Sociodemographic" refers to demographic aspects related to a population's social structure, such as age, gender, ethnicity, educational level, and marital status. Sociodemographic analysis examines how these factors influence population dynamics and living conditions.[4]
Dimension	
Indicator	Age measured from birth to current age
Scale	Numerical
Type	Quantitative

Pain

Field	Description
Definition	Pain is an unpleasant sensory and emotional experience associated with actual or potential tissue damage. It is an important alarm signal indicating injury or disease requiring attention. Pain may be acute, occurring immediately after injury, or chronic, persisting for weeks, months, or years.[5]
Dimension	Postoperative pain measured using a visual analog scale from 1 to 10
Indicator	
Scale	
Type	Quantitative

Satisfaction

Field	Description
Definition	Satisfaction is a positive emotional state resulting from the perception that a person's expectations or wishes have been fulfilled concerning a particular experience, situation, or relationship. It may relate to different aspects of life, including work, interpersonal relationships, and personal achievements.
Dimension	
Indicator	
Scale	
Type	Quantitative

Bibliographic references for Annex C

1. Moore, K. L., Dalley, A. F., & Agur, A. M. R. (2014). Anatomía con orientación clínica (7a ed.). Lippincott Williams & Wilkins.

2. Newman, M. G., Takei, H. H., Klokkevold, P. R., & Carranza, F. A. (2015). Carranza's clinical periodontology (12th ed.). Saunders.

3. Galbraith, J. K. (1996). Economía y el bien común. Ediciones Península.

4. López-Gay, A. (2005). Sociología de la población: Fundamentos teóricos y metodológicos. Plaza y Valdés.

5. Loeser, J. D., Butler, S. H., Chapman, C. R., & Turk, D. C. (Eds.). (2001). Bonica's Management of Pain (3rd ed.). Lippincott Williams & Wilkins.

6. Diener, E., Lucas, R. E., & Oishi, S. (Eds.). (2018). Advances in subjective well-being research. Springer.

ANEXO 12

Formulario para la presentación de protocolos de investigaciones de observacionales y de intervención en seres humanos
(Excepto Ensayos Clínicos)

INSTRUCCIONES: *El siguiente formulario deberá ser llenado completamente, en idioma español empleando letra tipo Times New Roman de 10 puntos, a espacio sencillo, en hojas tamaño A4, manteniendo un margen de 2,5 cm por lado. Si alguna de las tablas del formulario requiere de más filas, puede crearlas; sin embargo, debe tener en consideración los límites de texto que puede ingresar en algunas secciones del formulario. No debe excederse de 20 páginas. La argumentación debe apoyarse en referencias bibliográficas y datos estadísticos actualizados, mismos que deberán ser citadas en el texto utilizando un número de*

DATOS GENERALES DE LA INVESTIGACIÓN

TÍTULO

Comportamiento clínico en la regeneración de tejidos blandos y óseos de la cavidad oral de pacientes con ausencia y defectos dentarios en la clínica odontológica de la Universidad Espíritu Santo desde 2024 hasta el 2029

TIPO DE INVESTIGACIÓN			
Marque con una X la opción que corresponda			
**Estudios Observacionales**		**Estudios de Intervención**	
- Estudios Transversales		- Estudios Cuasi-experimentales	
- Estudios Ecológicos		- Ensayo de campo	
- Reportes de Casos		- Ensayos controlados aleatorizados sin medicamentos y/o dispositivos móviles	x
- Series de Casos			
- Estudios de casos y Controles			
- Estudios de Cohorte			
**Otros**			
Especifique			

TIEMPO DE EJECUCIÓN DE LA INVESTIGACIÓN

60 meses
1ero de Mayo 2024 hasta 30 de Abril 2029

FINANCIAMIENTO DE LA INVESTIGACIÓN

Monto total del financiamiento de la investigación	*122410,80*
Fuentes de financiamiento	*Centro de investigación - Universidad de Especialidades Espíritu Santo*

DATOS DEL PATROCINADOR

(Patrocinador es la persona natural o jurídica, compañía, institución, empresa u organización incluidas las académicas, legalmente constituidas y con representación legal en el país, que tiene la responsabilidad sobre la iniciación, manejo, desarrollo y financiamiento de la investigación)

Nombre de la persona/institución que realiza la investigación

**Patrocinador**	*Universidad Espíritu Santo*			**Cédula/RUC**	
**Teléfono Institucional**	*(593) - 045000950*	**Extensión**		**Correo Electrónico**	
**Dirección**	*km. 2,5 via a samborondon*				
**Página Web Institucional**	www.uees.edu.ec				
**Órgano Ejecutor**	*Centro de investigación - Universidad de Especialidades Espíritu Santo*				

COBERTURA DE EJECUCIÓN DE LA INVESTIGACIÓN

(Seleccione sólo un tipo de cobertura)

**Nacional**	☐		
**Zonas de Planificación**	☐	Zona 1 (Carchi, Esmeraldas, Imbabura y Sucumbíos)	☐
		Zona 2 (Napo, Orellana y Pichincha)	☐
		Zona 3 (Chimborazo, Cotopaxi, Pastaza y Tungurahua)	☐
		Zona 4 (Manabí, Sto. Domingo de los Tsáchilas)	☐
		Zona 5 (Bolívar, Guayas, Los Ríos y Santa Elena)	☐
		Zona 6 (Azuay, Cañar y Morona Santiago)	☐
		Zona 7 (El Oro, Loja y Zamora Chinchipe)	☐
		Zona 8 (Cantones Guayaquil, Samborondón, Durán)	☐
		Zona 9 (Distrito Metropolitano de Quito)	☐
**Provincial**	☐	*Especifique las provincias en las que se ejecutará su investigación*	
**Local**	■	*Espefique la Provincia y Cantones donde se ejecutará su investigación*	

PERSONAL DE LA INVESTIGACIÓN

NOTA: *Debe incluirse a todos los investigadores.*
Si es necesario añada una fila por cada miembro del equipo científico-técnico de la investigación, en caso que el patrocinador sea un investigador se debe repetir su nombre en ambas filas.

FUNCIÓN	NOMBRE COMPLETO	CÉDULA DE CIUDADANÍA/PASAPORTE	FORMACIÓN ACADÉMICA	ENTIDAD A LA QUE PERTENECE	CORREO ELECTRÓNICO PERSONAL E INSTITUCIONAL	TELÉFONO CELULAR
Patrocinador						

Investigador Principal	Antonio Gabriel Lanata Flores	0916524820	Odontólogo	UEES	alanataf@uees.edu.ec	0983820084
Investigador 1						
Investigador 2						
Técnico/Asistente						

DETALLE DE LA INVESTIGACIÓN

Resumen estructurado

El resumen deberá contener los siguientes apartados (Máximo 1 página):

1. *Título*
2. *Introducción*
3. *Objetivos*
4. *Métodos*
5. *Resultados esperados*

Problema de investigación

Los pacientes que presentan perdidas dentarias usualmente tienen disfunciones. Las afecciones funcionales se deben a que las pérdidas de las piezas dentarias alteran la oclusión, disminuyen la estabilidad de las piezas dentarias y existe una constante remodelación ósea en el área edéntula.

Las áreas edéntulas sufren de una progresiva remodelación ósea que, en muchos casos, dificultan la rehabilitación ósea. En la actualidad, la rehabilitación de primera opción en áreas edéntulas es con instalación de implantes dentarios. En pacientes con grandes pérdidas óseas como consecuencia de la remodelación ósea, se debe realizar métodos de regeneración ósea complejas que incluye de técnicas óseas con injertos autógenos o con uso de biomateriales. La técnica convencional de regeneración ósea fue conceptualizada por Urban et al.1 en 2013 en donde utilizan una mezcla de partículas autógenas de hueso obtenido en mandíbula del paciente y hueso anorgánico de origen bovino combinadas en una proporción 1:1, recubiertos por una membrana absorbible de colágeno posicionada con tachuelas en el hueso del paciente.

La técnica de regeneración ósea horizontal con hueso autógeno descrita por Khoury et al.2 se la realiza con el uso de dos bloques óseos estabilizados con mini tornillos y rellenando el defecto con partículas de hueso autógeno. La obtención del hueso autógeno se lo realiza en áreas como rama de la mandíbula, ángulo mandibular (zona retromolar) o sínfisis del mentón. La técnica original de Khoury obtiene el hueso de área donadora con el uso de micromotor, sierra o piezoeléctrico. En la revisión sistemáticas de Sánchez., et al.3 del 2021 menciona ¨The availability of RCTs comparing these techniques specifically is scarce, so it will be appropriate to design comparative studies in future in order to understand implant behavior and marginal bone loss with a longer follow-up¨.
En la fase de curación después de la extracción, el hueso alveolar sufre cambios morfológicos adicional como resultado del proceso de remodelación natural. Esto comienza inmediatamente después de la extracción, mostrando mayor cantidad de reabsorción los primeros dos meses después de la extracción, aunque se puede observar cambios dimensionales 1 año después del acto quirúrgico. Diferentes estudios dieron como resultado un mayor grado de reabsorción en la pared bucal en comparación con la pared lingual del alveolo. Sobre los tejidos blandos en áreas donadoras, existe un solo reporte de ensayo clínico controlado aleatorizado que valida este tipo de técnica con resina fluida con esponjas hemostáticas de colágeno y ausencia de estudios sobre apósitos autoadhesivos como protección palatina, lo cual se desea comprobar su utilización como técnicas alternativas para la protección del paladar luego de la toma de un injerto gingival libre y valorar la cicatrización y dolor posoperatorio

PROBLEMA:

PROBLEMA:

No existe un consenso en beneficios e indicaciones entre las técnicas de regeneración de tejidos blandos y tejidos óseos.
Hay limitaciones en los trabajos de regeneración ósea con follow-up inadecuados.

•Urban, I.A.; Lozada, J.L.; Jovanovic, S.A.; Nagy, K. Horizontal guided bone regeneration in the posterior maxilla using recombinant human platelet-derived growth factor: A case report. Int. J. Periodontics Restor. Dent. 2013, 33, 421–425.
•Khoury, F.; Hanser, T. Mandibular bone block harvesting from the retromolar region: A 10-year prospective clinical study. Int. J. Oral Maxillofac. Implants 2015, 30, 688–697.
•Sánchez-Sánchez,J.; Pickert, F.N.; Sánchez-Labrador, L.; GF Tresguerres, F.; Martínez- González, J.M.; Meniz-García, C. Horizontal Ridge Augmentation: A Comparison between Khoury and Urban Technique. Biology 2021, 10, 749.
•Horváth A, Mardas N, Mezzomo LA, Needleman IG, Donos N. Alveolar ridge preservation. A systematic review. Clin Oral Investig [Internet]. 2013 Mar 1 [cited 2023 Aug 5];17(2):341–63.
•Mauricio G Araújo 1, Jan Lindhe, Dimensional ridge alterations following tooth extraction. An experimental study in the dog. Journal of Clinical Periodontology, 32(2), 212–218
•Oreste Iocca, Alessio Farcomeni, Simon Pardiñas Lopez, Huzefa S. Talib. Alveolar ridge preservation after tooth extraction: a Bayesian Network meta-analysis of grafting materials efficacy on prevention of bone height and width reduction. Journal of Clinical Periodontology, 44(1), 104–114 | 10.1111/jcpe.12633.
•Meza-Mauricio J, Mourão ERST, Oliveira Marinho K, Vergara-Buenaventura A, Mendoza-Azpur G, Muniz FWMG, et al. Effect of collagen sponge and flowable resin composite on pain management after free gingival graft harvesting: A randomized controlled clinical trial. Eur J Oral Sci. 2023;131:e12935. https://doi.org/10.1111/eos.12935Cairo F, Pagliaro

Justificación

La regeneración ósea por medio de tablas óseas y uso de micro tornillos es un procedimiento de alta precisión, pero de bajo costo. Siendo muy interesante poder desarrollar un protocolo quirúrgico viable para los cirujanos dentistas especializados en el área de la implantología. Adicionalmente se ha demostrado que el uso de vidrio bioactivo es una alternativa para la reparación y sustitución de tejido óseo. Los cambios volumétricos óseos se ven disminuidos en su mayoría a los 2 y 6 meses después de la extracción dental según lo mencionado en el articulo de Araujo y Lindhe. Durante la preservación alveolar, el cirujano debe fijarse en eliminar todo el tejido de granulación que queda en el alveolo, si no se elimina correctamente puede afectar la efectividad de la preservación alveolar, por lo contrario, hay investigaciones que demuestran ventajas del vidrio bioactivo por sus componentes antimicrobianos. En la actualidad existe escasa o nula evidencia científica acerca de este biomaterial injertado en alveolo post-extracción

Los injertos tejidos blandos presentan una queja algica muy fuerte en los pacientes, por los que se busca disminuir la morbilidad posoperatoria, tiempo quirúrgico y mejorar la cicatrización de tejidos blandos luego de la remoción de un injerto gingival libre y su colocación en áreas que necesiten recubrimientos.

Marco Teórico

TEJIDOS BLANDOS

Las recesiones gingivales (RG) se pueden definir como la exposición de la superficie de la raíz después de una migración apical del margen gingival en relación con la línea amelo-cementaria.(1) Las RGs afectan tanto a las poblaciones más jóvenes como a las mayores, diversas encuestas revelaron que el 88% de las personas ≥65 años y el 50% de las personas de 18 a 64 años tienen ≥1 sitio con recesión gingival.(1-20). Las RGs pueden ocurrir por factores anatómicos, patológicos o por trauma y va a dar como consecuencia una pérdida de inserción. (2)

La exposición de la superficie de la raíz por la RG se asocia con frecuencia con la hipersensibilidad a la dentina, caries radiculares, las lesiones cervicales no cariosas (NCCL), el bajo control de la placa y la apariencia poco estética. (7)

Las indicaciones para los procedimientos de cobertura de la raíz incluyen las demandas de los pacientes para mejorar la estética y la reducción de la hipersensibilidad de la raíz, pero también un interés en el aumento de las medidas de tejido queratinizado. (3)

Siempre se ha sostenido a la idea de que es necesaria la presencia de encía queratinizada para mantener la salud gingival. Aunque la cantidad exacta de tejido queratinizado (TQ) necesario para la salud periodontal sigue en duda, doctores están de acuerdo en que ≥ 1 mm de la encía es necesaria para mantener la salud periodontal en pacientes con un control de placa óptimo. (24)

Lang y Loe propusieron que es necesarios ≥ 2 mm de TQ alrededor de los dientes para mantener la salud periodontal del paciente con una higiene promedio. El consenso actual es que ≥2 mm de TQ y ≥ 1 mm de encía adherida es necesario alrededor de los dientes. (25)

La recesión gingival es una de las preocupaciones estéticas más comunes asociadas con los tejidos periodontales. La clasificación de dicha condición es importante para diagnosticar, determinar el pronóstico y poder realizar un correcto plan de tratamiento.

En cirugía plástica periodontal, el diagnóstico correcto de los defectos de las recesiones gingivales (DRG) es de crucial importancia para definir la necesidad de terapia, elegir enfoques de tratamiento adecuados y monitorear o predecir longitudinalmente cambios de sitios tratados versus no tratados (16)

de sitios tratados versus no tratados (16)

Muchas clasificaciones diagnósticas de DRGs han sido propuesto a lo largo de los años. Entre ellos, el sistema de clasificación de Miller ha sido el más emblemático en la práctica clínica durante décadas. Sin embargo, este sistema presentaba variables limitadas. (17-18).

Una nueva clasificación fue propuesta en el 2018 por Cairo et al. y se basaba en el nivel de inserción clínica interdental. (19)

La clasificación de Cairo es una clasificación orientada al tratamiento para pronosticar el potencial de cobertura radicular a través de la evaluación de CAL interdental. En Cairo RT1 (Miller Clase I y II) se puede predecir una cobertura de raíces del 100%; en Cairo RT2 (Clase III de Miller) algunos ensayos clínicos aleatorizados indican el límite de pérdida de CAL interdental dentro del cual es predecible una cobertura radicular del 100% aplicando diferentes procedimientos de cobertura radicular; en Cairo RT3 (clase IV de Miller) no se puede lograr una cobertura total de la raíz. (21-22)
Esta clasificación supera algunas limitaciones de la clasificación de Miller, como la difícil identificación entre Clase I y II, y el uso de "pérdida de tejido blando o hueso" como referencia interdental para diagnosticar una destrucción periodontal en el área interdental. Además, La clasificación de Miller se propuso cuando las técnicas de cobertura radicular estaban recién empezando y el pronóstico de la cobertura radicular en las cuatro clases de Miller ya no coincide con los resultados del tratamiento de las técnicas quirúrgicas más avanzadas.(21)

El clínico debe centrarse en los factores de susceptibilidad y las condiciones modificables asociadas con la recesión gingival. Los principales factores de susceptibilidad son tejido gingival delgado, ausencia o una banda estrecha (<2 mm) de KT, profundidades de sondaje que se extienden más allá de la MGJ y una historia positiva de recesión gingival y/o enfermedad periodontal inflamatoria. Las condiciones modificables son la acumulación de placa, la enfermedad periodontal inflamatoria, la posición aberrante del frenillo, los hábitos de higiene oral traumáticos, los márgenes de restauración subgingival, el tabaquismo y las condiciones sistémicas como la diabetes. Por lo tanto, el clínico debe identificar estos factores y, si es posible, modificarlos en consecuencia.(23)

Numerosas técnicas de cirugía plástica periodontal han sido sugeridas para el tratamiento de RGs. El injerto de tejido conectivo

(CTG) con colgajo avanzado hacia coronal(CAF), obteniendo ganancia de tejido queratinizado y logrando resultados de tratamiento predecibles (2). Otro procedimiento muy utilizado es la técnica de túnel, la cual se refiere a que se emplea un colgajo supraperiosteo mejorando la vascularizad del colgajo e injerto. (4)

Existen otras técnicas cuando se tiene que manejar condiciones mucogingivales desfavorables como la inserción marginal del frenillo, un vestíbulo poco profundo entre otros y, se ha propuesto el injerto gingival libre (IGL) (5). El IGL es un injerto de tejido blando extraído del paladar con el epitelio suprayacente (8-12). Una de las principales indicaciones de IGL es restablecer un tejido queratinizado adecuado y espesor gingival en presencia de defectos mucogingival. (8-9)

Se ha informado una gran variabilidad en términos de cobertura radicular subsiguiente (rango: 11%–87%; media: 72%) al aplicar esta técnica. Uno de los desafíos de esta técnica es el suministro de sangre limitado debido al hecho de que parte del injerto se coloca en un sitio receptor avascular, es decir, la superficie de la raíz expuesta (5).

Recientemente, Carcuac y Derks propusieron una versión modificada del IGL (IGLMod), en la que se voltea un colgajo de pedículo conectivo desde su origen apical para cubrir la superficie de la raíz expuesta antes del posicionamiento del IGL. (6-10)

La razón detrás del enfoque es mejorar la revascularización del injerto. Según la evidencia científica se puede lograr una cobertura completa de la raíz (CRC) en todos los pacientes a los 3 meses, Sin embargo, faltan datos de ensayos que comparen IGLMod con IGL convencional y que evalúen la eficacia a largo plazo de esta nueva técnica. (1-11)

El injerto autólogo de tejido conectivo (ITC) actualmente se considera como el gold standard para el tratamiento de defectos de tejido blando periodontal y peri-implantar. Inicialmente fue utilizado para aumentar el volumen de la cresta edéntula, el grosor de la encía queratinizada y profundizar el fondo del vestíbulo. Posteriormente, ha sido utilizado para numerosos procedimientos como la cobertura radicular, el aumento de tejidos blandos alrededor de implantes dentales y áreas parcialmente edéntulas en áreas estéticas, la reconstrucción de la papila y la corrección de cicatrices, su uso ha brindado un mejor pronóstico y mayor estabilidad a largo plazo de los tejidos blandos periodontales y peri-implantares.

Para la selección del área donadora de un injerto de tejido conectivo (ITC) debemos haber realizado un correcto diagnóstico para poder elegir el tipo de técnica y procedimiento a realizar, de acuerdo a las características biológicas; la mucosa palatina y la tuberosidad son áreas idóneas como donantes de un ITC, éstos poseen efectos beneficiosos con una genética inherente y factores tisulares que ayudan a mejorar el proceso de cicatrización y corrección de defectos. La zona de la tuberosidad se ha considerado como un sitio de recolección mejorado para injertos fibrosos de mayor grosor, ya que posee una mayor cantidad de lámina propia y menor de tejido submucoso (glandular y adiposo), más recomendados para el aumento de volumen en defectos de tejidos duros y blandos peri-implantarios. El uso de un ITC de la mucosa palatina ha reportado resultados exitosos en el tratamiento de deficiencias mucosas, se ha demostrado que el origen y composición biológica del injerto de la tuberosidad puede favorecer respuestas hiperplásicas y comprometer áreas estéticas.

La obtención de un ITC del área palatina ha sido descrita como la opción más recomendada para defectos mucosos, debido que para la obtención del mismo tenemos una mejor visibilidad y acceso, el tamaño del paladar nos favorece a que el diámetro y longitud del injerto requerido sea mayor en comparación con el de la tuberosidad.

Para la remoción de un ITC del paladar pueden haber varias técnicas en la cual éste puede quedar protegido mediante el epitelio como es el caso de un injerto de tejido conectivo subepitelial (ITCSE) o desprotegido sin epitelio como en un injerto gingival libre (IGL), en comparación de estas técnicas se destaca el IGL por la facilidad del diseño del injerto, el tamaño y ventajas en su remoción, pero con mayores molestias posoperatorias como dolor, riesgo de infección, y posibles sangrados excesivos, y su cicatrización se logrará por segunda intención, sin embargo, en un ITCSE el paciente presentará menores molestias ya que el cierre de la herida se logrará por primera intención, pero se trata de una técnica más complicada y en ocasiones no se logra obtener un grosor adecuado del injerto

La lesión accidental de un tronco vascular principal o de sus ramas colaterales es la causa más frecuente de hemorragia palatina. Debido a la separación de los bordes de la herida y sin permitir una aproximación de los mismos, la cicatrización y epitelialización se logrará por segunda intención en un tiempo prolongado que puede tardar aproximadamente de 2 a 4 semanas en cerrar el defecto. El grado más alto de dolor del sitio donador manifestado por los pacientes es el primer día posterior a la cirugía, disminuyendo sus valores en 2 semanas posquirúrgicas.

La cicatrización del área donadora del paladar se produce mediante la proliferación de fibroblastos, síntesis de colágeno, angiogénesis y contracción de la herida. Además, la revascularización, la inmunidad y la proliferación de células epiteliales son factores cruciales para una cicatrización óptima de las heridas. En un reto poder acelerar el proceso de cicatrización, reducir la hemorragia prolongada, el dolor causado por la herida palatina, se han utilizado diversos tipos de materiales para reducir la morbilidad del paciente como agentes hemostáticos (por ejemplo, colágeno sintético absorbible, esponja de gelatina absorbible, cianoacrilato, celulosa regenerada oxidada, subsulfato férrico), apósitos periodontales, apósitos autoadhesivos, protección mecánica como retenedores Hawley o termoplastificados, y más recientemente se han utilizado concentrados de plaquetas.

El uso de apósitos periodontales ha demostrado ser eficaz para lograr una reducción significativa del dolor, incomodidad, sangrado y permitir las actividades rutinarias del paciente. A pesar de esto, problemas tales como la fricción con la mucosa palatina durante la función pueden resultar en su desprendimiento causando sangrado asociado con dolor, retraso de la cicatrización y dificultad para hablar.

Un apósito autoadhesivo a base de polímeros hidrofílicos y celulosa hidroxyethil libres de eugenol permiten la adherencia a superficies intraorales para lograr proteger heridas y favorecer una mejor cicatrización de los tejidos absorbiendo el exudado inflamatorio generado en las primeras fases de cicatrización y ayudando a la proliferación de factores de crecimiento, estos apósitos pueden ser estabilizados sobre las heridas y el tiempo de adhesión va a depender del área y las condiciones del lecho quirúrgico. Obteniendo como resultado ventajas posoperatorias en el control del dolor, sangrado y cicatrización.

En la actualidad gracias a la diversidad de materiales restauradores adhesivos podemos conformar una nueva barrera de protección fija para el área donadora palatina; mediante técnicas adhesivas en piezas dentarias vecinas logrando una capa protectora de resina superficial, recubriendo el área denudada y favoreciendo la cicatrización por medio del mantenimiento del coágulo, esto favorecerá la hemostasia de la zona, reducción de morbilidad del paciente (dolor, edema, infección, necrosis)

Carcuac O, Trullenque-Eriksson A, Derks J. Modified-free gingival graft technique for treatment of gingival recession defects at mandibular incisors: A randomized clinical trial. J Periodontol. 2023 Jan 10. doi: 10.1002/JPER.22-0581. Epub ahead of print. PMID: 36627509.

Akcan SK, Ünsal B. Gingival recession treatment with concentrated growth factor membrane: a comparative clinical trial. J Appl Oral Sci. 2020 Mar 27;28:e20190236. doi: 10.1590/1678-7757-2019-0236. PMID: 32236353; PMCID: PMC7105285.

Cairo F, Nieri M, Pagliaro U. Efficacy of periodontal plastic surgery procedures in the treatment of localized facial gingival recessions. A systematic review. J Clin Periodontol. 2014;41(15):S44-62.

Ramos UD, Bastos GF, Costa CA, de Souza SLS, Taba M Jr, Novaes AB Jr. Root coverage with tunneling technique or modified advanced flap associated with acellular dermal matrix: results from 6 months randomized clinical trial. Clin Oral Investig. 2022 Jan;26(1):773-780. doi: 10.1007/s00784-02104055-8. Epub 2021 Aug 7. PMID: 34363104.

Pini Prato G, Clauser C, Cortellini P, Tinti C, Vincenzi G, Pagliaro U. Guided tissue regeneration versus mucogingival surgery in the treatment of human buccal recessions. A 4-year follow-up study. J Periodontol. 1996;67:1216-1223.

Fernández-Jiménez A, García-De-La-Fuente AM, Estefanía-Fresco R, Marichalar-Mendia X, Aguirre-Urizar JM, Aguirre-Zorzano LA. Complete root coverage in the treatment of Miller class III or RT2 gingival recessions: a systematic review and meta-analysis. BMC Oral Health. 2021 Mar 22;21(1):145. doi: 10.1186/s12903-021-01494-3. Erratum in: BMC Oral Health. 2021 Apr 26;21(1):213. PMID: 33752657; PMCID: PMC7986294.

Imber JC, Kasaj A. Treatment of Gingival Recession: When and How? Int Dent J. 2021 Jun;71(3):178-187. doi: 10.1111/idj.12617. Epub 2021 Jan 29. PMID: 34024328; PMCID: PMC9275303.

Zucchelli G, Tavelli L, McGuire MK, Rasperini G, Feinberg SE, Wang HL, Giannobile WV. Autogenous soft tissue grafting for periodontal and peri-implant plastic surgical reconstruction. J Periodontol. 2020 Jan;91(1):9-16. doi: 10.1002/JPER.19-0350. Epub 2019 Oct 6. PMID: 31461778.

Zucchelli G, Mounssif I. Periodontal plastic surgery. Periodontol 2000. 2015 Jun;68(1):333-68. doi: 10.1111/prd.12059. PMID: 25867992.

Barootchi S, Tavelli L, Zucchelli G, Giannobile WV, Wang HL. Gingival phenotype modification therapies on natural teeth: A network meta-analysis. J Periodontol. 2020 Nov;91(11):1386-1399. doi: 10.1002/JPER.19-0715. Epub 2020 Jun 12. PMID: 32392401.

Cairo F, Nieri M, Pagliaro U. Efficacy of periodontal plastic surgery procedures in the treatment of localized facial gingival recessions. A systematic review. J Clin Periodontol. 2014 Apr;41 Suppl 15:S44-62. doi: 10.1111/jcpe.12182. PMID: 24641000.

Almeida VC, Pannuti CM, Ferreira MS, Lazarin RO, Romito GA, Jung RE, Tatakis DN, Silva COE, Cesar Neto JB. Conventional versus flap-protected free gingival graft: a multicenter randomized clinical trial. Braz Oral Res. 2023 Jan 6;37:e001. doi: 10.1590/1807-3107bor-2023.vol37.0001. PMID: 36629587.

Bitencourt FV, Cardoso De David S, Schutz JDS, Otto Kirst Neto A, Visioli F, Fiorini T. Minimizing patient morbidity after free gingival graft harvesting:A triple-blind randomized-controlled clinical trial. Clin Oral Implants Res. 2022 Jun;33(6):622-633. doi: 10.1111/clr.13923. Epub 2022 Apr 6. PMID: 35305280.
Naziker Y, Ertugrul AS. Aesthetic evaluation of free gingival graft applied by partial de-epithelialization and free gingival graft applied by conventional method: a randomized controlled clinical study. Clin Oral Investig. 2023 Jul;27(7):4029-4038. doi: 10.1007/s00784-023-05029-8. Epub 2023 Apr 28. PMID: 37118334.
Huang JP, Liu JM, Wu YM, Dai A, Hu HJ, He FM, Chen QM, Li XJ, Sun P, Ding PH. Clinical evaluation of xenogeneic collagen matrix versus free gingival grafts for keratinized mucosa augmentation around dental implants: A randomized controlled clinical trial. J Clin Periodontol. 2021 Oct;48(10):1293-1301. doi: 10.1111/jcpe.13518. Epub 2021 Jul 23. PMID: 34219259.
Pini Prato G, Di Gianfilippo R, Pannuti CM, Allen EP, Aroca S, Avila-Ortiz G, Bouchard P, Cairo F, Chackartchi T, Cortellini P, Franceschi D, Mahajan A, Mancini EA, McGuire MK, Natsvlishvili T, Santamaria MP, Scheyer ET, Wang HL, Kornman K, Chambrone L. Diagnostic reproducibility of the 2018 Classification of Gingival Recession Defects and Gingival Phenotype: A multicenter inter- and intra-examiner agreement study. J Periodontol. 2023 May;94(5):661-672. doi: 10.1002/JPER.22-0501. Epub 2022 Dec 20. PMID: 36464773.

TEJIDOS DUROS

La extracción dental desencadena una cascada de eventos biológicos, mediados tanto por la respuesta inflamatoria local que sigue a la intervención quirúrgica como por la privación de la estimulación masticatoria del periodonto, que provocan una alteración de la homeostasis y la integridad estructural de los tejidos periodontales.

En la fase de curación después de la extracción, el hueso alveolar (HA) sufre cambios morfológicos adicional como resultado del proceso de remodelación natural. Esto comienza inmediatamente después de la extracción, mostrando mayor cantidad de reabsorción los primeros dos meses después de la extracción, aunque se puede observar cambios dimensionales 1 año después del acto quirúrgico. Diferentes estudios dieron como resultado un mayor grado de reabsorción en la pared bucal en comparación con la pared lingual del alveolo. La preservación del reborde alveolar (PRA) es una técnica quirúrgica que tiene como objetivo minimizar el grado de cambios dimensionales posteriores a la extracción. Se pueden usar varias técnicas para preservar la arquitectura del alvéolo después de la extracción del diente.

Un injerto óseo autólogo se define como un tejido vivo capaz de promover la cicatrización ósea, trasladando ese tejido de un área donadora a un área receptora. Se ha definido como el ¨estándar de oro¨ por tener capacidad de osteogénesis, osteoinducción, osteoconducción y proveer buen soporte estructural, mostrando alta biocompatibilidad y baja inmunogenicidad, sin embargo, el hueso autólogo es limitado y posee mayor morbilidad por la recolección del hueso autólogo del sitio donante, aumento del tiempo quirúrgico, mayor infección, inflamación, dolor y sangrado.

En pacientes con grandes pérdidas óseas como consecuencia de la remodelación ósea, se debe realizar métodos de regeneración ósea complejas que incluye de técnicas óseas con injertos autógenos o con uso de biomateriales. La técnica convencional de regeneración ósea fue conceptualizada por Urban et al.1 en 2013 en donde utilizan una mezcla de partículas autógenas de hueso obtenido en mandíbula del paciente y hueso anorgánico de origen bovino combinadas en una proporción 1:1, recubiertos por una membrana absorbible de colágeno posicionada con tachuelas en el hueso del paciente.

La técnica de regeneración ósea horizontal con hueso autógeno descrita por Khoury et al.2 se la realiza con el uso de dos bloques óseos estabilizados con mini tornillos y rellenando el defecto con partículas de hueso autógeno. La obtención del hueso autógeno se lo realiza en áreas como rama de la mandíbula, ángulo mandibular (zona retromolar) o sínfisis del mentón. La técnica original de Khoury obtiene el hueso de área donadora con el uso de micromotor, sierra o piezoeléctrico. En la revisión sistemáticas de Sánchez., et al.3 del 2021 menciona ¨ The availability of RCTs comparing these techniques specifically is scarce, so it will be appropriate to design comparative studies in future in order to understand implant behavior and marginal bone loss with a longer follow-up¨.

Avila-Ortiz G, Chambrone L, Vignoletti F. Effect of alveolar ridge preservation interventions following tooth extraction: A systematic review and meta-analysis. J Clin Periodontol [Internet]. 2019 Jun 1 [cited 2023 Aug 19];46 Suppl 21(S21):195–223.
Horváth A, Mardas N, Mezzomo LA, Needleman IG, Donos N. Alveolar ridge preservation. A systematic review. Clin Oral Investig [Internet]. 2013 Mar 1 [cited 2023 Aug 5];17(2):341–63.
Mauricio G Araújo 1, Jan Lindhe, Dimensional ridge alterations following tooth extraction. An experimental study in the dog. Journal of Clinical Periodontology, 32(2), 212–218
Oreste Iocca, Alessio Farcomeni, Simon Pardiñas Lopez, Huzefa S. Talib. Alveolar ridge preservation after tooth extraction: a Bayesian Network meta-analysis of grafting materials efficacy on prevention of bone height and width reduction. Journal of Clinical Periodontology, 44(1), 104–114 | 10.1111/jcpe.12633.
Prevention of Bone Resorption by HA/β-TCP + Collagen Composite after Tooth Extraction: A Case Series. International Journal of Environmental Research and Public Health, 16(23), 4616
MacBeth N, Trullenque-Eriksson A, Donos N, Mardas N. Hard and soft tissue changes following alveolar ridge preservation: a systematic review. Clin Oral Implants Res [Internet]. 2017 Aug 1 [cited 2023 Aug 21];28(8):982–1004.
Majzoub J, Ravida A, Starch-Jensen T, Tattan M, Amo FSL del. The Influence of Different Grafting Materials on Alveolar Ridge Preservation: a Systematic Review. J Oral Maxillofac Res [Internet]. 2019 Sep 5 [cited 2023 Aug 5];10(3):3–6.
Atieh MA, Alsabeeha NH, Payne AG, Duncan W, Faggion CM, Esposito M. Interventions for replacing missing teeth: alveolar ridge preservation techniques for dental implant site development. Cochrane Database Syst Rev [Internet]. 2015 May 28 [cited 2023 Aug 5];2015(5).
Zhao R, Yang R, Cooper PR, Khurshid Z, Shavandi A, Ratnayake J. Bone Grafts and Substitutes in Dentistry: A Review of Current Trends and Developments. Molecules [Internet]. 2021 [cited 2023 Aug 5];26(10).

Objetivos de la investigación

General:
Valorar la eficacia y viabilidad de técnicas de tejidos blandos y óseos para proporcionar mejores conocimientos clínicos que contribuyan al avance de las prácticas odontológicas
Específicos:
•Investigar y comparar los resultados a largo plazo de la cobertura radicular utilizando la técnica de injerto libre modificada versus otras técnicas disponibles, analizando la estabilidad del injerto y la salud periodontal de los pacientes.
•Evaluar y comparar las técnicas de recubrimiento de areas de injerto palatino acorde la morbidad de las técnicas actuales versus digitales.
•Comparar la eficacia de la técnica de aumento oseo vertical con tablas oseas autogenas y técnicas osea con uso de biomaterial.
•Cuantificar los cambios volumétricos observados en las diferentes etapas de cicatrización de bordes alveolares postexodoncia con uso de biomaterales
•Desarrollar recomendaciones prácticas para mejorar la aplicación de la técnica de injertos de tejidos blandos y óseos basadas en los hallazgos y resultados obtenidos durante la investigación

Pregunta de investigación o hipótesis del estudio

Los protocolos quirúrgicos digitales son eficaces como los protocolos quirúrgicos convencionales en injertos de tejidos dentarios en deficiencias dentarias e injertos de tejidos óseo en bordes edéntulos.

Metodología

METODOLOGÍA I

Tipo de estudio y población

El diseño de este estudio es de tipo intervención; ensayos clínicos que se lo realizará en la Clínica de odontología de la Universidad Espiritu Santo de Guayaquil en el periodo del año 2024-2025.

Todos los pacientes que fueron intervenidos quirurgicamente fueron informados sobre el estudio, los procedimientos y dieron su consentimiento por escrito.

Se evaluaron un total de 30 pacientes que tenian defectos de recesión gingival (RT1) en incisivos mandibulares como en el artículo de Carcuac y cols. Todos los pacientes recibieron atención periodontal integral con profilaxis dental, instrucciones de higiene oral, raspado y alisado radicular y pulido coronal. Las cirugías se realizaron solo después de que se lograra un índice de placa menor al 20 %.

Procedimiento quirúrgico

Después del examen inicial, todos los pacientes recibieron instrucciones de higiene oral, la cual consistia en una limpieza dental profesional con ultrasonido e incluía raspado y alisado con curetas Gracey y pulido con copa de goma y pasta con flúor.
Los pacientes fueron asignados aleatoriamente a dos grupos: injerto gingival libre convencional (IGL, grupo control) o injerto gingival libre modificado (IGLMod, grupo experimental).La aleatorización, se generó por computadora.

Despues de la anestesia local con lidocaina, las superficies radiculares expuestas se instrumentaron con curetas Gracey y se enjuagaron con solución salina. Según la asignación de grupos, se realizó uno de los procedimientos siguientes:

Grupo experimental (Injerto gingival libre modificado)

•Preparación del sitio receptor
Se empezó con una incisión intrasulcular con una hoja de bisturi 15 C, se realizaron bilateralmente incisiones horizontales de 2 mm de largo al nivel de LAC. A partir de estos, se realizaron incisiones de liberación verticales de manera divergente, extendiéndose hacia la mucosa alveolar. Posteriormente se levantó un colgajo delgado de espesor parcial. Se midió la dimensión del sitio receptor.

•Preparación del colgajo pedículado de tejido conectivo:
Apicalmente al área de la raíz expuesta de los dientes, se realizó una incisión horizontal a una distancia correspondiente a la altura de la superficie de la raíz expuesta. Desde los puntos finales de esta incisión y en dirección coronal, se agregaron dos incisiones ligeramente divergentes. Así, se delineó el colgajo pediculado de tejido conectivo y luego se levantó cuidadosamente del periostio. Luego, el injerto pedicular, adherido en su parte más coronal, se volteó y se colocó sobre la superficie de la raíz expuesta a través de suturas reabsorbibles 6.0 ubicadas lateralmente.

•Obtención del injerto gingival libre:
Se delinearon las dimensiones del injerto en el paladar adyacente a los premolares y al primer molar. Se obtuvo un injerto de espesor parcial que consistía en epitelio y una capa delgada de tejido conectivo subyacente (grosor total previsto del injerto: 1,5 mm), manteniendo una distancia de ≥2 mm a los dientes superiores. Después de la recolección de la IGL, se colocó una lámina de acetato para cubrir herida y hacer hemostasia.
•Colocación del injerto
El IGL se adaptó al sitio receptor y anclado al periostio mediante suturas simples (suturas periósticas). Se suturaron los bordes laterales del injerto para obtener estabilidad. Cuando fue necesario, se colocaron suturas cruzadas suspendidas verticalmente para lograr una ligera compresión del injerto al sitio receptor. No se realizó sutura apical de la mucosa.

Grupo control (Injerto gingival libre convencional):
Los procedimientos quirúrgicos aplicados en el grupo de control fueron iguales a los del grupo experimental, con la excepción de que se omitió el colgajo pediculado de tejido conectivo.

SEGUIMIENTO POSTQUIRÚRGICO Y CONTROL DE INFECCIONES
El dolor y el edema postoperatorios se controlaron con medicación antiinflamatoria no esteroidea y analgésicos.

Después de las intervenciones quirúrgicas, se indicó a los pacientes que se enjuagaran dos veces al día con una solución de digluconato de clorhexidina al 0,2% y que evitaran cualquier traumatismo mecánico como el de cepillado de dientes y tracción muscular excesiva en el área quirúrgica durante 2 semanas.

Se retiraron las suturas 2 semanas despues de la cirugía y se restablecieron las medidas de control de placa. Posteriormente, el cepillado se realizó mediante la técnica de Stillman modificada con un cepillo de dientes de cerdas suave. A los pacientes se les programaron citas de control a los 1, 3 y 6 meses como en el artículo de Carcuac y cols menciona. La examinación final se realizó 1 año después de la cirugía.

MEDICIONES CLÍNICAS Y RESULTADOS RELACIONADOS CON EL PACIENTE.

Las mediciones clínicas fueron realizadas por un examinador en la cara media por vestibular al inicio del estudio (1 semana antes de la intervención quirúrgica), así como 1, 3, 6 y 12 meses después de la cirugía al igual que el artículo de Carcuac y cols.

Se hizo una selección de variables a evaluar y estas fueron:
Profundidad de recesión gingival (PRG) tanto en los sitios bucales como interproximales.
Profundidad de sondaje (PS) tanto en los sitios bucales como interproximales.
Luego se calculó el nivel de inserción clínico (NIC) tanto en los sitios bucales como interproximales.

Se contactó a un periodoncista con más de 5 años de experiencia para evaluar los resultados del estudio. Se le informó y enseñó la nueva técnica a utilizar. Se le aclaró todas las dudas sobre la nueva técnica antes de comenzar el estuido. El examinador evaluó cada variable; PR, PS, NIC en cada defecto gingival.
La toma de recolección de datos se la hizo de la siguiente manera:
Toma de imágenes Tomografía computarizada cone bean (Samborondon bussines center Imadent 3D)
Otro método de valoración : visual
Registro fotográfico
Scanner

Los criterios de inclusión fueron:
Presencia de al menos una recesión localizada es sector antero-inferior
Recesión tipo 1 de Cairo
Pacientes que tengan buen control de placa ≥ 15%
Pacientes entre 18- 64 años

Los criterios de exclusión fueron :
Paciente comprometido sistémicamente
Paciente fumador más de 10 cigarrillos al día
Mujeres embarazadas, lactancia o uso de anticonceptivos orales
Pacientes que estén tomando medicación que pueda afectar la cicatrización y coagulación
Cirugías periodontales previas en el área de la recesión gingival

METODOLOGÍA II
El diseño de este estudio es de tipo intervención y comparativo entre dos técnicas alternativas para la protección del área donadora palatina. Todos los pacientes a intervenir serán informados sobre el tipo de estudio, procedimientos, intervenciones y los cuales brindarán su consentimiento para la toma de decisiones y evaluaciones durante el proceso del estudio.

Se tomarán en cuenta un total de 30 pacientes que presenten defectos periodontales o peri-implantares mínimo en dos cuadrantes, que tengan la necesidad de ser tratados mediante la recolección y uso de injertos gingivales libres para procedimientos de cirugía periodontal o peri-implantar; con un tamaño de injerto promedio que involucre de 3 a 4 piezas dentarias en región de canino superior a primer molar superior.

Las cirugías se realizarán en dos tiempos operatorios, con boca dividida en un mismo paciente, y serán divididos en dos grupos. Grupo I: intervención con utilización de un apósito periodontal autoadhesivo y Grupo II: intervención con utilización de resina fluida, ambos con el fin de proteger el área donadora palatina.

Técnica quirúrgica Grupo I: se aplica anestesia local con lidocaína y epinefrina al 1:80.000, se recolecta el injerto gingival libre con el uso de hoja de bisturí 15c y grosor de 2 mm, con una longitud de injerto entre 3 a 4 dientes (entre canino superior y primer molares superior), se realiza hemostasia bajo presión con gasa y uso de anestésico con vasoconstrictor, se coloca una esponja hemostática de colágeno hidrolizado (Hemospon), posterior aquello se corta y se adapta el apósito periodontal autoadhesivo a la forma de la cavidad entre 2 a 3 mm sobrepasando los márgenes y se realiza suturas en X para mantener el material en su posición y estabilizar el coágulo, esta técnica ha sido modificada de acuerdo a las recomendaciones del fabricante del producto ya que el apósito debe ser colocado por encima de la sutura lo que favorece su rápido pérdida de adhesión a la mucosa oral y su pronta caída y el fin de su modificación es mantener por mayor tiempo este apósito brindando mayores ventajas en la protección del paladar.

Técnica quirúrgica Grupo II: los primeros pasos en la recolección del injerto son similares a la técnica anterior, esta técnica tiene un enfoque en la adhesión dental, por medio del grabado ácido selectivo del esmalte se coloca ácido fosfórico al 35% durante 15 segundos en las caras palatinas de piezas dentarias involucradas de acuerdo al tamaño del injerto, se lava con abundante agua, se realiza la aplicación de (adhesivo single bond universal 3M), en ambas técnicas se coloca una esponja hemostática de colágeno hidrolizado (Hemospon), suturas en X para mantener y estabilizar el coágulo, Controlando la hemorragia con gasas a presión y anestésico con vasoconstrictor, posterior se procede a la colocación de resina fluida empezando desde las caras palatinas de las piezas dentarias, y llenando totalmente el tamaño de la herida palatina.

Estos materiales de protección y suturas utilizadas serán removidos a los 7 días posoperatorios para valorar la cicatrización por medio del tejido de granulación formado y su re-epitelización respectiva.

Criterios de Inclusión
Mayor de 18 años
Control de placa bacteriana menor 20%
Sangrado al sondaje menor del 10%
Pacientes con necesidad de dos cirugías periodontales o peri-implantares en maxilar superior o inferior
No tener cirugías previas del área donadora

Criterios de Exclusión
Mujeres embarazadas o en lactancia
Pacientes con lesiones en el paladar
Pacientes que usen prótesis que cubran el paladar
Pacientes con enfermedad periodontal
Pacientes fumadores
Paciente con enfermedades sistémicas

METODOLOGÍA III
Este estudio experimental aleatorizado de grupos paralelos se realizará en 78 personas adultas que requieran regeneración ósea horizontal en rebordes posteriores edéntulos mandibulares. Los procedimientos quirúrgicos se realizarán en la clínica odontológica de la Universidad de Especialidades Espíritu Santo (UEES), ciudad de Samborondón, Ecuador.

Cada participante será asignado aleatoriamente a uno de tres grupos de estudio. En todos los casos se realizará técnica de regeneración ósea mediante la técnica de láminas corticales (técnica tipo Khoury), rellenadas por una mezcla de 50% hueso autógeno y 50% biomaterial xenógeno, con el fin de permitir rehabilitaciones posteriores sobre implantes una vez integrados los injertos.

Criterio de inclusion
Mayor de 18 años
Pacientes con edentulismo posterior mandibular bilateral
No tener cirugías previas del área donadora
Altura máxima del nervio alveolar inferior al borde superior mandibular máxima de 9 mm
Sin enfermedades sistémicas no controladas
Criterios de exclusión:
Pacientes embarazadas
Pacientes con enfermedades sistemicas no controladas
Pacientes que tomen bifosfonatos o anticoagulantes
Paciente alergico a las penicilinas
Evaluación de imagen
Se realizará un examen de imagén tomografico computadorizado cone beam preoperatorio (C.B.C.T.) para comparar con los resultados de examen de imagen (C.B.C.T.) postoperatorios inmeditos y un examen adicional de imagen (C.B.C.T.) de postoperatorio de 6 meses

Evaluación clínico
Previo al acto quirurgico, todos los pacientes tendran un modelo tridimensional exacto de las medidas de sus rebordes mandibulares. Esto será comparado con un nuevo escaneo postoperatorio inmediato y uno posterior a 6 meses de rehabilitación

Procedimiento quirúrgico
Sobre anestesia local, se realiza la instalación de injertos oseos en regiones edentulas posteriores de mandibula. Con una lamina de bisturí 15, se realiza incision sobre el borde mandibular edentula para posterior realizar un colgajo trapezoidal. Sobre este espacio se colocará los injertos mencionados en los grupos controles y estudio.

Grupo Control (Técnica Khoury convencional)

Injerto óseo autógeno: La técnica quirugica consite en instalar 2 tablas oseas autogenas con un ancho de 1 mm y una altura y ancho acorde al caso necesitado por cada paciente fijadas con 2 o 3 tornillos al hueso mandibular. Las tablas oseas serán obtenidas de rebordes y ramas mandibulares del mismo paciente. La medición y guía de corte será confeccionada por un software exocad. La obtención de las tablas óseas será realizadas por medio de un ultrasonido (Piezosurgery Touch Mectron – Italia). La area interna de los injertos será rellenada por una mezcla de 50 % hueso autógeno y 50% de biomateriales (Bioss Geistlich Pharma – Suiza). La obtención del hueso autogeno, podrá ser de región mentoniana , retromolar maxilar o de la rama mandibular del paciente acorde a sus necesidades.

Grupo Estudio #1 (Técnica Khoury modificada): Injerto óseo autógeno: La modificación de la técnica consiste de reemplazar el uso de hueso autógeno por el uso de 2 tablas oseas de hueso cortical porcino con colageno (Lámina rigida 1 mm, OsetoBiol by Tecnoss) fijadas con 2 o 3 tornillos al reborde mandibular. La area interna de los injertos será rellenada por una mezcla de 50 % hueso autógeno y 50% de biomateriales (Bioss

Grupo Estudio #2 (Técnica Khoury convencional)
Se instalarán dos láminas corticales de origen humano (Maxgraft® Cortical Sheet, Botiss – Alemania), previamente rehidratadas y adaptadas al defecto, fijadas con 2 o 3 tornillos al reborde mandibular. El espacio interno será rellenado con la misma mezcla de 50% hueso autógeno y 50% biomaterial xenógeno (Bio-Oss®, Geistlich Pharma – Suiza).
METODOLOGÍA IV

Tipo de estudio y población
En este estudio experimental se va a realizar 30 procedimientos en 30 personas diferentes. El estudio incluye a 30 personas. En la clínica dental de la Universidad Espíritu Santo (UEES), ciudad de Guayaquil, Ecuador. Se realizaron 30 procedimientos de extracción dental maxilar y mandibular con técnica de extracción dental atraumática, de los cuales 30 sitios fueron preservados con vidrio bioactivo y se cerrara el alveolo la membrana de colágeno reabsorbible manteniéndola con suturas.

Los pacientes aceptaron los términos del estudio y firmaron el consentimiento informado. Se realizaron mediciones tomográficas antes de realizar la extracción, post-extracción, 2 y 6 meses después de la extracción dental para observar los cambios dimensionales que ocurren durante el tiempo.

Procedimiento quirúrgico

Se realiza una tomografía de la zona de la exodoncia dental indicada. Se realizara tomografía a los pacientes antes de la cirugía, post-extraccion y a los 2 y 6 meses para observar los cambios volumétricos del hueso alveolar. El paciente acude a la clínica UEES para realizar la extracción. Se aplican los pasos debidos de asepsia y antisepsia antes del acto quirúrgico, una vez que tengamos un ambiente estéril procedemos con anestesia local lidocaína 2% con epinefrina 1:80.000. Empezamos con la extracción atraumática para no dañar tablas óseas, utilizando una hoja de bisturí 15c se logra la ruptura de fibras gingivales, ingresamos el periotomo hasta llegar a cresta alveolar, el instrumento se coloca en el espacio del ligamento periodontal y se realizan movimientos vestibulares a palatinos en las superficies mesiales o distales del diente a extraer insertando el periotomo hacia el ápice radicular hasta alcanzar 2/3 de longitud radicular o hasta alcanzar movilidad dental, se procede a preservar el alveolo con el vidrio bioactivo 58s de la marca comercial JHS ACTIVE BONE , luego posicionamos la membrana de colágeno reabsorbible de la marca comercial BIOHORIZONS (MEM-LOK) con la medida de 15mm x 20mm por debajo de los bordes del alveolo vestibular y palatino/lingual y suturamos con sutura nylon 6.0 con una aguja de corte reverso 3/8, mantenemos la membrana de colágeno reabsorbible con la técnica de sutura colchonero horizontal y un punto en X. Una vez terminado el acto quirúrgico el paciente se realizará el mismo día una tomografía para observar las dimensiones volumétricas iniciales, luego se realizará tomografía a los 2 y 6 meses para observar los cambios volumétricos en cada etapa de cicatrización. A los 6 mes se hace la colocación del implante

Criterios de inclusión:
Pacientes mayores de 18 años de edad
No fumadores
Pacientes con exodoncias indicadas
Pacientes que hayan aceptado las condiciones del estudio
Criterios de exclusión:
Pacientes embarazadas
Pacientes con enfermedades sistemicas
Exodoncia traumática previa
Pacientes que tomen bifosfonatos o anticoagulantes
Pacientes que no sea alérgico a las penicilinas

Recursos humanos y materiales

1.- Responsable de realizar los procedimientos quirúrgicos.
2.- Responsable de evaluar los datos obtenidos
3.- Responsable de realizar la evaluación
tomografo
impresora 3d

Consideraciones éticas y de género

Posibles riesgo infección, métodos de barrera, instrumentales estériles,guantes estériles. En caso de infección, un profesional siempre estara disponble. Esta en el mismo edificio del centro medico uees clinic. con medico de guardia, aunque no se espera tener procesos infecciosos.
Se anomización datos de los pacientes Primera letra de nombre, primera letra de apellido, primeros dígitos de cédula.
No se realizan muestran biológicas. No va recibir ni una compensacion, pero tampoco va pagar por el estudio. El uso de las instalaciones tiene un costo de $15. Las personas que necesiten, no pagaran ni un costo adicional. Si desean salir, pueden y sin costo alguno

Consentimiento informado

Dependiendo del tipo de investigación, se deben precautelar las consideraciones mínimas (Anexo 3) que debe tener un documento de consentimiento informado ya sea amplio (Anexo 4) o específico.

Notas:
o En caso de que los participantes de la investigación sean menores de edad o que no esté en la capacidad de hacerlo debido a trastornos mentales o de conducta, a más del Consentimiento Informado firmado por sus representantes legales, es necesario presentar un documento de Asentimiento Informado el cual deberá ser adaptado al nivel de madurez de los participantes y a la capacidad de decidir sobre su participación.

Resultados esperados

Realizar un detalle y descripción de los posibles beneficios a la población sujeta de estudio, y de los resultados que se espera obtener con la ejecución del proyecto, considerando los objetivos quesehanplanteadoparaelmismo. Es importante que se destaque la relevancia de los resultados, así como el campo en el cual tendríanaplicabilidad.
Discutir posibles limitaciones y sesgos que podrían impedir que consiga los resultados. Además explique cómo superar estas limitaciones con el propósito que el proyecto sea exitoso.

Referencias citadas

Carcuac O, Trullenque-Eriksson A, Derks J. Modified-free gingival graft technique for treatment of gingival recession defects at mandibular incisors: A randomized clinical trial. J Periodontol. 2023 Jan 10. doi: 10.1002/JPER.22-0581. Epub ahead of print. PMID: 36627509.
Akcan SK, Ünsal B. Gingival recession treatment with concentrated growth factor membrane: a comparative clinical trial. J Appl Oral Sci. 2020 Mar 27;28:e20190236. doi: 10.1590/1678-7757-2019-0236. PMID: 32236353; PMCID: PMC7105285.
Cairo F, Nieri M, Pagliaro U. Efficacy of periodontal plastic surgery procedures in the treatment of localized facial gingival recessions. A systematic review. J Clin Periodontol. 2014;41(15):S44-62.
Ramos UD, Bastos GF, Costa CA, de Souza SLS, Taba M Jr, Novaes AB Jr. Root coverage with tunneling technique or modified advanced flap associated with acellular dermal matrix: results from 6 months randomized clinical trial. Clin Oral Investig. 2022 Jan;26(1):773-780. doi: 10.1007/s00784-02104055-8. Epub 2021 Aug 7. PMID: 34363104.
Pini Prato G, Clauser C, Cortellini P, Tinti C, Vincenzi G, Pagliaro U. Guided tissue regeneration versus mucogingival surgery in the treatment of human buccal recessions. A 4-year follow-up study. J Periodontol. 1996;67:1216-1223.
Fernández-Jiménez A, García-De-La-Fuente AM, Estefanía-Fresco R, Marichalar-Mendia X, Aguirre-Urizar JM, Aguirre-Zorzano LA. Complete root coverage in the treatment of Miller class III or RT2 gingival recessions: a systematic review and meta-analysis. BMC Oral Health. 2021 Mar 22;21(1):145. doi: 10.1186/s12903-021-01494-3. Erratum in: BMC Oral Health. 2021 Apr 26;21(1):213. PMID: 33752657; PMCID: PMC7986294.
Imber JC, Kasaj A. Treatment of Gingival Recession: When and How? Int Dent J. 2021 Jun;71(3):178-187. doi: 10.1111/idj.12617. Epub 2021 Jan 29. PMID: 34024328; PMCID: PMC9275303.
Zucchelli G, Tavelli L, McGuire MK, Rasperini G, Feinberg SE, Wang HL, Giannobile WV. Autogenous soft tissue grafting for periodontal and peri-implant plastic surgical reconstruction. J Periodontol. 2020 Jan;91(1):9-16. doi: 10.1002/JPER.19-0350. Epub 2019 Oct 6. PMID: 31461778.
Zucchelli G, Mounssif I. Periodontal plastic surgery. Periodontol 2000. 2015 Jun;68(1):333-68. doi: 10.1111/prd.12059. PMID: 25867992.
Barootchi S, Tavelli L, Zucchelli G, Giannobile WV, Wang HL. Gingival phenotype modification therapies on natural teeth: A network meta-analysis. J Periodontol. 2020 Nov;91(11):1386-1399. doi: 10.1002/JPER.19-0715. Epub 2020 Jun 12. PMID: 32392401.
Cairo F, Nieri M, Pagliaro U. Efficacy of periodontal plastic surgery procedures in the treatment of localized facial gingival recessions. A systematic review. J Clin Periodontol. 2014 Apr;41 Suppl 15:S44-62. doi: 10.1111/jcpe.12182. PMID: 24641000.

Declaración final

El equipo de investigadores, representado por el Patrocinador y el Investigador Principal del proyecto, de forma libre y voluntaria declaran lo siguiente:

-Que el contenido, la autoría y la responsabilidad sobre los resultados del estudio corresponden al Patrocinador y al Investigador Principal y que se exonera al Ministerio de Salud Pública de cualquier acción legal que se derive por esta causa.

-Que el proyecto descrito en este documento es una obra original, cuyos autores forman parte del equipo de investigadores y que por lo tanto se asume la completa responsabilidad legal en el caso de que un tercero alegue la titularidad de los derechos intelectuales del proyecto; Así como se exonera al Ministerio de Salud Pública de cualquier acción legal que se derive por esta causa.

-Que el presente proyecto no causa perjuicio alguno a los sujetos participantes en la investigación y al ambiente y no transgrede normativa legal o norma ética alguna, y que en el caso de que la investigación requiera de permisos de otras instituciones ajenas al Ministerio de Salud Pública, previo a su ejecución, el Patrocinador/Investigador Principal remitirán una copia certificada de los mismos al Ministerio de Salud Pública del Ecuador.

-Que velarán por el cumplimiento de la presente investigación en los términos que se aprobó tanto por el Comité de Ética de Investigación en Seres Humanos como por el Ministerio de Salud Pública.

-Que se enviará un informe final de la investigación con los resultados obtenidos al Ministerio de Salud Pública.

Lugar: *Ciudad - Provincia*
Fecha:

ANTONIO GABRIEL LANATA FLORES Digitally signed by ANTONIO GABRIEL LANATA FLORES

Antonio Gabriel Lanata Flores

Nombres y Apellidos investigador principal

Firma investigador principal (obligatorio)

0916524820

Cédula de ciudadanía o pasaporte Investigador principal (obligatoria)

Nombres y Apellidos Patrocinador

Firma Patrocinador (obligatorio)

Cédula de ciudadanía o pasaporte Patrocinador (obligatoria)
