# Supplementary material for: Comparison of bone augmentation techniques in the mandibular posterior region: A randomized clinical trial protocol
Source: PLoS One. 2026 Oct 29;21(10):e0337304. doi: 10.1371/journal.pone.0337304 (PMC13718928; doi:10.1371/journal.pone.0337304)
Supplement: Supplementary file 2 [file pone.0337304.s005.pdf]

Editorial Requirement 1 – Formatting and Style
We have carefully revised the manuscript to ensure full compliance with PLOS ONE formatting and style requirements, including file naming conventions, title page structure, abstract formatting, and reference style. The manuscript has been aligned with the PLOS ONE formatting templates provided.

Editorial Requirement 2 – Competing Interests
We confirm that the Competing Interests statement has been completed on the online submission form as follows:
The authors have declared that no competing interests exist.
This statement has also been included in the cover letter, as requested.

Editorial Requirement 3 – Ethics-Approved Study Protocol
We have uploaded the complete ethics-approved study protocol as a Supporting Information file in its original language. An English translation has also been provided, in accordance with PLOS ONE guidelines for clinical trial protocols.

Editorial Requirement 4 – Suggested Citations
We carefully reviewed all reviewer-suggested references. Relevant publications have been incorporated where appropriate. No citations were added unless they were directly relevant to the study design or methodological framework.

Response to Reviewer #1 – Comment 1
We thank the reviewer for this important comment. We agree that a fixed rotating sequence could introduce allocation predictability. The manuscript has been revised to specify that allocation will be performed using a computer-generated randomization sequence created via Random.org, with variable block sizes and allocation concealment ensured through sequentially numbered, opaque, sealed envelopes (SNOSE), prepared by an independent researcher.
The description of rotating allocation has been removed entirely.

Reviewer #1 – Comment 2 (Sample-size description is inconsistent and must be reconciled)
We appreciate the reviewer's careful reading. All inconsistencies have been corrected. The final planned sample size is 78 participants (26 per group), as stated in the Abstract, Participants section, and Sample Size section. The unit of analysis for the primary endpoint is the participant. When two sites are treated, a participant-level summary (mean horizontal gain) will be used.
The sample-size calculation is based on two pre-specified pairwise superiority comparisons (Group A vs B; Group A vs C), assuming:

- Pooled SD ≈ 1.12 mm,
- Clinically relevant difference = 1.0 mm,
- 80% power,
- Family-wise $\alpha = 0.05$ with multiplicity adjustment.

All sections have been fully aligned.

Reviewer #1 – Comment 3 (Primary endpoint and primary model need explicit designation)
We have clarified that:

- The single confirmatory primary endpoint is horizontal bone gain (mm) at 6 months.

- The primary analysis model is ANCOVA with treatment group as fixed factor and baseline ridge width as covariate.
- The framework is superiority.
- Only two primary contrasts (A vs B; A vs C) are confirmatory.
- All longitudinal and other outcomes are explicitly labeled as secondary or exploratory.

All ambiguity regarding multiple primary time points has been removed.

Reviewer #1 – Comment 4 (Non-inferiority framework insufficiently aligned with planned analyses)
We thank the reviewer for identifying this issue. The study is no longer framed as a non-inferiority trial. The design has been revised to a superiority framework. All references to non-inferiority margins have been removed. The statistical plan now uses multiplicity-adjusted superiority testing (Holm procedure) for the two pre-specified primary comparisons.

Reviewer #1 – Comment 5 (Missing-data strategy requires specification)
We have expanded the missing-data section to fully specify:

- Threshold for imputation (>5% missingness),
- 20 imputations using MICE,
- Variables included in the imputation model (group, baseline ridge width, age, sex, smoking, post-baseline ridge width values, complications),
- Pooling via Rubin's rules,
- Sensitivity analyses:
    - complete-case analysis,
    - delta-adjusted pattern-mixture analysis.

This has been fully detailed in the Statistical Analysis section.

Reviewer #1 – Comment 6 (Blinding description should be clarified)
The manuscript has been revised to state that this is a statistician-blinded, parallel-group trial.
We clarify that:

- The surgeon cannot be blinded.
- Participants cannot be fully blinded due to autogenous harvesting.
- The statistician remains blinded until completion of primary analysis.

All references to "double-blind" have been removed.

Reviewer #1 – Comment 7 (Clustering must be consistently addressed across analyses)
We have clarified that:

- The primary analysis is participant-level.
- When two sites are present, a participant-level summary is used.
- For site-level outcomes, mixed-effects models including Participant as a random effect will be applied.
- Longitudinal models include Participant (and Site nested within Participant when applicable) as random effects.

This has been explicitly described.

Reviewer #1 – Comment 8 (Multiplicity across secondary outcomes)
We now explicitly state that:

- Only the two primary contrasts are confirmatory.
- All secondary and exploratory outcomes will not undergo formal multiplicity correction.
- Results will be interpreted using effect sizes and confidence intervals rather than dichotomous significance testing.

Reviewer #2 – Comment 1 (Design inconsistency)
Thank you for identifying this important inconsistency. The study design has been clarified throughout the manuscript. The trial is a randomized, statistician-blinded, parallel-group clinical trial. All references to a split-mouth or double-blind design have been removed to ensure full conceptual coherence between the Introduction and Methods sections. The Introduction, Abstract, and Study Design sections have been revised to consistently reflect the true parallel-group allocation structure.

Reviewer #2 – Comment 2 (Sample size inconsistencies)
All inconsistencies have been corrected. The final planned sample size is 78 participants (26 per group), consistently reported across Abstract, Participants, and Sample Size sections.

Reviewer #2 – Comment 3 (Inconsistency between non-inferiority objective and statistical analysis)
The study is now explicitly framed as a superiority trial. All references to non-inferiority have been removed, and the statistical analysis plan has been fully aligned with this framework.

Reviewer #2 – Comment 4 (Randomization method is not truly random)
Thank you for this important methodological observation. The previous fixed rotating allocation sequence has been removed. The revised protocol now employs a computer-generated randomization sequence created through the web-based platform Random.org, using block randomization with variable block sizes to ensure both balance and unpredictability.
The allocation sequence will be generated by an independent researcher not involved in recruitment, surgery, or outcome assessment. Allocation concealment will be maintained using sequentially numbered, opaque, sealed envelopes (SNOSE), opened only after eligibility confirmation and informed consent. These revisions eliminate allocation predictability and ensure true randomization with appropriate concealment. The manuscript has been updated accordingly.

Reviewer #2 – Comment (Blinding)
Thank you for this important clarification. The study is not double-blind, and the manuscript has been revised to accurately describe the blinding structure. Due to the surgical nature of the interventions and the need for donor-site harvesting in the autogenous group, blinding of the surgeon and participants is not feasible. The trial is now clearly described as statistician-blinded, meaning that the statistician performing the primary analysis will remain blinded to group allocation through coded identifiers. All terminology suggesting double blinding has been removed to ensure transparency and methodological accuracy.

Reviewer #2 – Comment 5 (Primary outcome time point)

We thank the reviewer for identifying this inconsistency. The protocol has been revised to clearly define a single primary endpoint: horizontal bone gain at 6 months postoperatively. All sections (Abstract, Sample Size, and Statistical Analysis) have been aligned accordingly. Measurements at 3 and 12 months are now explicitly described as secondary exploratory outcomes.

Reviewer #2 – Comment 6 (Use of Hounsfield Units)
The manuscript has been revised to remove the term “Hounsfield Units (HU)” when referring to CBCT-derived measurements. Radiographic density will now be reported as device-specific gray values under standardized acquisition parameters.

Reviewer #2 – Comment 7 (Baseline ridge)
The manuscript has been revised to explicitly state that baseline ridge width will be measured from the preoperative CBCT using the same standardized reference lines (5, 7, and 11 mm from the crest) and identical measurement protocol as postoperative assessments.

Reviewer #2 – Comment 8 (Postoperative inflammation outcome)
We have clarified that linear facial measurements are exploratory and complemented by 3D structured-light facial scanning to improve objectivity and reproducibility.

Reviewer #2 – Comment 9 (Exclusion criteria)
We thank the reviewer for this observation. We agree that hormonal contraceptive use is not a standard exclusion criterion in bone-augmentation trials. After reconsideration, this criterion has been removed from the protocol. Only pregnancy and lactation are now listed as exclusion criteria due to surgical and radiographic considerations.

Reviewer #2 – Comment 10 (Smoking exclusion)
We clarified that:

- Only smokers with >5 cigarettes/day are excluded.
- Former smokers are eligible.
- Smoking status is assessed via structured self-report without biochemical verification.

This clarification has been incorporated into the Eligibility Criteria section.

Reviewer #2 – Comment 11 (Ethics committee)
The institutional name has been standardized throughout the manuscript to ensure consistency.

Reviewer #2 – Comment 12 (Soft-tissue outcomes)
We have clarified the role of intraoral scans in the revised manuscript. Soft-tissue contour changes derived from intraoral surface scans are now explicitly defined as exploratory secondary outcomes. These measurements will not be used for confirmatory hypothesis testing but will provide descriptive quantitative information regarding external volumetric and morphological remodeling. The Statistical Analysis section has been updated accordingly.
