# Supplementary material for: Comparison of bone augmentation techniques in the mandibular posterior region: A randomized clinical trial protocol
Source: PLoS One. 2026 Oct 29;21(10):e0337304. doi: 10.1371/journal.pone.0337304 (PMC13718928; doi:10.1371/journal.pone.0337304)
Supplement: Supplementary file 3 [file pone.0337304.s006.pdf]

Manuscript Title:
Comparison of Bone Augmentation Techniques in the Mandibular Posterior Region: A Randomized Clinical Trial Protocol

Manuscript ID: PONE-D-25-58795R1
We sincerely thank the Editor and Reviewers for their valuable comments and suggestions. We have carefully revised the manuscript and addressed each point as detailed below.

Reviewer #1 – Comment 1
Randomization description remains contradictory. The manuscript includes both a computer-generated block randomization/SNOSE description and (elsewhere) the prior rotating A→B→C schedule text. Please remove the rotating-schedule language entirely and ensure the final clean manuscript contains only one allocation method, consistently described throughout.
Response:
We thank the reviewer for pointing this out. We removed the previous rotating A→B→C allocation text from the manuscript. The randomization method is now described consistently as computer-generated block randomization with a 1:1:1 allocation ratio, using sequentially numbered, opaque, sealed envelopes (SNOSE) for allocation concealment.
We also clarified that, when a participant has two eligible posterior mandibular sites, both sites will receive the same allocated intervention. This is consistent with the parallel-group design and the participant-level primary analysis.
Location of changes:

- Materials and Methods section, "Study groups and interventions" subsection.
- Materials and Methods section, "Randomization and blinding" subsection.

Reviewer #1 – Comment 2
Sample-size section is internally inconsistent regarding superiority vs non-inferiority. The text states the trial follows a superiority framework with no non-inferiority margin, but the same section still contains a non-inferiority sample-size paragraph (including one-sided alpha and hierarchical testing). Please fully align the sample size rationale with the final chosen framework (superiority), specify the confirmatory primary comparisons (e.g., A vs B; A vs C), and state clearly how multiplicity will be controlled for these primary contrasts. Also specify the assumed attrition rate (or expected loss to follow-up) used to justify the final n=78.
Response:
We thank the reviewer for this important observation. The sample-size section has been revised to fully align the protocol with the final superiority framework. All non-inferiority language, including any reference to a non-inferiority margin, one-sided alpha level, or hierarchical non-inferiority testing, has been removed.
We now specify that the single confirmatory primary endpoint is participant-level horizontal bone gain at 6 months. The two confirmatory primary comparisons are Group A (autogenous cortical shell) versus Group B (xenogeneic cortical shell), and Group A versus Group C (allogeneic cortical shell). The Group B versus Group C comparison will be considered secondary and exploratory. Multiplicity across the two confirmatory primary contrasts will be controlled using the Holm–Bonferroni procedure, maintaining a two-sided family-wise alpha level of 0.05.
We also clarified that the final sample size of 78 participants includes an anticipated 10% loss to follow-up before the 6-month CBCT assessment, while preserving equal allocation across the three groups.
Location of changes:

- Materials and Methods section, "Sample size" subsection.
- Materials and Methods section, "Statistical analysis – Primary and secondary endpoints" subsection.

Reviewer #1 – Comment 3
Statistical analysis section contains duplicated/contradictory methods and embedded reviewer text. Although the primary analysis is described as ANCOVA at 6 months, the same section also retains one-way ANOVA with Tukey post-hoc tests and repeated/duplicated LMM text, and it includes a pasted paragraph stating "Missing-data strategy needs further specification…". Please remove the embedded reviewer paragraph and harmonize the analysis plan to one coherent primary approach (ANCOVA for the confirmatory endpoint at 6 months, with explicitly pre-specified primary contrasts and multiplicity adjustment), with secondary/exploratory models clearly labeled.

Response:
We thank the reviewer for noting this issue. We revised the statistical analysis section to remove duplicated and contradictory text while keeping the primary analysis consistent with the planned ANCOVA framework.
The primary endpoint is now described as participant-level horizontal bone gain at 6 months, analyzed using ANCOVA with treatment group as the fixed factor and baseline ridge width as a covariate. We also clarified that the two confirmatory primary contrasts will be Group A versus Group B and Group A versus Group C, with Holm–Bonferroni adjustment for multiplicity.
The reviewer comment that had inadvertently remained in the manuscript was removed. Secondary longitudinal, morphometric, categorical, and soft-tissue analyses are now described as secondary or exploratory.
Location of changes:

- Materials and Methods section, “Statistical analysis – Primary and secondary endpoints” subsection.

Reviewer #1 – Comment 4
Missing-data plan is still not actually specified in the manuscript text. The protocol currently still states only that MI will be used if missingness exceeds 10%, without specifying number of imputations, imputation model variables, pooling (Rubin’s rules), or sensitivity analyses; moreover, the manuscript contains the pasted reviewer paragraph noting these omissions. Please replace this with a complete pre-specified approach (threshold for MI, number of imputations, method such as MICE, variables included, pooling rules, and at least one sensitivity analysis such as complete-case and/or pattern-mixture/delta adjustment).
Response:
We thank the reviewer for this important comment. We added a dedicated missing-data section to the statistical analysis plan.
The revised text now specifies that the extent and reasons for missing data will be described by treatment group. If missing data for the primary endpoint exceed 10%, multiple imputation will be performed using multivariate imputation by chained equations (MICE), with 20 imputed datasets. The imputation model will include treatment group, baseline ridge width, age, sex, smoking status, number of treated sites, and available horizontal bone gain measurements at 3, 6, and 12 months. Estimates and standard errors from the imputed datasets will be combined according to Rubin’s rules, and the corresponding reference was added to the reference list. We also added complete-case and delta-adjusted sensitivity analyses to assess the robustness of the primary endpoint results.
Location of changes:

- Materials and Methods section, “Statistical analysis – Missing data” subsection.
- References section.

Reviewer #1 – Comment 5
Blinding statements remain inconsistent across document components. Parts of the submission still describe the trial as “double-blind,” while the revised abstract describes “statistician-blinded,” and the methods acknowledge image processing is not masked. Please ensure “double-blind” is removed everywhere and provide a consistent statement of who is blinded (statistician; ideally outcome assessors if feasible) and what safeguards will mitigate detection bias when image processing cannot be masked (e.g., pre-specified segmentation rules, calibration, blinded duplicate measurement, and reporting inter-/intra-rater reliability).
Response:
We thank the reviewer for pointing out this inconsistency. We revised the manuscript to remove references to “double-blind” and other unsupported blinding descriptions.
The trial is now described consistently as a randomized, statistician-blinded, parallel-group clinical trial. We clarified that the statistician responsible for the primary analysis will remain blinded to treatment allocation through coded group identifiers until completion of the primary analysis. We also state that blinding of the operating surgeon and participants is not feasible because of the surgical nature of the interventions and the need for autogenous bone harvesting in Group A.
We further clarified how detection bias will be minimized during outcome assessment. The revised manuscript now specifies the use of standardized CBCT segmentation and surface-registration protocols, pre-specified segmentation rules, examiner calibration, duplicate measurements, and reporting of intra- and inter-examiner reliability using intraclass correlation coefficients.
Location of changes:

- Abstract.
- Materials and Methods section, “Randomization and blinding” subsection.
- Materials and Methods section, “Volumetric and morphometric analysis” subsection.

Reviewer #3 – Comment 1
The sample size calculation assumes a common SD of 1.2 mm, but the authors do not justify this value. While references 13 and 14 are cited, the specific SD values from these studies are not reported. The authors should state the actual SD values from the cited studies (e.g., "Lin et al. reported a pooled SD of X mm")
Response:
We thank the reviewer for this helpful comment. The sample-size section has been revised to report the specific standard deviation values from the studies used to support the assumed common SD of 1.2 mm.
We now state that Lin et al. reported horizontal ridge resorption at 1 mm from the crest of 1.29 ± 1.37 mm, 1.07 ± 1.07 mm, and 1.54 ± 1.33 mm across the three study groups at 6 months, corresponding to an approximate pooled SD of 1.26 mm. We also state that Kim et al. reported horizontal width changes at 3 mm from the crest of −0.37 ± 1.31 mm, 0.10 ± 0.97 mm, and 0.72 ± 0.80 mm across groups, corresponding to an approximate pooled SD of 1.05 mm.
Based on these values, the assumed common SD of 1.2 mm was retained as a reasonable estimate for the present trial.
Location of changes:

- Materials and Methods section, “Sample size” subsection.

Reviewer #3 – Comment 2
The primary endpoint is horizontal bone gain at 6 months, but the sample size calculation does not explicitly state that the 6-month time point was used for power calculations. Clarify that the 6-month assessment was used for the primary analysis.
Response:
We thank the reviewer for this comment. We clarified in the sample-size section that the primary endpoint used for the sample-size calculation is participant-level horizontal bone gain at 6 months, measured by CBCT. We also specified that the 6-month assessment was selected a priori as the definitive confirmatory time point for hypothesis testing.
Location of changes:

- Materials and Methods section, “Sample size” subsection.

Reviewer #3 – Major Concern 3
The authors removed hormonal contraceptive use from exclusion criteria, which is appropriate. However, they should consider whether this is relevant for women of childbearing age and whether pregnancy testing is performed before surgery.
Response:
We thank the reviewer for this comment. We agree that hormonal contraceptive use should not be an exclusion criterion by itself. We clarified that, for women of childbearing age, possible pregnancy will be assessed by self-report before surgery. If pregnancy status is uncertain, the participant may either be excluded from the study or undergo a urine pregnancy test before surgical intervention. Pregnant or lactating women remain excluded from participation.
Location of changes:

- Materials and Methods section, “Participants” subsection.

Reviewer #3 – Major Concern 4
The study is registered at ClinicalTrials.gov (NCT06661447). The authors should confirm that the registration is complete and that all protocol details (primary outcome, sample size) match.
Response:
We thank the reviewer for this comment. We reviewed the ClinicalTrials.gov registration and updated the relevant protocol details to match the revised manuscript, including the primary outcome, the 6-month confirmatory time point, the planned sample size of 78 participants, and the parallel-group allocation.
The ClinicalTrials.gov registration is complete and now matches the revised protocol.
Location of changes:

- ClinicalTrials.gov registration record NCT06661447.

Reviewer #3 – Major Concern 5
The surface registration workflow uses CloudCompare (open-source software). This is acceptable, but the authors should specify the software version and whether any custom scripts were used for batch processing.
Response:
We thank the reviewer for this comment. We revised the surface-registration workflow description to specify the CloudCompare version used for the analysis. We also clarified that no custom scripts will be used for batch processing and that registration and measurement procedures will be performed using the standard graphical user interface tools available in CloudCompare.
Location of changes:

- Materials and Methods section, "Volumetric and morphometric analysis – Surface registration and quantitative analysis" subsection.

Reviewer #3 – Major Concern 6
The statistician-blinded design is appropriate. However, the authors should clarify who will break the blind if needed, for example in case of serious adverse events.
Response:
We thank the reviewer for this comment. We clarified the unblinding procedure in the Randomization and blinding section. If unblinding is required for participant safety, such as in the event of a serious adverse event, the principal investigator will authorize and document the unblinding process.
Location of changes:

- Materials and Methods section, "Randomization and blinding" subsection.

Reviewer #3 – Minor Concern 1
Abstract – the phrase "statistician-blinded" appears twice. Remove duplicate.
Response:
We thank the reviewer for noting this. We reviewed the Abstract and removed the duplicate wording. The trial is now described once as a randomized, statistician-blinded, parallel-group clinical trial.
Location of changes:

- Abstract.

Reviewer #3 – Minor Concern 2
Abstract – the primary outcome is stated as "horizontal bone gain (mm) at 6 months". This is correct, but the introduction of the abstract also mentions 3, 6, and 12 months – clarify that only 6 months is confirmatory.
Response:
We thank the reviewer for this comment. We clarified in the Abstract that horizontal bone gain at 6 months is the single primary endpoint. The 3- and 12-month CBCT assessments are now described as secondary longitudinal outcomes.
Location of changes:

- Abstract.

Reviewer #3 – Minor Concern 3
Methods – smoking exclusion: The authors state that "only current heavy smokers (>5 cigarettes/day) will be excluded" but do not specify how smoking status will be verified. Self-report is acceptable, but this should be stated.
Response:
We thank the reviewer for this observation. We clarified that smoking status will be assessed at baseline by structured self-report and categorized as current heavy smokers, light smokers, former smokers, or never smokers. Smoking history and vaping use will also be recorded for descriptive and exploratory analyses.
Location of changes:

- Materials and Methods section, "Participants" subsection.

Reviewer #3 – Minor Concern 4

Methods – postoperative facial swelling: The measurement from the oral commissure to the tragus is described. The authors should specify whether the same examiner performs all measurements to ensure reproducibility.
Response:
We thank the reviewer for this comment. We clarified that postoperative facial swelling measurements will be performed twice by the same calibrated examiner, and the mean value will be recorded to improve reproducibility.
Location of changes:

- Materials and Methods section, “Surgical procedure” subsection.

Reviewer #3 – Minor Concern 5
Results – expected results: The authors anticipate that xenogeneic and allogeneic laminae will achieve comparable outcomes to autogenous plates. The authors should consider whether a “non-inferiority” framework would be more appropriate for comparing prefabricated materials to the gold standard (autogenous), but they have already addressed this and chosen superiority.
Response:
We thank the reviewer for this thoughtful comment. We considered this point carefully and have retained a superiority framework for the present protocol. To avoid ambiguity, the sample-size and statistical-analysis sections were revised to remove non-inferiority language and to state the confirmatory primary comparisons and multiplicity adjustment clearly.
Location of changes:

- Materials and Methods section, “Sample size” and “Statistical analysis” subsections.

Reviewer #3 – Minor Concern 7
The authors should add a short statement on the expected clinical implications of the findings.
Response:
We thank the reviewer for this suggestion. We added a short statement on the expected clinical implications of the study findings. Specifically, we note that if prefabricated xenogeneic or allogeneic laminae show comparable clinical and volumetric performance to autogenous cortical shells, they may help reduce donor-site morbidity and surgical time and support their use as alternative treatment options for posterior mandibular ridge augmentation.
Location of changes:

- Expected Results and Discussion section.
