# Supplementary material for: Comparison of bone augmentation techniques in the mandibular posterior region: A randomized clinical trial protocol
Source: PLoS One. 2026 Oct 29;21(10):e0337304. doi: 10.1371/journal.pone.0337304 (PMC13718928; doi:10.1371/journal.pone.0337304)
Supplement: Supplementary file 4 [file pone.0337304.s007.pdf]

Manuscript Title:
Comparison of Bone Augmentation Techniques in the Mandibular Posterior Region: A Randomized Clinical Trial Protocol

Manuscript ID: PONE-D-25-58795R2
We sincerely thank the Editor and Reviewers for their valuable comments and suggestions. We have carefully revised the manuscript and addressed each point as detailed below.

Reviewer #1 – Comment 1
Superiority framework remains inconsistent with the scientific objective.
We thank the reviewer for this important clarification. We retained the superiority framework and revised the manuscript to ensure that the objectives, hypotheses, expected results, and clinical implications are aligned with this framework.
Specifically, we removed wording suggesting that the trial could establish "comparable" or "similar" performance between prefabricated laminae and autogenous cortical shells. The Introduction now states that the study aims to evaluate differences in clinical, radiographic, and volumetric outcomes among the three treatment groups. The Expected Results and Discussion section was also revised so that the planned interpretation focuses on differences in horizontal bone gain, dimensional stability, surgical duration, postoperative morbidity, and volumetric stability, rather than equivalence or non-inferiority.

Location of changes:
- Introduction section.
- Expected results section.
- Discussion section.

Reviewer #1 – Comment 2
Sample-size calculation remains insufficiently reproducible.
We thank the reviewer for this detailed comment. The sample-size section was revised, and Supplementary File 1 was added to provide the complete G*Power calculation.

The revised manuscript now states that the sample size was calculated a priori using G*Power version 3.1.9.7. The calculation was based on the two prespecified confirmatory comparisons: Group A versus Group B and Group A versus Group C. For sample-size planning, a conservative two-sided alpha level of 0.025 per comparison was used; the final confirmatory analyses will control the family-wise type I error using the Holm procedure.

We also specified the assumed alternative used for planning: a clinically relevant between-group difference of 1.0 mm in horizontal bone gain at 6 months, with a common standard deviation of approximately 1.12 mm, corresponding to Cohen's d = 0.893. This standard deviation was derived from a previous CBCT-based randomized clinical trial reporting 6-month variability in horizontal ridge augmentation. Under these assumptions, 26 evaluable participants per group are required for each pairwise comparison, with an achieved power of approximately 81.5%.

To account for approximately 10% attrition, the enrollment target was increased to 29 participants per group, resulting in a total planned sample size of 87 participants. We clarified that the reported power refers to each prespecified confirmatory contrast, not to joint rejection of both contrasts. We also clarified that no expected efficiency gain from ANCOVA adjustment was incorporated into the sample-size calculation; therefore, no baseline-to-follow-up correlation or variance-reduction factor was assumed.

The previous "80–90% power" statement in the Abstract was corrected. We also added a sensitivity analysis across plausible standard deviation values in Supplementary File 1 to address the transportability of the variance estimate from a related but not identical CBCT-based randomized trial.

Location of changes:
- Abstract.
- Materials and Methods section, "Participants" subsection.
- Materials and Methods section, "Sample size" subsection.
- Supplementary File 1.

Reviewer #1 – Comment 3

Missing-data plan remains partly data-dependent and incomplete.
We thank the reviewer for this helpful comment. We revised the missing-data plan so that the primary approach no longer depends on an arbitrary missingness threshold. The revised manuscript now specifies that, if any 6-month primary-outcome data are missing, multiple imputation by chained equations will be used under a missing-at-random assumption.

We clarified that the primary analysis will follow the intention-to-treat principle and will include all randomized participants according to their assigned treatment group. The imputation procedure is now prespecified with 20 imputed datasets and an imputation model including treatment group, baseline ridge width, age, sex, smoking status, available horizontal ridge-width measurements at other follow-up time points, and clinically relevant variables associated with outcome or loss to follow-up, including postoperative complications when available. Treatment-effect estimates and standard errors will be combined using Rubin's rules, and the corresponding reference was added.

We also specified the delta-adjusted sensitivity analysis in greater detail. Because the primary superiority hypothesis favors Group A, the MNAR sensitivity analysis will impose assumptions unfavorable to Group A by progressively reducing imputed 6-month horizontal bone-gain values in that group by prespecified deltas of −0.25, −0.50, −0.75, and −1.00 mm. A tipping-point analysis will also be performed using 0.10-mm increments until the conclusion for each primary contrast changes.

Finally, we clarified that secondary longitudinal mixed-effects models will include all available repeated observations under the missing-at-random assumption, without last-observation-carried-forward or single-value imputation.

Location of changes:
- Materials and Methods section, "Missing data" subsection.
- References.

Reviewer #1 – Comment 4
Secondary-outcome analyses remain under-specified.
We thank the reviewer for this comment. We expanded the statistical analysis plan to provide an outcome-by-outcome analysis strategy for the secondary outcomes.

The revised manuscript now specifies the analysis unit, model, treatment effect, and confidence interval reporting for longitudinal horizontal bone gain, volumetric change, morphometric parameters, relative CBCT gray values, surgical duration, repeated postoperative facial-swelling measurements, soft-tissue contour changes, and postoperative complications.

For longitudinal horizontal bone gain, we specified that the model will include group, time, and group-by-time interaction as fixed effects, baseline ridge width as a covariate, and participant as a random intercept. Time will be treated as a categorical factor, and an unstructured covariance matrix will be used for within-participant repeated measurements. We also clarified that model assumptions will be assessed using residual diagnostics rather than normality testing of raw outcome data.

For surgical duration, we clarified that time will be recorded from administration of local anesthesia to the final suture for each surgical session. If two sites are treated during the same session, total surgical duration for that session will constitute the participant-level observation.

Location of changes:
- Materials and Methods section, "Statistical analysis: Secondary outcome analyses" subsection.

Reviewer #1 – Comment 5
Outcome-assessor masking and measurement reliability require clarification.
We thank the reviewer for this comment. We revised the Randomization and blinding section to clarify the masking procedures for outcome assessment.

The revised manuscript now states that the statistician responsible for the primary analysis will remain blinded to treatment allocation through coded group identifiers. We also clarified that the operating surgeon and participants cannot be blinded because of the surgical nature of the interventions and the need for autogenous bone harvesting in one group.

For outcome assessment, we clarified that CBCT datasets and intraoral scan files will be coded before measurement so that outcome examiners do not have access to participant identifiers or treatment-

group labels during linear and volumetric measurements. We also acknowledge that complete masking of image-processing operators may not be feasible because some graft materials may have recognizable radiographic or morphological features.

To reduce detection bias, the revised manuscript specifies standardized scan analysis, prespecified segmentation and surface-registration rules, duplicate independent measurements by two calibrated examiners, and reporting of intra- and inter-examiner reliability using intraclass correlation coefficients. We also added that, if the prespecified reliability criterion is not achieved, examiners will repeat calibration and the measurement protocol will be reviewed before final outcome measurements are completed.

Location of changes:

- Materials and Methods section, "CBCT-based segmentation and linear measurements" subsection.
- Materials and Methods section, "Randomization and blinding" subsection.

Reviewer #1 – Comment 6
Data-availability plan.
We thank the reviewer for this comment. We revised the data-availability statement so that access to study data does not depend solely on request to the corresponding author.

The revised manuscript now states that the de-identified participant-level dataset underlying the reported results, the data dictionary, metadata, and statistical code will be deposited in a public repository in accordance with PLOS ONE data policies. We also clarified that complete CBCT/DICOM and intraoral-scan imaging files will not be publicly released because they may contain potentially identifiable anatomical information. Access to these imaging files, when scientifically justified and ethically permissible, will be evaluated through an institutional data-access process rather than depending solely on the discretion of the corresponding author.

Location of changes:

- Materials and Methods section, "Data management and confidentiality" subsection.

Reviewer #1 – Comment 7
Final consistency checks.
We thank the reviewer for this final consistency check. We reviewed the manuscript to ensure that the superiority framework is used consistently throughout the text. The previous "80–90% power" wording was removed, and statements suggesting "comparable" or "similar" treatment performance were revised so that the manuscript now refers to testing and estimating differences among treatment groups.

We also corrected the sample-size wording throughout the manuscript so that the planned enrollment is consistently reported as 87 participants, with 29 participants per group, allowing for approximately 10% attrition and targeting 26 evaluable participants per group.

We added a brief statement in the Trial Registration section indicating that the registry record will be updated before recruitment begins. Any required protocol amendments will also be submitted for ethics review and approval before implementation.

Location of changes:

- Abstract.
- Materials and Methods section, "Participants" subsection.
- Trial Registration section.
