# Supplementary material for: Comparison of bone augmentation techniques in the mandibular posterior region: A randomized clinical trial protocol
Source: PLoS One. 2026 Oct 29;21(10):e0337304. doi: 10.1371/journal.pone.0337304 (PMC13718928; doi:10.1371/journal.pone.0337304)
Supplement: Supplementary file 5 [file pone.0337304.s001.docx]

**Supplementary File 1. Sample Size Calculation (G*Power 3.1.9.7)**

The required sample size for the primary endpoint, horizontal bone gain at 6 months, was calculated a priori using G*Power version 3.1.9.7 (Heinrich-Heine-Universität Düsseldorf, Germany).

**Statistical framework**

The trial follows a superiority framework with two prespecified confirmatory pairwise comparisons:

- Group A (autogenous cortical shell) vs Group B (xenogeneic cortical shell)
- Group A (autogenous cortical shell) vs Group C (allogeneic cortical shell)

The final confirmatory analyses will control the family-wise type I error across these two primary contrasts using the Holm step-down procedure.

Because the Holm procedure is data-dependent and cannot be represented by a single fixed alpha level in an a priori G*Power calculation, a conservative Bonferroni-adjusted two-sided significance level of α = 0.025 per comparison was used for sample-size planning. This corresponds to an overall family-wise α of 0.05 across the two primary comparisons.

The power reported in this calculation refers to each individual confirmatory contrast evaluated at the conservative two-sided α = 0.025 used for planning.

**Parameters used in G*Power**

**Test family:** t tests
**Statistical test:** Means – Difference between two independent means (two groups)
**Type of power analysis:** A priori (compute required sample size given α, power, and effect size)

**Input parameters**

- Tail(s): Two
- Effect size (Cohen’s d): 0.893
- α error probability: 0.025
- Power (1 − β): 0.80
- Allocation ratio (N2/N1): 1

**Effect size justification**

The calculation was based on a clinically relevant between-group difference (δ) of 1.0 mm in horizontal ridge-width gain at 6 months between the autogenous cortical shell group and each comparator group.

The assumed standard deviation was derived from a previously published randomized clinical trial using CBCT-based assessment of horizontal ridge augmentation at 6 months. That study reported standard deviations of 1.35 mm and 0.87 mm across the two treatment arms. The corresponding pooled standard deviation was approximately 1.12 mm (1).

Cohen’s d was calculated as:

d = δ / SD

d = 1.0 / 1.12 ≈ 0.893

The source study was considered clinically and methodologically relevant because it evaluated horizontal ridge augmentation using CBCT at a comparable follow-up period. However, the anatomical distribution and grafting techniques were not identical to those of the present trial. Therefore, the transportability of the variance estimate was examined through sensitivity analyses using alternative plausible SD values.

**G*Power results**

Under the prespecified assumptions, G*Power yielded:

- Required evaluable sample size per group: 26 participants
- Total sample size per pairwise comparison: 52 participants
- Degrees of freedom: 50
- Critical t value: 2.3109
- Achieved power: 0.815

Thus, 26 evaluable participants per treatment group are required to provide approximately 81.5% power for each prespecified confirmatory pairwise comparison under the conservative two-sided α = 0.025 used for planning.

Because the trial includes three parallel groups, a total of 78 evaluable participants would be required if complete primary-outcome data were available for all randomized participants.

**Adjustment for anticipated attrition**

To account for an anticipated attrition rate of approximately 10%, the recruitment target was inflated according to:

n enrolled = n evaluable / (1 − attrition proportion)

n enrolled = 26 / 0.90 = 28.89

Accordingly, 29 participants will be recruited per group, resulting in a total planned enrollment of: 29 × 3 = 87 participants.

This recruitment target is intended to retain approximately 26 evaluable participants per group after an anticipated 10% loss to follow-up.

**Relation to the primary ANCOVA model**

The primary analysis will use ANCOVA with treatment group as a fixed factor and baseline ridge width as a covariate.

However, no anticipated gain in efficiency from baseline covariate adjustment was incorporated into the sample-size calculation. Specifically, no assumption was made regarding the correlation between baseline ridge width and the 6-month outcome, and no variance-reduction factor was applied.

The sample-size calculation was therefore based on the conservative independent two-sample comparison described above rather than on an assumed ANCOVA efficiency gain.

**Sensitivity analysis for the assumed standard deviation**

Because the standard deviation was obtained from a related but not identical clinical setting, sensitivity analyses were conducted to evaluate the influence of alternative plausible SD values on the required sample size.

Assuming a 1.0-mm between-group difference, two-sided α = 0.025, 80% power, and equal allocation, the approximate required number of evaluable participants per group increases as the assumed SD increases:

| **Assumed SD** | **Approximate evaluable participants required per group** |
| --- | --- |
| 1.00 mm | 21 |
| 1.12 mm | 26 |
| 1.20 mm | 29 |
| 1.35 mm | 36 |
| 1.40 mm | 39 |

These sensitivity analyses demonstrate that statistical power is dependent on the transportability of the variance estimate from the reference trial. The prespecified primary calculation uses SD = 1.12 mm because it represents the pooled empirical variability reported in the most directly comparable randomized clinical trial identified for planning purposes.

**Interpretation**

The planned enrollment of 87 participants corresponds to 29 randomized participants per group and allows for approximately 10% attrition while targeting 26 evaluable participants per group.

Under the primary planning assumptions of a 1.0-mm mean difference, SD = 1.12 mm, and two-sided α = 0.025, the resulting evaluable sample provides approximately 81.5% power for each of the two prespecified confirmatory contrasts.

The final inferential analysis will use the Holm procedure to control the family-wise type I error across the two primary comparisons.

**Reference**

1. Mendoza-Azpur G, de la Fuente A, Chavez E, Valdivia E, Khouly I. Horizontal ridge augmentation with xenogeneic particulate graft alone or in combination with autogenous block graft: a randomized controlled clinical trial. Clin Implant Dent Relat Res. 2019;21(4):711–719. doi:10.1111/cid.12740.
