# Supplementary material for: Comparison of bone augmentation techniques in the mandibular posterior region: A randomized clinical trial protocol
Source: PLoS One. 2026 Oct 29;21(10):e0337304. doi: 10.1371/journal.pone.0337304 (PMC13718928; doi:10.1371/journal.pone.0337304)
Supplement: Peer review history

# Peer review: Decision Letter 0

**DOI:** 10.1371/journal.pone.0337304.r001

**Authors:** Carlos Alberto Viegas

2 Feb 2026

Dear Dr.  Lanata-Flores,

plosone@plos.org. When you're ready to submit your revision, log on to https://www.editorialmanager.com/pone/ and select the 'Submissions Needing Revision' folder to locate your manuscript file.

- A letter that responds to each point raised by the academic editor and reviewer(s). You should upload this letter as a separate file labeled 'Response to Reviewers'.
- A marked-up copy of your manuscript that highlights changes made to the original version. You should upload this as a separate file labeled 'Revised Manuscript with Track Changes'.
- An unmarked version of your revised paper without tracked changes. You should upload this as a separate file labeled 'Manuscript'.

If you would like to make changes to your financial disclosure, please include your updated statement in your cover letter. Guidelines for resubmitting your figure files are available below the reviewer comments at the end of this letter.

If applicable, we recommend that you deposit your laboratory protocols in protocols.io to enhance the reproducibility of your results. Protocols.io assigns your protocol its own identifier (DOI) so that it can be cited independently in the future. For instructions see: https://journals.plos.org/plosone/s/submission-guidelines#loc-laboratory-protocols. Additionally, PLOS ONE offers an option for publishing peer-reviewed Lab Protocol articles, which describe protocols hosted on protocols.io. Read more information on sharing protocols at https://plos.org/protocols?utm_medium=editorial-email&utm_source=authorletters&utm_campaign=protocols.

We look forward to receiving your revised manuscript.

Kind regards,

Carlos Alberto Antunes Viegas, DVM; MSc; PhD

Academic Editor

PLOS One

Journal Requirements:

When submitting your revision, we need you to address these additional requirements.

1.Please ensure that your manuscript meets PLOS ONE's style requirements, including those for file naming. The PLOS ONE style templates can be found at https://journals.plos.org/plosone/s/file?id=wjVg/PLOSOne_formatting_sample_main_body.pdf and https://journals.plos.org/plosone/s/file?id=ba62/PLOSOne_formatting_sample_title_authors_affiliations.pdf

2. Thank you for stating the following in your Competing Interests section:

“The authors declare that they have no competing interests.

They have no financial or personal relationships that could inappropriately influence (bias) this work.

The study received institutional support from Universidad de Especialidades Espíritu Santo (UEES), which had no role in the study design, data collection and analysis, decision to publish, or preparation of the manuscript.”

Please complete your Competing Interests on the online submission form to state any Competing Interests. If you have no competing interests, please state "The authors have declared that no competing interests exist.", as detailed online in our guide for authors at http://journals.plos.org/plosone/s/submit-now

This information should be included in your cover letter; we will change the online submission form on your behalf.

3. Please upload a copy of your study protocol that was approved by your ethics committee/IRB as a Supporting Information file. By the study protocol, we mean the complete and detailed plan for the conduct and analysis of the trial approved by the ethics committee/IRB. Please send this in the original language. If this is in a language other than English, please also provide a translation. [https://journals.plos.org/plosone/s/submission-guidelines#loc-guidelines-for-specific-study-types

4. If the reviewer comments include a recommendation to cite specific previously published works, please review and evaluate these publications to determine whether they are relevant and should be cited. There is no requirement to cite these works unless the editor has indicated otherwise.

[Note: HTML markup is below. Please do not edit.]

Reviewers' comments:

Reviewer's Responses to Questions

**Comments to the Author**

1. Does the manuscript provide a valid rationale for the proposed study, with clearly identified and justified research questions?

Reviewer #1: Yes

Reviewer #2: No

**********

2. Is the protocol technically sound and planned in a manner that will lead to a meaningful outcome and allow testing the stated hypotheses?

Reviewer #1: No

Reviewer #2: No

**********

3. Is the methodology feasible and described in sufficient detail to allow the work to be replicable?

Reviewer #1: No

Reviewer #2: No

**********

4. Have the authors described where all data underlying the findings will be made available when the study is complete?

The PLOS Data policy

Reviewer #1: Yes

Reviewer #2: No

**********

5. Is the manuscript presented in an intelligible fashion and written in standard English?

Reviewer #1: Yes

Reviewer #2: No

**********

Please use the space provided to explain your answers to the questions above and, if applicable, provide comments about issues authors must address before this protocol can be accepted for publication. You may also include additional comments for the author, including concerns about research or publication ethics.

You may also provide optional suggestions and comments to authors that they might find helpful in planning their study.

Reviewer #1: This protocol addresses a clinically relevant and understudied question in alveolar ridge augmentation. The rationale is well developed, the digital imaging workflow is validated, and the manuscript adheres to SPIRIT guidelines. The detailed surgical and imaging procedures is described sufficiently, and the registration and ethics documentation are complete.

However, several methodological and statistical issues are not clear and require revisions. These are important to be addressed to ensure internal validity, analytic transparency, and alignment between design and analysis.

1. Allocation method requires revision.

The use of a fixed rotating assignment schedule (A→B→C) creates predictability and risks selection bias. A computer-generated randomization sequence with variable block sizes (e.g., permuted blocks), prepared by an independent party and implemented with sequentially numbered, opaque, sealed envelopes, would ensure true allocation concealment.

2. Sample-size description is inconsistent and must be reconciled.

The abstract states 78 participants, while the methods describe a calculation yielding 15–18 per group and the eligibility criteria mention enrolling 40 adults. These numbers conflict. Please clarify:

the intended final sample size,

whether participants contribute one or two sites,

whether the unit of analysis is the participant or the site, and

how this is incorporated into the power calculation.

Alignment across the abstract, methods, and statistical section is needed.

3. Primary endpoint and primary model need explicit designation.

The manuscript describes the primary outcome as horizontal bone gain at 3, 6, and 12 months, yet the sample size appears based on a single 12-month comparison and the analysis plan mixes cross-sectional ANOVA with longitudinal mixed models. Please clearly specify:

the single primary endpoint (e.g., 12-month adjusted difference),

the primary analysis model (e.g., LMM with Group, Time, and Group×Time),

how non-inferiority will be formally tested at that endpoint, and

which analyses are exploratory.

4. Non-inferiority framework is insufficiently aligned with the planned analyses.

Although the study is framed as a non-inferiority trial with a defined margin, the proposed analysis (one-way ANOVA with Tukey tests) is inappropriate for non-inferiority. Non-inferiority requires CI-based testing comparing the lower CI bound to −Δ. Please revise the plan to reflect non-inferiority standards and ensure consistency between the margin, sample size justification, and statistical model.

5. Missing-data strategy needs further specification.

The manuscript states that multiple imputation will be used if missingness exceeds 10%, but does not specify:

the number of imputations,

variables included in the imputation model,

how imputations integrate with LMM analyses, or

planned sensitivity analyses (e.g., complete-case analysis or delta-adjusted imputation).

Providing these details will enhance transparency and reproducibility.

6. Blinding description should be clarified.

The manuscript currently describes the trial as double-blind, but surgeons cannot be blinded and participant blinding may not be feasible when autogenous harvesting is performed. Please clarify which parties are blinded (e.g., outcome assessors and statisticians) and adjust the blinding terminology accordingly.

7. Clustering must be consistently addressed across analyses.

Some participants may contribute two eligible sites, and while the LMM accounts for patient-level clustering, the planned cross-sectional ANOVA does not. Please state explicitly that primary analyses will use mixed-effects models including patient (and site, if applicable) as random effects.

8. Multiplicity across numerous secondary outcomes requires clarification.

The protocol lists multiple secondary outcomes at several time points without a clear plan for addressing multiplicity. Consider stating that secondary analyses will be exploratory and interpreted via effect sizes and confidence intervals, or predefine a structured approach (e.g., hierarchical sequence or FDR control).

Reviewer #2: This manuscript describes a randomized clinical trial protocol comparing autogenous, xenogeneic and allogeneic cortical shell techniques for horizontal ridge augmentation in the posterior mandible using an advanced digital and morphometric workflow. The topic is clinically relevant and timely. However, several important methodological inconsistencies and design issues must be addressed - the protocol must be considered robust and internally coherent.

Introduction vs Methods

In the Introduction the study is described as a “randomized, double-blind, split-mouth design”, whereas in the Methods it is consistently described as a parallel-group trial. This is a major conceptual inconsistency.

The randomization and intervention sections clearly indicate a parallel-group design.

The authors must correct the Introduction and all other sections to reflect the true design.

Methods

1. Three different sample sizes appear in the manuscript:

Abstract: 78 participants (26 per group)

Participants section: 40 adults

Sample size calculation section: 15 per group (with possible increase to 18 per group)

These figures are mutually incompatible.

So the authors must clearly state:

- the final planned sample size;

- the number per group;

- and align the Abstract, Methods and Sample size sections accordingly.

2. We also find inconsistency between study objective (non-inferiority) and statistical analysis.

The sample size section explicitly states that the study is powered as a non-inferiority trial (B vs A and C vs A). However, the statistical analysis plan states: one-way ANOVA with Tukey post-hoc tests, p < 0.05 for significance.

This corresponds to a superiority framework, not to a non-inferiority design.

This is a critical methodological issue.

3. The randomization method is not truly random

Participants are assigned following a fixed rotating sequence (A, B, C). This is not true randomization and can introduce allocation predictability, even with sealed envelopes.

4. Blinding is likely overstated

The study is described as double-blind, however: the surgeon cannot be blinded; the graft materials are visually and physically different; and the participants may easily be aware of donor-site harvesting in the autogenous group.

5. Primary outcome time point is unclear. The primary outcome is horizontal bone gain, but: the abstract refers to measurements at 3, 6 and 12 months, the sample size section specifies the primary endpoint at 12 months. So the manuscript should clearly define a single primary time point.

6. Use of Hounsfield Units for bone density in CBCT

Bone density is reported in Hounsfield Units derived from CBCT but it is well known that CBCT-derived gray values are not true HU and are highly device- and protocol-dependent.

7. The description of baseline ridge width as a covariate in ANCOVA is appropriate, but the method for baseline measurement (same CBCT protocol and reference lines) should be explicitly stated.

8. Postoperative inflammation outcome

It is measured as the linear distance from the lip commissure to the tragus.

However this outcome: is uncommon in the implant and augmentation literature; is highly operator- and positioning-dependent.

9. Exclusion of patients using hormonal contraceptives

Hormonal contraceptive use is listed as an exclusion criterion.

A justification should be provided, as this is not a standard exclusion criterion in oral bone-augmentation trials and may unnecessarily limit recruitment.

10. Smoking exclusion is defined as “>5 cigarettes/day”. Please clarify whether occasional or former smokers are eligible and how smoking status will be verified.

11. Ethics committee description

Two slightly different institutional descriptions appear for the same ethics committee. Why??

12. The protocol states that intraoral scans will be used for soft-tissue evaluation, but soft-tissue outcomes are not explicitly defined as endpoints. Please clarify their role (exploratory vs secondary outcome).

Formatting considerations

The manuscript contains several small formatting inconsistencies (spacing, line breaks, duplicated words such as “including” in the morphometric outcomes paragraph) that should be corrected.

**********

what does this mean?). If published, this will include your full peer review and any attached files.

If you choose “no”, your identity will remain anonymous but your review may still be made public.

**Do you want your identity to be public for this peer review?**  For information about this choice, including consent withdrawal, please see our Privacy Policy

Reviewer #1: No

Reviewer #2: No

**********

[NOTE: If reviewer comments were submitted as an attachment file, they will be attached to this email and accessible via the submission site. Please log into your account, locate the manuscript record, and check for the action link "View Attachments". If this link does not appear, there are no attachment files.]

To ensure your figures meet our technical requirements, please review our figure guidelines: https://journals.plos.org/plosone/s/figures

You may also use PLOS’s free figure tool, NAAS, to help you prepare publication quality figures: https://journals.plos.org/plosone/s/figures#loc-tools-for-figure-preparation.

NAAS will assess whether your figures meet our technical requirements by comparing each figure against our figure specifications.

# Author comment: Author response to Decision Letter 1

**DOI:** 10.1371/journal.pone.0337304.r002

19 Feb 2026

Editorial Requirement 1 – Formatting and Style

We have carefully revised the manuscript to ensure full compliance with PLOS ONE formatting and style requirements, including file naming conventions, title page structure, abstract formatting, and reference style. The manuscript has been aligned with the PLOS ONE formatting templates provided.

Editorial Requirement 2 – Competing Interests

We confirm that the Competing Interests statement has been completed on the online submission form as follows:

The authors have declared that no competing interests exist.

This statement has also been included in the cover letter, as requested.

Editorial Requirement 3 – Ethics-Approved Study Protocol

We have uploaded the complete ethics-approved study protocol as a Supporting Information file in its original language. An English translation has also been provided, in accordance with PLOS ONE guidelines for clinical trial protocols.

Editorial Requirement 4 – Suggested Citations

We carefully reviewed all reviewer-suggested references. Relevant publications have been incorporated where appropriate. No citations were added unless they were directly relevant to the study design or methodological framework.

Response to Reviewer #1 – Comment 1

We thank the reviewer for this important comment. We agree that a fixed rotating sequence could introduce allocation predictability. The manuscript has been revised to specify that allocation will be performed using a computer-generated randomization sequence created via Random.org, with variable block sizes and allocation concealment ensured through sequentially numbered, opaque, sealed envelopes (SNOSE), prepared by an independent researcher.

The description of rotating allocation has been removed entirely.

Reviewer #1 – Comment 2 (Sample-size description is inconsistent and must be reconciled)

We appreciate the reviewer’s careful reading. All inconsistencies have been corrected. The final planned sample size is 78 participants (26 per group), as stated in the Abstract, Participants section, and Sample Size section. The unit of analysis for the primary endpoint is the participant. When two sites are treated, a participant-level summary (mean horizontal gain) will be used.

The sample-size calculation is based on two pre-specified pairwise superiority comparisons (Group A vs B; Group A vs C), assuming:

• Pooled SD ≈ 1.12 mm,

• Clinically relevant difference = 1.0 mm,

• 80% power,

• Family-wise α = 0.05 with multiplicity adjustment.

All sections have been fully aligned.

Reviewer #1 – Comment 3 (Primary endpoint and primary model need explicit designation)

We have clarified that:

• The single confirmatory primary endpoint is horizontal bone gain (mm) at 6 months.

• The primary analysis model is ANCOVA with treatment group as fixed factor and baseline ridge width as covariate.

• The framework is superiority.

• Only two primary contrasts (A vs B; A vs C) are confirmatory.

• All longitudinal and other outcomes are explicitly labeled as secondary or exploratory.

All ambiguity regarding multiple primary time points has been removed.

Reviewer #1 – Comment 4 (Non-inferiority framework insufficiently aligned with planned analyses)

We thank the reviewer for identifying this issue. The study is no longer framed as a non-inferiority trial. The design has been revised to a superiority framework. All references to non-inferiority margins have been removed. The statistical plan now uses multiplicity-adjusted superiority testing (Holm procedure) for the two pre-specified primary comparisons.

Reviewer #1 – Comment 5 (Missing-data strategy requires specification)

We have expanded the missing-data section to fully specify:

• Threshold for imputation (>5% missingness),

• 20 imputations using MICE,

• Variables included in the imputation model (group, baseline ridge width, age, sex, smoking, post-baseline ridge width values, complications),

• Pooling via Rubin’s rules,

• Sensitivity analyses:

o complete-case analysis,

o delta-adjusted pattern-mixture analysis.

This has been fully detailed in the Statistical Analysis section.

Reviewer #1 – Comment 6 (Blinding description should be clarified)

The manuscript has been revised to state that this is a statistician-blinded, parallel-group trial.

We clarify that:

• The surgeon cannot be blinded.

• Participants cannot be fully blinded due to autogenous harvesting.

• The statistician remains blinded until completion of primary analysis.

All references to “double-blind” have been removed.

Reviewer #1 – Comment 7 (Clustering must be consistently addressed across analyses)

We have clarified that:

• The primary analysis is participant-level.

• When two sites are present, a participant-level summary is used.

• For site-level outcomes, mixed-effects models including Participant as a random effect will be applied.

• Longitudinal models include Participant (and Site nested within Participant when applicable) as random effects.

This has been explicitly described.

Reviewer #1 – Comment 8 (Multiplicity across secondary outcomes)

We now explicitly state that:

• Only the two primary contrasts are confirmatory.

• All secondary and exploratory outcomes will not undergo formal multiplicity correction.

• Results will be interpreted using effect sizes and confidence intervals rather than dichotomous significance testing.

Reviewer #2 – Comment 1 (Design inconsistency)

Thank you for identifying this important inconsistency. The study design has been clarified throughout the manuscript. The trial is a randomized, statistician-blinded, parallel-group clinical trial. All references to a split-mouth or double-blind design have been removed to ensure full conceptual coherence between the Introduction and Methods sections. The Introduction, Abstract, and Study Design sections have been revised to consistently reflect the true parallel-group allocation structure.

Reviewer #2 – Comment 2 (Sample size inconsistencies)

All inconsistencies have been corrected. The final planned sample size is 78 participants (26 per group), consistently reported across Abstract, Participants, and Sample Size sections.

Reviewer #2 – Comment 3 (Inconsistency between non-inferiority objective and statistical analysis)

The study is now explicitly framed as a superiority trial. All references to non-inferiority have been removed, and the statistical analysis plan has been fully aligned with this framework.

Reviewer #2 – Comment 4 (Randomization method is not truly random)

Thank you for this important methodological observation. The previous fixed rotating allocation sequence has been removed. The revised protocol now employs a computer-generated randomization sequence created through the web-based platform Random.org, using block randomization with variable block sizes to ensure both balance and unpredictability.

The allocation sequence will be generated by an independent researcher not involved in recruitment, surgery, or outcome assessment. Allocation concealment will be maintained using sequentially numbered, opaque, sealed envelopes (SNOSE), opened only after eligibility confirmation and informed consent. These revisions eliminate allocation predictability and ensure true randomization with appropriate concealment. The manuscript has been updated accordingly.

Reviewer #2 – Comment (Blinding)

Thank you for this important clarification. The study is not double-blind, and the manuscript has been revised to accurately describe the blinding structure. Due to the surgical nature of the interventions and the need for donor-site harvesting in the autogenous group, blinding of the surgeon and participants is not feasible. The trial is now clearly described as statistician-blinded, meaning that the statistician performing the primary analysis will remain blinded to group allocation through coded identifiers. All terminology suggesting double blinding has been removed to ensure transparency and methodological accuracy.

Reviewer #2 – Comment 5 (Primary outcome time point)

We thank the reviewer for identifying this inconsistency. The protocol has been revised to clearly define a single primary endpoint: horizontal bone gain at 6 months postoperatively. All sections (Abstract, Sample Size, and Statistical Analysis) have been aligned accordingly. Measurements at 3 and 12 months are now explicitly described as secondary exploratory outcomes.

Reviewer #2 – Comment 6 (Use of Hounsfield Units)

The manuscript has been revised to remove the term “Hounsfield Units (HU)” when referring to CBCT-derived measurements. Radiographic density will now be reported as device-specific gray values under standardized acquisition parameters.

Reviewer #2 – Comment 7 (Baseline ridge)

The manuscript has been revised to explicitly state that baseline ridge width will be measured from the preoperative CBCT using the same standardized reference lines (5, 7, and 11 mm from the crest) and identical measurement protocol as postoperative assessments.

Reviewer #2 – Comment 8 (Postoperative inflammation outcome)

We have clarified that linear facial measurements are exploratory and complemented by 3D structured-light facial scanning to improve objectivity and reproducibility.

Reviewer #2 – Comment 9 (Exclusion criteria)

We thank the reviewer for this observation. We agree that hormonal contraceptive use is not a standard exclusion criterion in bone-augmentation trials. After reconsideration, this criterion has been removed from the protocol. Only pregnancy and lactation are now listed as exclusion criteria due to surgical and radiographic considerations.

Reviewer #2 – Comment 10 (Smoking exclusion)

We clarified that:

• Only smokers with >5 cigarettes/day are excluded.

• Former smokers are eligible.

• Smoking status is assessed via structured self-report without biochemical verification.

This clarification has been incorporated into the Eligibility Criteria section.

Reviewer #2 – Comment 11 (Ethics committee)

The institutional name has been standardized throughout the manuscript to ensure consistency.

Reviewer #2 – Comment 12 (Soft-tissue outcomes)

We have clarified the role of intraoral scans in the revised manuscript. Soft-tissue contour changes derived from intraoral surface scans are now explicitly defined as exploratory secondary outcomes. These measurements will not be used for confirmatory hypothesis testing but will provide descriptive quantitative information regarding external volumetric and morphological remodeling. The Statistical Analysis section has been updated accordingly.

Attachment Submitted filename: Response to Reviewer.pdf

# Peer review: Decision Letter 1

**DOI:** 10.1371/journal.pone.0337304.r003

**Authors:** Carlos Alberto Viegas

17 Jun 2026

Dear Dr. Lanata-Flores,

Please submit your revised manuscript by Aug 01 2026 11:59PM. If you will need more time than this to complete your revisions, please reply to this message or contact the journal office at plosone@plos.org. When you're ready to submit your revision, log on to https://www.editorialmanager.com/pone/ and select the 'Submissions Needing Revision' folder to locate your manuscript file.

- A letter that responds to each point raised by the academic editor and reviewer(s). You should upload this letter as a separate file labeled 'Response to Reviewers'.
- A marked-up copy of your manuscript that highlights changes made to the original version. You should upload this as a separate file labeled 'Revised Manuscript with Track Changes'.
- An unmarked version of your revised paper without tracked changes. You should upload this as a separate file labeled 'Manuscript'.

If you would like to make changes to your financial disclosure, please include your updated statement in your cover letter. Guidelines for resubmitting your figure files are available below the reviewer comments at the end of this letter.

If applicable, we recommend that you deposit your laboratory protocols in protocols.io to enhance the reproducibility of your results. Protocols.io assigns your protocol its own identifier (DOI) so that it can be cited independently in the future. For instructions see: https://journals.plos.org/plosone/s/submission-guidelines#loc-laboratory-protocols. Additionally, PLOS ONE offers an option for publishing peer-reviewed Lab Protocol articles, which describe protocols hosted on protocols.io. Read more information on sharing protocols at https://plos.org/protocols?utm_medium=editorial-email&utm_source=authorletters&utm_campaign=protocols.

As the corresponding author, your ORCID iD is verified in the submission system and will appear in the published article. PLOS supports the use of ORCID, and we encourage all coauthors to register for an ORCID iD and use it as well. Please encourage your coauthors to verify their ORCID iD within the submission system before final acceptance, as unverified ORCID iDs will not appear in the published article. *Only*  the individual author can complete the verification step; PLOS staff *cannot*  verify ORCID iDs on behalf of authors.

We look forward to receiving your revised manuscript.

Kind regards,

Carlos Alberto Antunes Viegas, DVM; MSc; PhD

Academic Editor

PLOS One

Journal Requirements:

1. If the reviewer comments include a recommendation to cite specific previously published works, please review and evaluate these publications to determine whether they are relevant and should be cited. There is no requirement to cite these works unless the editor has indicated otherwise.

2. Please review your reference list to ensure that it is complete and correct. If you have cited papers that have been retracted, please include the rationale for doing so in the manuscript text, or remove these references and replace them with relevant current references. Any changes to the reference list should be mentioned in the rebuttal letter that accompanies your revised manuscript. If you need to cite a retracted article, indicate the article’s retracted status in the References list and also include a citation and full reference for the retraction notice.

[Note: HTML markup is below. Please do not edit.]

Reviewers' comments:

Reviewer's Responses to Questions

**Comments to the Author**

1. Does the manuscript provide a valid rationale for the proposed study, with clearly identified and justified research questions?

Reviewer #1: Yes

Reviewer #3: Yes

**********

2. Is the protocol technically sound and planned in a manner that will lead to a meaningful outcome and allow testing the stated hypotheses?

Reviewer #1: Partly

Reviewer #3: Yes

**********

3. Is the methodology feasible and described in sufficient detail to allow the work to be replicable?

Reviewer #1: No

Reviewer #3: Yes

**********

4. Have the authors described where all data underlying the findings will be made available when the study is complete?

The PLOS Data policy

Reviewer #1: Yes

Reviewer #3: Yes

**********

5. Is the manuscript presented in an intelligible fashion and written in standard English?

Reviewer #1: Yes

Reviewer #3: Yes

**********

Please use the space provided to explain your answers to the questions above and, if applicable, provide comments about issues authors must address before this protocol can be accepted for publication. You may also include additional comments for the author, including concerns about research or publication ethics.

You may also provide optional suggestions and comments to authors that they might find helpful in planning their study.

Reviewer #1: The revised protocol addresses several prior concerns, including clearer specification of a single primary endpoint at 6 months, participant-level inference when two sites are treated, and a stated primary ANCOVA framework with baseline adjustment. These are important improvements. However, the current revised document still contains multiple internal inconsistencies that must be corrected before the protocol can be considered sufficiently pre-specified and reproducible:

Randomization description remains contradictory. The manuscript includes both a computer-generated block randomization/SNOSE description and (elsewhere) the prior rotating A→B→C schedule text. Please remove the rotating-schedule language entirely and ensure the final clean manuscript contains only one allocation method, consistently described throughout.

Sample-size section is internally inconsistent regarding superiority vs non-inferiority. The text states the trial follows a superiority framework with no non-inferiority margin, but the same section still contains a non-inferiority sample-size paragraph (including one-sided alpha and hierarchical testing). Please fully align the sample size rationale with the final chosen framework (superiority), specify the confirmatory primary comparisons (e.g., A vs B; A vs C), and state clearly how multiplicity will be controlled for these primary contrasts. Also specify the assumed attrition rate (or expected loss to follow-up) used to justify the final n=78.

Statistical analysis section contains duplicated/contradictory methods and embedded reviewer text. Although the primary analysis is described as ANCOVA at 6 months, the same section also retains one-way ANOVA with Tukey post-hoc tests and repeated/duplicated LMM text, and it includes a pasted paragraph stating “Missing-data strategy needs further specification…”. Please remove the embedded reviewer paragraph and harmonize the analysis plan to one coherent primary approach (ANCOVA for the confirmatory endpoint at 6 months, with explicitly pre-specified primary contrasts and multiplicity adjustment), with secondary/exploratory models clearly labeled.

Missing-data plan is still not actually specified in the manuscript text. The protocol currently still states only that MI will be used if missingness exceeds 10%, without specifying number of imputations, imputation model variables, pooling (Rubin’s rules), or sensitivity analyses; moreover, the manuscript contains the pasted reviewer paragraph noting these omissions. Please replace this with a complete pre-specified approach (threshold for MI, number of imputations, method such as MICE, variables included, pooling rules, and at least one sensitivity analysis such as complete-case and/or pattern-mixture/delta adjustment).

Blinding statements remain inconsistent across document components. Parts of the submission still describe the trial as “double-blind,” while the revised abstract describes “statistician-blinded,” and the methods acknowledge image processing is not masked. Please ensure “double-blind” is removed everywhere and provide a consistent statement of who is blinded (statistician; ideally outcome assessors if feasible) and what safeguards will mitigate detection bias when image processing cannot be masked (e.g., pre-specified segmentation rules, calibration, blinded duplicate measurement, and reporting inter-/intra-rater reliability).

Reviewer #3: This is a well-structured, methodologically sound protocol for a randomized, statistician-blinded, parallel-group clinical trial comparing autogenous, xenogeneic, and allogeneic cortical shells for mandibular bone augmentation. The authors have comprehensively addressed previous reviewer comments, and the revised manuscript demonstrates significant improvements in clarity, consistency, and methodological rigour. The protocol is suitable for publication with minor revisions.

Recommendation: Minor revision

Major Concerns

1. The sample size calculation assumes a common SD of 1.2 mm, but the authors do not justify this value. While references 13 and 14 are cited, the specific SD values from these studies are not reported. The authors should state the actual SD values from the cited studies (e.g., "Lin et al. reported a pooled SD of X mm").

2. The primary endpoint is horizontal bone gain at 6 months, but the sample size calculation does not explicitly state that the 6-month time point was used for power calculations. Clarify that the 6-month assessment was used for the primary analysis.

3. The authors removed hormonal contraceptive use from exclusion criteria, which is appropriate. However, they should consider whether this is relevant for women of childbearing age and whether pregnancy testing is performed before surgery.

4. The study is registered at ClinicalTrials.gov (NCT06661447). The authors should confirm that the registration is complete and that all protocol details (primary outcome, sample size) match.

5. The surface registration workflow uses CloudCompare (open-source software). This is acceptable, but the authors should specify the software version and whether any custom scripts were used for batch processing.

6. The statistician-blinded design is appropriate. However, the authors should clarify who will break the blind if needed (e.g., in case of serious adverse events).

Minor Concerns

1. Abstract – the phrase "statistician-blinded" appears twice. Remove duplicate.

2. Abstract – the primary outcome is stated as "horizontal bone gain (mm) at 6 months". This is correct, but the introduction of the abstract also mentions 3, 6, and 12 months – clarify that only 6 months is confirmatory.

3. Methods – smoking exclusion: The authors state that "only current heavy smokers (>5 cigarettes/day) will be excluded" but do not specify how smoking status will be verified. Self-report is acceptable, but this should be stated.

4. Methods – postoperative facial swelling: The measurement from the oral commissure to the tragus is described. The authors should specify whether the same examiner performs all measurements to ensure reproducibility.

5. Results – expected results: The authors anticipate that xenogeneic and allogeneic laminae will achieve comparable outcomes to autogenous plates. The authors should consider whether a "non-inferiority" framework would be more appropriate for comparing prefabricated materials to the gold standard (autogenous) – but they have already addressed this and chosen superiority.

7. The authors should add a short statement on the expected clinical implications of the findings (e.g., "If prefabricated laminae achieve comparable results, they may reduce surgical time and morbidity, supporting their use as a first-line option").

**********

what does this mean?). If published, this will include your full peer review and any attached files.

If you choose “no”, your identity will remain anonymous but your review may still be made public.

**Do you want your identity to be public for this peer review?**  For information about this choice, including consent withdrawal, please see our Privacy Policy

Reviewer #1: No

Reviewer #3: No

**********

[NOTE: If reviewer comments were submitted as an attachment file, they will be attached to this email and accessible via the submission site. Please log into your account, locate the manuscript record, and check for the action link "View Attachments". If this link does not appear, there are no attachment files.]

To ensure your figures meet our technical requirements, please review our figure guidelines: https://journals.plos.org/plosone/s/figures

You may also use PLOS’s free figure tool, NAAS, to help you prepare publication quality figures: https://journals.plos.org/plosone/s/figures#loc-tools-for-figure-preparation.

NAAS will assess whether your figures meet our technical requirements by comparing each figure against our figure specifications.

# Author comment: Author response to Decision Letter 2

**DOI:** 10.1371/journal.pone.0337304.r004

16 Jul 2026

Manuscript Title:

Comparison of Bone Augmentation Techniques in the Mandibular Posterior Region: A Randomized Clinical Trial Protocol

Manuscript ID: PONE-D-25-58795R1

We sincerely thank the Editor and Reviewers for their valuable comments and suggestions. We have carefully revised the manuscript and addressed each point as detailed below.

Reviewer #1 – Comment 1

Randomization description remains contradictory. The manuscript includes both a computer-generated block randomization/SNOSE description and (elsewhere) the prior rotating A→B→C schedule text. Please remove the rotating-schedule language entirely and ensure the final clean manuscript contains only one allocation method, consistently described throughout.

Response:

We thank the reviewer for pointing this out. We removed the previous rotating A→B→C allocation text from the manuscript. The randomization method is now described consistently as computer-generated block randomization with a 1:1:1 allocation ratio, using sequentially numbered, opaque, sealed envelopes (SNOSE) for allocation concealment.

We also clarified that, when a participant has two eligible posterior mandibular sites, both sites will receive the same allocated intervention. This is consistent with the parallel-group design and the participant-level primary analysis.

Location of changes:

• Materials and Methods section, “Study groups and interventions” subsection.

• Materials and Methods section, “Randomization and blinding” subsection.

Reviewer #1 – Comment 2

Sample-size section is internally inconsistent regarding superiority vs non-inferiority. The text states the trial follows a superiority framework with no non-inferiority margin, but the same section still contains a non-inferiority sample-size paragraph (including one-sided alpha and hierarchical testing). Please fully align the sample size rationale with the final chosen framework (superiority), specify the confirmatory primary comparisons (e.g., A vs B; A vs C), and state clearly how multiplicity will be controlled for these primary contrasts. Also specify the assumed attrition rate (or expected loss to follow-up) used to justify the final n=78.

Response:

We thank the reviewer for this important observation. The sample-size section has been revised to fully align the protocol with the final superiority framework. All non-inferiority language, including any reference to a non-inferiority margin, one-sided alpha level, or hierarchical non-inferiority testing, has been removed.

We now specify that the single confirmatory primary endpoint is participant-level horizontal bone gain at 6 months. The two confirmatory primary comparisons are Group A (autogenous cortical shell) versus Group B (xenogeneic cortical shell), and Group A versus Group C (allogeneic cortical shell). The Group B versus Group C comparison will be considered secondary and exploratory. Multiplicity across the two confirmatory primary contrasts will be controlled using the Holm–Bonferroni procedure, maintaining a two-sided family-wise alpha level of 0.05.

We also clarified that the final sample size of 78 participants includes an anticipated 10% loss to follow-up before the 6-month CBCT assessment, while preserving equal allocation across the three groups.

Location of changes:

• Materials and Methods section, “Sample size” subsection.

• Materials and Methods section, “Statistical analysis – Primary and secondary endpoints” subsection.

Reviewer #1 – Comment 3

Statistical analysis section contains duplicated/contradictory methods and embedded reviewer text. Although the primary analysis is described as ANCOVA at 6 months, the same section also retains one-way ANOVA with Tukey post-hoc tests and repeated/duplicated LMM text, and it includes a pasted paragraph stating “Missing-data strategy needs further specification…”. Please remove the embedded reviewer paragraph and harmonize the analysis plan to one coherent primary approach (ANCOVA for the confirmatory endpoint at 6 months, with explicitly pre-specified primary contrasts and multiplicity adjustment), with secondary/exploratory models clearly labeled.

Response:

We thank the reviewer for noting this issue. We revised the statistical analysis section to remove duplicated and contradictory text while keeping the primary analysis consistent with the planned ANCOVA framework.

The primary endpoint is now described as participant-level horizontal bone gain at 6 months, analyzed using ANCOVA with treatment group as the fixed factor and baseline ridge width as a covariate. We also clarified that the two confirmatory primary contrasts will be Group A versus Group B and Group A versus Group C, with Holm–Bonferroni adjustment for multiplicity.

The reviewer comment that had inadvertently remained in the manuscript was removed. Secondary longitudinal, morphometric, categorical, and soft-tissue analyses are now described as secondary or exploratory.

Location of changes:

• Materials and Methods section, “Statistical analysis – Primary and secondary endpoints” subsection.

Reviewer #1 – Comment 4

Missing-data plan is still not actually specified in the manuscript text. The protocol currently still states only that MI will be used if missingness exceeds 10%, without specifying number of imputations, imputation model variables, pooling (Rubin’s rules), or sensitivity analyses; moreover, the manuscript contains the pasted reviewer paragraph noting these omissions. Please replace this with a complete pre-specified approach (threshold for MI, number of imputations, method such as MICE, variables included, pooling rules, and at least one sensitivity analysis such as complete-case and/or pattern-mixture/delta adjustment).

Response:

We thank the reviewer for this important comment. We added a dedicated missing-data section to the statistical analysis plan.

The revised text now specifies that the extent and reasons for missing data will be described by treatment group. If missing data for the primary endpoint exceed 10%, multiple imputation will be performed using multivariate imputation by chained equations (MICE), with 20 imputed datasets. The imputation model will include treatment group, baseline ridge width, age, sex, smoking status, number of treated sites, and available horizontal bone gain measurements at 3, 6, and 12 months. Estimates and standard errors from the imputed datasets will be combined according to Rubin’s rules, and the corresponding reference was added to the reference list. We also added complete-case and delta-adjusted sensitivity analyses to assess the robustness of the primary endpoint results.

Location of changes:

• Materials and Methods section, “Statistical analysis – Missing data” subsection.

• References section.

Reviewer #1 – Comment 5

Blinding statements remain inconsistent across document components. Parts of the submission still describe the trial as “double-blind,” while the revised abstract describes “statistician-blinded,” and the methods acknowledge image processing is not masked. Please ensure “double-blind” is removed everywhere and provide a consistent statement of who is blinded (statistician; ideally outcome assessors if feasible) and what safeguards will mitigate detection bias when image processing cannot be masked (e.g., pre-specified segmentation rules, calibration, blinded duplicate measurement, and reporting inter-/intra-rater reliability).

Response:

We thank the reviewer for pointing out this inconsistency. We revised the manuscript to remove references to “double-blind” and other unsupported blinding descriptions.

The trial is now described consistently as a randomized, statistician-blinded, parallel-group clinical trial. We clarified that the statistician responsible for the primary analysis will remain blinded to treatment allocation through coded group identifiers until completion of the primary analysis. We also state that blinding of the operating surgeon and participants is not feasible because of the surgical nature of the interventions and the need for autogenous bone harvesting in Group A.

We further clarified how detection bias will be minimized during outcome assessment. The revised manuscript now specifies the use of standardized CBCT segmentation and surface-registration protocols, pre-specified segmentation rules, examiner calibration, duplicate measurements, and reporting of intra- and inter-examiner reliability using intraclass correlation coefficients.

Location of changes:

• Abstract.

• Materials and Methods section, “Randomization and blinding” subsection.

• Materials and Methods section, “Volumetric and morphometric analysis” subsection.

Reviewer #3 – Comment 1

The sample size calculation assumes a common SD of 1.2 mm, but the authors do not justify this value. While references 13 and 14 are cited, the specific SD values from these studies are not reported. The authors should state the actual SD values from the cited studies (e.g., "Lin et al. reported a pooled SD of X mm")

Response:

We thank the reviewer for this helpful comment. The sample-size section has been revised to report the specific standard deviation values from the studies used to support the assumed common SD of 1.2 mm.

We now state that Lin et al. reported horizontal ridge resorption at 1 mm from the crest of 1.29 ± 1.37 mm, 1.07 ± 1.07 mm, and 1.54 ± 1.33 mm across the three study groups at 6 months, corresponding to an approximate pooled SD of 1.26 mm. We also state that Kim et al. reported horizontal width changes at 3 mm from the crest of −0.37 ± 1.31 mm, 0.10 ± 0.97 mm, and 0.72 ± 0.80 mm across groups, corresponding to an approximate pooled SD of 1.05 mm.

Based on these values, the assumed common SD of 1.2 mm was retained as a reasonable estimate for the present trial.

Location of changes:

• Materials and Methods section, “Sample size” subsection.

Reviewer #3 – Comment 2

The primary endpoint is horizontal bone gain at 6 months, but the sample size calculation does not explicitly state that the 6-month time point was used for power calculations. Clarify that the 6-month assessment was used for the primary analysis.

Response:

We thank the reviewer for this comment. We clarified in the sample-size section that the primary endpoint used for the sample-size calculation is participant-level horizontal bone gain at 6 months, measured by CBCT. We also specified that the 6-month assessment was selected a priori as the definitive confirmatory time point for hypothesis testing.

Location of changes:

• Materials and Methods section, “Sample size” subsection.

Reviewer #3 – Major Concern 3

The authors removed hormonal contraceptive use from exclusion criteria, which is appropriate. However, they should consider whether this is relevant for women of childbearing age and whether pregnancy testing is performed before surgery.

Response:

We thank the reviewer for this comment. We agree that hormonal contraceptive use should not be an exclusion criterion by itself. We clarified that, for women of childbearing age, possible pregnancy will be assessed by self-report before surgery. If pregnancy status is uncertain, the participant may either be excluded from the study or undergo a urine pregnancy test before surgical intervention. Pregnant or lactating women remain excluded from participation.

Location of changes:

• Materials and Methods section, “Participants” subsection.

Reviewer #3 – Major Concern 4

The study is registered at ClinicalTrials.gov (NCT06661447). The authors should confirm that the registration is complete and that all protocol details (primary outcome, sample size) match.

Response:

We thank the reviewer for this comment. We reviewed the ClinicalTrials.gov registration and updated the relevant protocol details to match the revised manuscript, including the primary outcome, the 6-month confirmatory time point, the planned sample size of 78 participants, and the parallel-group allocation.

The ClinicalTrials.gov registration is complete and now matches the revised protocol.

Location of changes:

• ClinicalTrials.gov registration record NCT06661447.

Reviewer #3 – Major Concern 5

The surface registration workflow uses CloudCompare (open-source software). This is acceptable, but the authors should specify the software version and whether any custom scripts were used for batch processing.

Response:

We thank the reviewer for this comment. We revised the surface-registration workflow description to specify the CloudCompare version used for the analysis. We also clarified that no custom scripts will be used for batch processing and that registration and measurement procedures will be performed using the standard graphical user interface tools available in CloudCompare.

Location of changes:

• Materials and Methods section, “Volumetric and morphometric analysis – Surface registration and quantitative analysis” subsection.

Reviewer #3 – Major Concern 6

The statistician-blinded design is appropriate. However, the authors should clarify who will break the blind if needed, for example in case of serious adverse events.

Response:

We thank the reviewer for this comment. We clarified the unblinding procedure in the Randomization and blinding section. If unblinding is required for participant safety, such as in the event of a serious adverse event, the principal investigator will authorize and document the unblinding process.

Location of changes:

• Materials and Methods section, “Randomization and blinding” subsection.

Reviewer #3 – Minor Concern 1

Abstract – the phrase “statistician-blinded” appears twice. Remove duplicate.

Response:

We thank the reviewer for noting this. We reviewed the Abstract and removed the duplicate wording. The trial is now described once as a randomized, statistician-blinded, parallel-group clinical trial.

Location of changes:

• Abstract.

Reviewer #3 – Minor Concern 2

Abstract – the primary outcome is stated as “horizontal bone gain (mm) at 6 months”. This is correct, but the introduction of the abstract also mentions 3, 6, and 12 months – clarify that only 6 months is confirmatory.

Response:

We thank the reviewer for this comment. We clarified in the Abstract that horizontal bone gain at 6 months is the single primary endpoint. The 3- and 12-month CBCT assessments are now described as secondary longitudinal outcomes.

Location of changes:

• Abstract.

Reviewer #3 – Minor Concern 3

Methods – smoking exclusion: The authors state that “only current heavy smokers (>5 cigarettes/day) will be excluded” but do not specify how smoking status will be verified. Self-report is acceptable, but this should be stated.

Response:

We thank the reviewer for this observation. We clarified that smoking status will be assessed at baseline by structured self-report and categorized as current heavy smokers, light smokers, former smokers, or never smokers. Smoking history and vaping use will also be recorded for descriptive and exploratory analyses.

Location of changes:

• Materials and Methods section, “Participants” subsection.

Reviewer #3 – Minor Concern 4

Methods – postoperative facial swelling: The measurement from the oral commissure to the tragus is described. The authors should specify whether the same examiner performs all measurements to ensure reproducibility.

Response:

We thank the reviewer for this comment. We clarified that postoperative facial swelling measurements will be performed twice by the same calibrated examiner, and the mean value will be recorded to improve reproducibility.

Location of changes:

• Materials and Methods section, “Surgical procedure” subsection.

Reviewer #3 – Minor Concern 5

Results – expected results: The authors anticipate that xenogeneic and allogeneic laminae will achieve comparable outcomes to autogenous plates. The authors should consider whether a “non-inferiority” framework would be more appropriate for comparing prefabricated materials to the gold standard (autogenous), but they have already addressed this and chosen superiority.

Response:

We thank the reviewer for this thoughtful comment. We considered this point carefully and have retained a superiority framework for the present protocol. To avoid ambiguity, the sample-size and statistical-analysis sections were revised to remove non-inferiority language and to state the confirmatory primary comparisons and multiplicity adjustment clearly.

Location of changes:

• Materials and Methods section, “Sample size” and “Statistical analysis” subsections.

Reviewer #3 – Minor Concern 7

The authors should add a short statement on the expected clinical implications of the findings.

Response:

We thank the reviewer for this suggestion. We added a short statement on the expected clinical implications of the study findings. Specifically, we note that if prefabricated xenogeneic or allogeneic laminae show comparable clinical and volumetric performance to autogenous cortical shells, they may help reduce donor-site morbidity and surgical time and support their use as alternative treatment options for posterior mandibular ridge augmentation.

Location of changes:

• Expected Results and Discussion section.

Attachment Submitted filename: Response to Reviewers.pdf

# Peer review: Decision Letter 2

**DOI:** 10.1371/journal.pone.0337304.r005

**Authors:** Carlos Alberto Viegas

27 Jul 2026

Thank you for submitting your manuscript to PLOS ONE. After careful consideration, we feel that it has merit but does not fully meet PLOS ONE’s publication criteria as it currently stands. Therefore, we invite you to submit a revised version of the manuscript that addresses the points raised during the review process.

Please submit your revised manuscript by Sep 10 2026 11:59PM. If you will need more time than this to complete your revisions, please reply to this message or contact the journal office at plosone@plos.org. When you're ready to submit your revision, log on to https://www.editorialmanager.com/pone/ and select the 'Submissions Needing Revision' folder to locate your manuscript file.

- A letter that responds to each point raised by the academic editor and reviewer(s). You should upload this letter as a separate file labeled 'Response to Reviewers'.
- A marked-up copy of your manuscript that highlights changes made to the original version. You should upload this as a separate file labeled 'Revised Manuscript with Track Changes'.
- An unmarked version of your revised paper without tracked changes. You should upload this as a separate file labeled 'Manuscript'.

If you would like to make changes to your financial disclosure, please include your updated statement in your cover letter. Guidelines for resubmitting your figure files are available below the reviewer comments at the end of this letter.

If applicable, we recommend that you deposit your laboratory protocols in protocols.io to enhance the reproducibility of your results. Protocols.io assigns your protocol its own identifier (DOI) so that it can be cited independently in the future. For instructions see: https://journals.plos.org/plosone/s/submission-guidelines#loc-laboratory-protocols. Additionally, PLOS ONE offers an option for publishing peer-reviewed Lab Protocol articles, which describe protocols hosted on protocols.io. Read more information on sharing protocols at https://plos.org/protocols?utm_medium=editorial-email&utm_source=authorletters&utm_campaign=protocols.

As the corresponding author, your ORCID iD is verified in the submission system and will appear in the published article. PLOS supports the use of ORCID, and we encourage all coauthors to register for an ORCID iD and use it as well. Please encourage your coauthors to verify their ORCID iD within the submission system before final acceptance, as unverified ORCID iDs will not appear in the published article. *Only*  the individual author can complete the verification step; PLOS staff *cannot*  verify ORCID iDs on behalf of authors.

We look forward to receiving your revised manuscript.

Kind regards,

Carlos Alberto Antunes Viegas, DVM; MSc; PhD

Academic Editor

PLOS One

**Journal Requirements:**

If the reviewer comments include a recommendation to cite specific previously published works, please review and evaluate these publications to determine whether they are relevant and should be cited. There is no requirement to cite these works unless the editor has indicated otherwise.

[Note: HTML markup is below. Please do not edit.]

Reviewers' comments:

Reviewer's Responses to Questions

**Comments to the Author**

1. Does the manuscript provide a valid rationale for the proposed study, with clearly identified and justified research questions?

Reviewer #1: Yes

Reviewer #3: Yes

**********

2. Is the protocol technically sound and planned in a manner that will lead to a meaningful outcome and allow testing the stated hypotheses?

Reviewer #1: No

Reviewer #3: Yes

**********

3. Is the methodology feasible and described in sufficient detail to allow the work to be replicable?

Reviewer #1: No

Reviewer #3: Yes

**********

4. Have the authors described where all data underlying the findings will be made available when the study is complete?

The PLOS Data policy

Reviewer #1: No

Reviewer #3: Yes

**********

5. Is the manuscript presented in an intelligible fashion and written in standard English?

Reviewer #1: Yes

Reviewer #3: Yes

**********

Please use the space provided to explain your answers to the questions above and, if applicable, provide comments about issues authors must address before this protocol can be accepted for publication. You may also include additional comments for the author, including concerns about research or publication ethics.

You may also provide optional suggestions and comments to authors that they might find helpful in planning their study.

Reviewer #1: The authors have made substantial improvements in Revision 2. The randomization procedure is now consistently described, the primary endpoint is clearly defined as participant-level horizontal bone gain at 6 months, the primary ANCOVA model and two confirmatory contrasts are identified, and the Holm procedure is specified. The previous noninferiority text, duplicated analyses, and inadvertently embedded reviewer comments have also been removed. The additions concerning the source of the assumed standard deviation, missing-data procedures, examiner calibration, reliability assessment, and software version are helpful. Nevertheless, several important statistical issues remain and should be addressed before the protocol is accepted.

1. Superiority framework remains inconsistent with the scientific objective

The protocol is now formally described as a superiority trial. However, the Introduction and Expected Results continue to state that xenogeneic and allogeneic laminae are expected to produce “comparable” or “similar” outcomes to autogenous cortical shells. A superiority analysis cannot establish comparability. Failure to detect a statistically significant difference does not demonstrate equivalence or noninferiority. The authors should either:

* Retain the superiority framework and rewrite the objectives, hypotheses, expected results, and clinical implications as testing for differences among treatments; or

* Adopt a properly designed noninferiority framework with a clinically justified noninferiority margin.

If the intended clinical question is whether prefabricated laminae preserve bone gain while reducing donor-site morbidity or surgical time, a noninferiority hypothesis for bone gain, potentially accompanied by superiority analyses for morbidity or operative time, may be more closely aligned with that objective. Regardless of the selected framework, the statistical hypotheses and permissible conclusions must be consistent throughout the manuscript.

2. Sample-size calculation remains insufficiently reproducible

The revised section states that 69 evaluable participants provide at least 80% power to detect a 1.0-mm difference with a common SD of 1.2 mm, followed by inflation to 78 participants for 10% attrition. However, the calculation is not fully aligned with the two confirmatory Holm-adjusted contrasts. With 69 evaluable participants, approximately 23 participants would remain per group. A conventional two-group approximation with a 1.0-mm difference and SD of 1.2 mm provides approximately 79% power at two-sided α=0.05, but only approximately 69% power at two-sided α=0.025, a conservative multiplicity-adjusted level for two primary contrasts. ANCOVA may improve power, but the assumed baseline–outcome correlation or expected variance reduction has not been stated.

Please provide:

* The software and procedure used for the calculation;

* The assumed group means or contrast-specific alternatives;

* How Holm multiplicity adjustment was incorporated;

* Whether the stated power refers to each contrast, at least one contrast, or joint rejection;

* The assumed correlation between baseline ridge width and the 6-month outcome, if ANCOVA efficiency was incorporated; and

* The resulting power for each confirmatory contrast after attrition.

The Abstract’s claim of “80–90% power” should be corrected because the current Methods section supports neither that range nor a 90% power calculation. Although the cited studies now provide empirical SD values, their measurements differ from the proposed primary outcome in anatomical level and clinical setting. Please discuss this transportability and consider a sensitivity analysis across plausible SD values.

3. Missing-data plan remains partly data-dependent and incomplete

The added information regarding MICE, 20 imputations, imputation-model variables, Rubin’s rules, complete-case analysis, and delta adjustment is useful. However, the primary analysis is described as including only randomized participants with available 6-month CBCT data, with multiple imputation used only if missingness exceeds 10%.

This approach makes the analysis method depend on the observed missing-data percentage. Even less than 10% missingness can produce bias if missingness is related to prognosis or treatment. Please prespecify one primary approach that does not depend on an arbitrary observed threshold. A reasonable option would be multiple imputation under the missing-at-random assumption whenever primary-outcome data are missing, with complete-case analysis as a sensitivity analysis.

The delta-adjusted analysis is not reproducible without specifying:

* Which treatment group or groups receive the delta adjustment;

* The numerical delta values or range;

* The direction of the assumed unfavorable departure; and

* Whether a tipping-point analysis will be performed.

The protocol should also explain how missing observations will be handled in secondary longitudinal mixed-effects analyses.

4. Secondary-outcome analyses remain under-specified

The manuscript lists several secondary outcomes but does not provide a complete analysis method for each. In particular, the plan remains unclear for:

* Volumetric change;

* Relative CBCT gray values;

* Surgical duration;

* Repeated postoperative facial-swelling measurements; and

* Soft-tissue contour changes.

Please provide an outcome-by-outcome analysis plan specifying the analysis unit, time point, model, treatment effect, confidence interval, and handling of repeated or bilateral measurements.

For the longitudinal horizontal bone-gain model, please explicitly specify treatment group, time, and group-by-time interaction as fixed effects; any baseline adjustment; the random-effects structure; and the covariance structure. Model assumptions should be assessed using model residuals rather than relying solely on a Shapiro–Wilk test of the raw outcome data.

5. Outcome-assessor masking and measurement reliability require clarification

The removal of “double-blind” terminology is appropriate, and the addition of standardized segmentation rules, duplicate measurements, examiner calibration, and ICC reporting reduces detection-bias concerns. Nevertheless, the protocol does not clearly state whether the CBCT segmenters and primary-outcome examiners will be blinded to treatment assignment and imaging time point.

Please state explicitly:

* Which outcome assessors will be blinded;

* Whether graft materials can be identified from the images;

* How scans will be coded and ordered;

* Whether duplicate measurements will be performed independently; and

* What corrective action will be taken if the prespecified reliability criterion is not achieved.

If outcome-assessor masking is not feasible, this limitation should be stated clearly.

6. Data-availability plan

The manuscript states that de-identified data will be available upon reasonable request to the corresponding author. This alone does not satisfy the PLOS Data Policy. Please identify an appropriate public repository and commit to depositing the de-identified participant-level data underlying the reported results, together with the data dictionary, metadata, and statistical code.

If legal or ethical restrictions prevent public release of participant-level data, specify the restriction, the responsible institutional contact or data-access committee, the application procedure, and the criteria governing access. Data access should not depend solely on the corresponding author’s discretion.

7. Final consistency checks

Please ensure that:

* The “80–90% power” statement is corrected throughout;

* All claims of “comparable” or “similar” performance are aligned with the final inferential framework;

* The final statistical analysis plan, ethics-approved protocol, manuscript, and ClinicalTrials.gov record contain identical primary endpoint, time point, sample size, treatment groups, and analysis framework; and

* Any changes are prospectively registered and approved before recruitment begins.

I think that overall revision 2 addresses many of the previous inconsistencies and represents meaningful progress. However, the above issues, including superiority–comparability conflict, sample-size calculation, missing-data strategy, secondary-outcome models, and outcome-assessor masking, remain insufficiently resolved.

Reviewer #3: Accept – The manuscript is methodologically sound, transparent, and suitable for publication. Minor formatting corrections (removing formatting notes, streamlining redundant text) can be addressed during production

**********

what does this mean?). If published, this will include your full peer review and any attached files.

If you choose “no”, your identity will remain anonymous but your review may still be made public.

**Do you want your identity to be public for this peer review?**  For information about this choice, including consent withdrawal, please see our Privacy Policy

Reviewer #1: No

Reviewer #3: No

**********

[NOTE: If reviewer comments were submitted as an attachment file, they will be attached to this email and accessible via the submission site. Please log into your account, locate the manuscript record, and check for the action link "View Attachments". If this link does not appear, there are no attachment files.]

To ensure your figures meet our technical requirements, please review our figure guidelines: https://journals.plos.org/plosone/s/figures

You may also use PLOS’s free figure tool, NAAS, to help you prepare publication quality figures: https://journals.plos.org/plosone/s/figures#loc-tools-for-figure-preparation.

NAAS will assess whether your figures meet our technical requirements by comparing each figure against our figure specifications.

# Author comment: Author response to Decision Letter 3

**DOI:** 10.1371/journal.pone.0337304.r006

5 Oct 2026

We have revised the manuscript in response to the remaining comments from Reviewer 1. A detailed point-by-point response has been uploaded as a separate file labeled “Response to Reviewers.”

In brief, we aligned the manuscript consistently with the superiority framework, revised the sample-size rationale and Supplementary File 1, clarified the missing-data plan, expanded the secondary-outcome analysis plan, clarified outcome-assessor masking and reliability procedures, revised the data-availability statement, and completed final consistency checks across the manuscript.

Attachment Submitted filename: Response_to_Reviewers_auresp_3.pdf

# Peer review: Decision Letter 3

**DOI:** 10.1371/journal.pone.0337304.r007

**Authors:** Carlos Alberto Viegas

8 Oct 2026

Comparison of Bone Augmentation Techniques in the Mandibular Posterior Region: A Randomized Clinical Trial Protocol

PONE-D-25-58795R3

Dear Dr. António Lanata-Flores,

We’re pleased to inform you that your manuscript has been judged scientifically suitable for publication and will be formally accepted for publication once it meets all outstanding technical requirements.

Within one week, you’ll receive an e-mail detailing the required amendments. When these have been addressed, you’ll receive a formal acceptance letter and your manuscript will be scheduled for publication.

An invoice will be generated when your article is formally accepted. Please note, if your institution has a publishing partnership with PLOS and your article meets the relevant criteria, all or part of your publication costs will be covered. Please make sure your user information is up-to-date by logging into Editorial Manager at Editorial Manager® and clicking the ‘Update My Information' link at the top of the page. For questions related to billing, please contact billing support.

If your institution or institutions have a press office, please notify them about your upcoming paper to help maximize its impact. If they’ll be preparing press materials, please inform our press team as soon as possible -- no later than 48 hours after receiving the formal acceptance. Your manuscript will remain under strict press embargo until 2 pm Eastern Time on the date of publication. For more information, please contact onepress@plos.org.

Kind regards,

Carlos Alberto Antunes Viegas, DVM; MSc; PhD

Academic Editor

PLOS One

Additional Editor Comments (optional):

Reviewers' comments:

Reviewer's Responses to Questions

**Comments to the Author**

1. Does the manuscript provide a valid rationale for the proposed study, with clearly identified and justified research questions?

Reviewer #1: Yes

**********

2. Is the protocol technically sound and planned in a manner that will lead to a meaningful outcome and allow testing the stated hypotheses?

Reviewer #1: Yes

**********

3. Is the methodology feasible and described in sufficient detail to allow the work to be replicable?

Reviewer #1: Yes

**********

4. Have the authors described where all data underlying the findings will be made available when the study is complete?

The PLOS Data policy

Reviewer #1: Yes

**********

5. Is the manuscript presented in an intelligible fashion and written in standard English?

Reviewer #1: Yes

**********

Please use the space provided to explain your answers to the questions above and, if applicable, provide comments about issues authors must address before this protocol can be accepted for publication. You may also include additional comments for the author, including concerns about research or publication ethics.

You may also provide optional suggestions and comments to authors that they might find helpful in planning their study.

Reviewer #1: Thank you for taking time for the revision. All of my comments and concerns have bee addressed; and I do not have any additional questions.

**********

what does this mean?). If published, this will include your full peer review and any attached files.

If you choose “no”, your identity will remain anonymous but your review may still be made public.

**Do you want your identity to be public for this peer review?**  For information about this choice, including consent withdrawal, please see our Privacy Policy

Reviewer #1: No

**********

# Editor report: Acceptance letter

**DOI:** 10.1371/journal.pone.0337304.r008

**Authors:** Carlos Alberto Viegas

PONE-D-25-58795R3

PLOS One

Dear Dr. Lanata-Flores,

I'm pleased to inform you that your manuscript has been deemed suitable for publication in PLOS One. Congratulations! Your manuscript is now being handed over to our production team.

At this stage, our production department will prepare your paper for publication. This includes ensuring the following:

* All references, tables, and figures are properly cited

* All relevant supporting information is included in the manuscript submission,

* There are no issues that prevent the paper from being properly typeset

You will receive further instructions from the production team, including instructions on how to review your proof when it is ready. Please keep in mind that we are working through a large volume of accepted articles, so please give us a few days to review your paper and let you know the next and final steps.

Lastly, if your institution or institutions have a press office, please let them know about your upcoming paper now to help maximize its impact. If they'll be preparing press materials, please inform our press team within the next 48 hours. Your manuscript will remain under strict press embargo until 2 pm Eastern Time on the date of publication. For more information, please contact onepress@plos.org.

You will receive an invoice from PLOS for your publication fee after your manuscript has reached the completed accept phase. If you receive an email requesting payment before acceptance or for any other service, this may be a phishing scheme. Learn how to identify phishing emails and protect your accounts at https://explore.plos.org/phishing.

If we can help with anything else, please email us at customercare@plos.org.

Thank you for submitting your work to PLOS One and supporting open access.

Kind regards,

PLOS One Editorial Office Staff

on behalf of

Dr. Carlos Alberto Antunes Viegas

Academic Editor

PLOS One
